# Molecular subtyping reveals immune alterations associated with progression of bronchial premalignant lesions

**DOI:** 10.1038/s41467-019-09834-2

**Published:** 2019-04-23

**Authors:** Jennifer E. Beane, Sarah A. Mazzilli, Joshua D. Campbell, Grant Duclos, Kostyantyn Krysan, Christopher Moy, Catalina Perdomo, Michael Schaffer, Gang Liu, Sherry Zhang, Hanqiao Liu, Jessica Vick, Samjot S. Dhillon, Suso J. Platero, Steven M. Dubinett, Christopher Stevenson, Mary E. Reid, Marc E. Lenburg, Avrum E. Spira

**Affiliations:** 1 Boston University School of Medicine, Boston, MA 02118 USA; 2 David Geffen School of Medicine at UCLA, Los Angeles, CA 90095 USA; 3 Johnson and Johnson Innovation, Cambridge, MA 02142 USA; 4 Princeton University, Princeton, NJ 08544 USA; 5 Kaiser Permanente, Roseville and Sacramento, Roseville, CA USA; 6 Covance, Princeton, NJ 08540 USA; 7 Janssen Research and Development, High Wycombe, HP12 4DP UK; 8 Roswell Park Comprehensive Cancer Center, Buffalo, NY 14203 USA

**Keywords:** Lung cancer, Tumour biomarkers, Computational biology and bioinformatics

## Abstract

Bronchial premalignant lesions (PMLs) are precursors of lung squamous cell carcinoma, but have variable outcome, and we lack tools to identify and treat PMLs at risk for progression to cancer. Here we report the identification of four molecular subtypes of PMLs with distinct differences in epithelial and immune processes based on RNA-Seq profiling of endobronchial biopsies from high-risk smokers. The Proliferative subtype is enriched with bronchial dysplasia and exhibits up-regulation of metabolic and cell cycle pathways. A Proliferative subtype-associated gene signature identifies subjects with Proliferative PMLs from normal-appearing uninvolved large airway brushings with high specificity. In progressive/persistent Proliferative lesions expression of interferon signaling and antigen processing/presentation pathways decrease and immunofluorescence indicates a depletion of innate and adaptive immune cells compared with regressive lesions. Molecular biomarkers measured in PMLs or the uninvolved airway can enhance histopathological grading and suggest immunoprevention strategies for intercepting the progression of PMLs to lung cancer.

## Abstract (web summary)

Bronchial premalignant lesions can potentially progress to lung squamous cell carcinoma. Here, the authors profile bronchial biopsies from high-risk smokers by RNA sequencing and identify four molecular subtypes of premalignant lesions and an immune molecular signature that associates with lesion progression.

## Introduction

Lung cancer (LC) is the leading cause of cancer death taking ~160,000 US lives each year, more than colorectal, pancreatic, breast, and prostate cancers combined. To decrease mortality, we need innovative strategies to intercept cancer development by diagnosing the disease at its earliest and potentially most curable stage. Development of LC risk biomarkers and interception strategies requires a detailed understanding of the earliest molecular alterations involved in lung carcinogenesis that occur in the respiratory epithelium^1,2^. Exposure to cigarette smoke creates a field of injury throughout the entire respiratory tract by inducing a variety of genomic alterations that can lead to an at-risk airway where premalignant lesions (PMLs) and LCs develop. Lung squamous cell carcinoma (LUSC) arises in the epithelial layer of the bronchial airways and is often preceded by the development of PMLs through a stepwise histological progression from normal epithelium to hyperplasia, squamous metaplasia, dysplasia (mild, moderate, and severe), carcinoma in situ (CIS), and finally to invasive and then metastatic LUSC^3^. In fact, the presence of high-grade persistent or progressive dysplasia (moderate or severe) is a marker of increased LC risk both at the lesion site (where they are the presumed precursors of squamous cell lung cancer) and elsewhere in the lung, although many dysplastic lesions do have varied outcomes^4,5^. Currently, however, we lack effective tools to identify PMLs at highest risk of progression to invasive carcinoma^6^. The development of markers of disease progression would identify patients at high-risk, suggest novel lung cancer chemoprevention agents, and provide molecular biomarkers for monitoring outcome in lung cancer prevention trials.

We hypothesized that molecular characterization of bronchial endobronchial biopsies containing a mixture of epithelial and immune cells would allow us to identify transcriptomic alterations associated with high-grade histology and premalignant lesion progression. In this study, we used mRNA sequencing (mRNA-seq) to profile endobronchial biopsies and brushings obtained through serial bronchoscopies from high-risk smokers undergoing lung cancer screening by autofluorescence bronchoscopy and chest computed tomography (CT). Using the bronchial biopsies, we identified four molecular subtypes associated with clinical phenotypes and biological processes. One subtype (Proliferative subtype) is enriched with bronchial dysplasia, high basal cell and low ciliated cell signals, and expression of proliferation-associated pathways. Genes involved in interferon signaling and T-cell-mediated immunity were down regulated among progressive/persistent lesions within the Proliferative subtype compared with regressive lesions and these pathways correlated with decreases in both innate and adaptive immune cell types. Molecular classification of biopsies into a high-grade/progressive disease group may be used to stratify patients into prevention trials and to monitor efficacy of the treatment. The results also suggest that personalized lung cancer chemoprevention, targeting specific cancer-related pathways or the immune system may have potential therapeutic benefits.

## Results

### Subject population

In this study, we used mRNA-seq to profile endobronchial biopsies and brushings obtained through serial bronchoscopy of high-risk smokers undergoing lung cancer screening by autofluorescence bronchoscopy and chest CT at the Roswell Park Comprehensive Cancer Center (Roswell) in Buffalo, NY. The Discovery Cohort samples were obtained from the Roswell subjects between 2010 and 2012 (DC; *n* = 29 patients, *n* = 191 biopsies, *n* = 91 brushes), and the Validation Cohort samples were obtained between 2012 and 2015 (VC; *n* = 20 patients, *n* = 111 biopsies, and 49 brushes). The subjects are predominantly older smokers, many of which have a prior history of lung cancer, chronic obstructive pulmonary disease (COPD), and occupational exposures that confer a high-risk of developing lung cancer. Clinical characteristics reported at the baseline visit such as sex, age, smoking status (ever or never), pack-years, prior history of lung cancer, COPD status, and occupational exposures were not significantly different between the two cohorts (Table 1). After sample filtering based on several quality metrics, the DC had 190 biopsies and 89 brushes, whereas the VC had 105 biopsies and 48 brushes. Ninety-four percent of subjects had at least one lung anatomic location sampled two or more times via endobronchial biopsy. The DC and VC contained 37.9% and 35.2% biopsies with a histological grade of dysplasia or higher and 23.1% and 19.0% had progressive/persistent dysplasia, respectively (Table 2). We used a previously described smoking-associated signature^7^ to predict the smoking status of each sample, as smoking status was only available at baseline. The predicted smoking status was consistent across all procedures for 63% and 70% of the DC and VC subjects, respectively (Supplementary Table 1). In terms of RNA sequencing quality, the DC had significantly greater total reads, percent uniquely mapping reads, and median transcript integrity number scores^8^ among the biopsies than the VC, but these differences between cohorts were not reflected in the brushes (Supplementary Table 2).

**Table 1:** Subject demographic and clinical annotation in the discovery and validation cohorts

Variable	Discovery cohort (*n* = 30)	Validation cohort (*n* = 20)	*P* value
Average # biopsies/subject	6.6 (5.7)	5.25 (2.9)	0.3
Average # bronchoscopies/subject	3.1 (1.6)	2.5 (0.7)	0.08
Average time between bronchoscopies (days)	348.6 (197.5)	366.8 (208.3)	0.69
Male	15/30 (50)	12/20 (60)	0.81
White	27/30 (90)	17/20 (85)	1
Age (at baseline clinical visit)	58.8 (7.6)	58.7 (8.3)	0.97
Ever smoker (at baseline clinical visit)	29/30 (96.7)	19/20 (95)	1
Pack-years (at baseline clinical visit)	49.8 (22.1)	41.3 (20.7)	0.17
Prior history of lung cancer	21/30 (70)	12/20 (60)	0.82
Prior history of LUSC	5/30 (16.7)	5/20 (25)	0.73
COPD (FEV1/FVC ≤ 0.7, at baseline clinical visit)	17/27 (63.0)	8/18 (44.4)	0.61
GOLD 1 (FEV1%> 80)	2/27 (7.4)	2/18 (11.1)	1
GOLD 2 (FEV1% < 80 and > 50)	12/27 (44.4)	5/18 (27.8)	0.56
GOLD 3 (FEV1% < 50 and > 30)	3/27 (11.1)	1/18 (5.6)	1
Occupational asbestos	13/30 (43.3)	9/20 (45)	1
Occupational high-risk job	14/30 (46.7)	12/20 (60)	0.62

Statistical tests between the discovery and validation cohorts were performed using two-sided Fisher’s exact tests for categorical variables and two-sided Student’s *t*-tests for continuous variables. Percentages are reported for categorical variables and mean and standard deviations are reported for continuous variables. Source data are provided as a Source Data file

**Table 2:** Sample clinical annotation in the discovery and validation cohorts

Variable	Discovery cohort		Validation cohort		*P* value	
Sample type	Biopsies	Brushes	Biopsies	Brushes	Biopsies	Brushes
Histology					0.05	0.42
Normal	38/190 (20)	6/89 (6.7)	23/105 (21.9)	0/48 (0)		
Hyperplasia	30/190 (15.8)	11/89 (12.4)	31/105 (29.5)	9/48 (18.8)		
Metaplasia	46/190 (24.2)	15/89 (16.9)	14/105 (13.3)	9/48 (18.8)		
Mild dysplasia	21/190 (11.1)	9/89 (10.1)	13/105 (12.4)	6/48 (12.5)		
Moderate dysplasia	38/190 (20)	30/89 (33.7)	20/105 (19.0)	18/48 (37.5)		
Severe dysplasia	12/190(6.3)	17/89 (19.1)	4/105 (3.8)	6/48 (12.5)		
Carcinoma in situ	1/190 (0.5)	0/89 (0)	0/105 (0)	0/48 (0)		
Tumor	0/190 (0)	1/89 (1.1)	0/105 (0)	0/48 (0)		
Unknown histology	4/190 (2.1)	0/89 (0)	0/105 (0)	0/48 (0)		
Current smoker (genomic prediction)	119/190 (62.6)	44/89 (49.4)	38/105 (36.2)	20/48 (41.7)	1.80E-05	0.47
Progression status					0.39	
Normal/stable	47/190 (24.7)		35/105 (33.3)			
Progressive/persistent	44/190(23.2)		20/105 (19.0)			
Regressive	30/190 (15.8)		18/105 (17.1)			
Unknown	69/190 (36.3)		32/105 (30.5)			

Statistical tests between the discovery and validation cohorts within either the biopsies or brushes were performed using two-sided Fisher’s exact tests and percentages are reported. Source data are provided as a Source Data file

### LUSC PMLs divide into distinct molecular subtypes

**Fig. 1:**
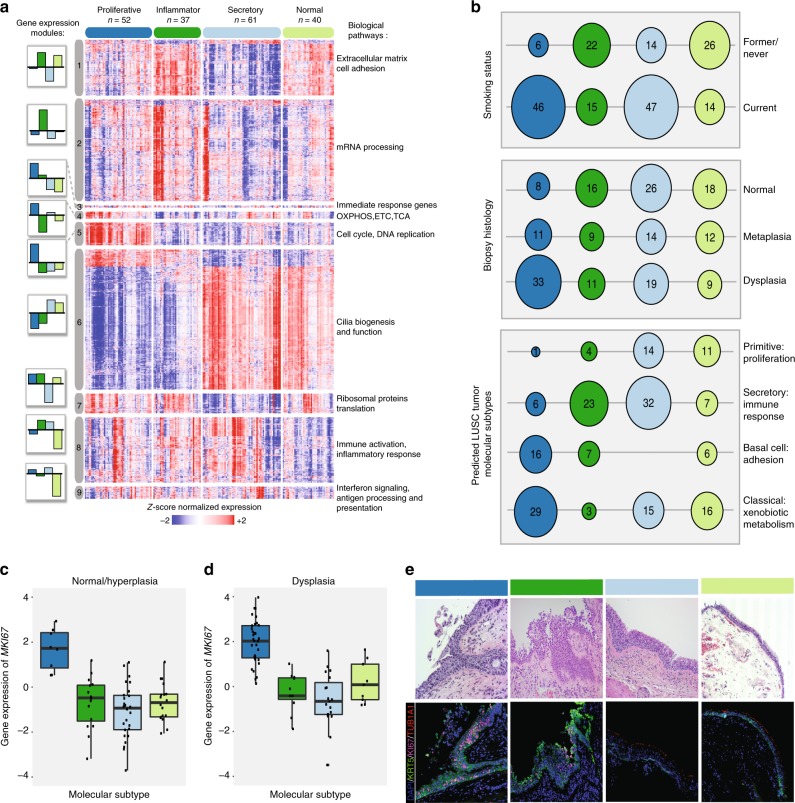
Endobronchial biopsies divide into four distinct molecular subtypes that correlate with clinical and molecular phenotypes. **a** Genes (*n* = 3936) organized into nine gene co-expression modules were used to discover four molecular subtypes (Proliferative, Inflammatory, Secretory, and Normal-like) across the 190 DC biopsies using consensus clustering. The heatmap shows semi-supervised hierarchal clustering of z*-*score-normalized gene expression across the 3936 genes and 190 DC biopsies. The top color bar represents the four molecular subtypes: Proliferative (*n* = 52 samples), Inflammatory (*n* = 37 samples), Secretory (*n* = 61 samples), and Normal-like (*n* = 40 samples). To the left of the heatmap, barplots for each module show the mean module GSVA score for each subtype. To the right of the heatmap, a summary of enriched biological pathways is listed for each module. **b** Bubbleplots showing significant associations (*p* < 0.01, two-sided Fisher’s exact test) between the molecular subtypes and genomic smoking status, biopsy histological grade, and the predicted LUSC tumor molecular subtypes. The columns represent the four molecular subtypes (Proliferative, Inflammatory, Secretory, and Normal-like) and the diameter of the circle is proportional to the number of samples within each subtype that have the row phenotype. **c** Boxplot of *MKI67* expression values in biopsies with normal or hyperplasia histology (*n* = 8, 16, 26, 18 in Proliferative, Inflammatory, Secretory, and Normal-like subtypes, respectively). The *MKI67* expression levels of the Proliferative subtype are significantly greater than non-Proliferative subtype samples (FDR = 3.4e-10, linear model). **d** Boxplot of expression values of MKI67 in biopsies with dysplastic histology (*n* = 33, 11, 19, 9 in Proliferative, Inflammatory, Secretory, and Normal-like subtypes, respectively). The *MKI67* expression levels of the Proliferative subtype are significantly greater than non-Proliferative subtype samples (FDR = 3.1e-8). **e** Immunofluorescent staining demonstrating the increased MKI67 and KRT5 staining and reduced TUB1A1 staining in the Proliferative subtype. The representative samples shown for the Proliferative and Inflammatory subtypes have dysplasia histology, whereas the samples shown for the Secretory and Normal-like subtypes have normal histology (Magnification ×  200). In the boxplots, the upper and lower hinges correspond to the first and third quartile, center line represents the median, and whiskers extend from the hinge to the largest or smallest value at most 1.5 times the distance between the quartiles. Source data are provided as a Source Data file

In order to identify gene expression differences associated with LUSC PML histological severity using the endobronchial biopsies, we used a discovery-based approach to identify de novo molecular subtypes based on distinct patterns of gene co-expression (gene modules). The approach was chosen given that there is histological heterogeneity within biopsies and that pathological analyses were conducted using biopsies adjacent to biopsies profiled via mRNA-Seq. First, we sought to select a set of gene modules that are present across different LUSC–related data sets. Using weighted gene co-expression network analysis^9^ (WGCNA), gene modules were derived in the DC biopsies (*n* = 190 samples, *n* = 16653 genes, *n* = 15 gene modules), the DC brushes (*n* = 89 samples, *n* = 16058 genes, *n* = 47 gene modules), TCGA LUSC tumors^10^ (*n* = 471 samples, *n* = 17887 genes, *n* = 55 gene modules), and tracheobronchial samples from mice treated with *n*-nitrosotris-(2-choroethyl)urea (NTCU) (*n* = 25 samples, *n* = 14897 genes, *n* = 40 gene modules). DC biopsy gene modules that were highly correlated (absolute Pearson correlation coefficient *r* > 0.85) to at least one other non-DC biopsy module within each of the four data sets were selected. Genes in the selected modules were filtered by requiring that each gene was also present in at least one of the correlated non-DC biopsy modules, resulting in a set of nine gene modules that consisted of 3936 genes in total (Supplementary Table 3). These gene modules identified four molecular subtypes within the DC biopsies via consensus clustering: Proliferative (dark blue, *n* = 52 samples, 27.4%), Inflammatory (dark green, *n* = 37 samples, 19.5%), Secretory (light blue, *n* = 61 samples, 32.1%), and Normal-like (light green, *n* = 40 samples, 21.1%) (Fig. 1a, Table 3).

**Table 3:** Molecular subtype characteristics in the discovery cohort

Proliferative	
Up-regulated modules	4, 5, 7
Down-regulated modules	6
Clinical characteristics	Current smoking (86%), dysplastic biopsies (63%)
Biological characteristics	LUSC subytpes—classical and basal; *TUB1A1*, *SCGB1A1* down regulated; *KRT5*, M*KI67* up regulated
Pathways	Cell cycle: *BUB1B/1/3, CHEK1/2, CDK1/2/4/6, E2F1/3/2/4, MCM4/3/5/6/7, TP53, RB1*
	DNA repair: *TP53, PARP1, RAD51, BRCA2, FANCA/D2/G/E/M/C, XRCC5/6, ERCC6*
	Oxidative phosphorylation and electron transport chain: ATP synthases, NADH-ubiquinone oxidoreductases, cytochrome C oxidases
Transcription factors	*E2F*
Inflammatory	
Up-regulated modules	1, 2, 7, 8
Down-regulated modules	4, 5, 6
Clinical characteristics	Former smoking (59%), non-dysplastic biopsies (68%)
Biological characteristics	LUSC subytpes—secretory; *TUB1A1, MUC5AC* down regulated
Pathways	Extracellular matrix, focal adhesion, and integrin pathways: collagen, integrin, and laminin genes
	Cytokine/chemokine: *CCL2/14/19/21/28, CXCL12/14/5, CCR1/2/3/4/5, IL1B, IL11RA, IL17RB, IL1R1, IL3RA, EGF, IL15, CX3CR1, TGFB1/B2/B3, KIT*
	Downregulation of oxidative phosphorylation, respiratory elecron transport, cell cycle
Transcription factors	*SRF*
Secretory	
Up-regulated modules	6, 8
Down-regulated modules	1, 2, 5, 7
Clinical characteristics	Current smoking (63%), non-dysplastic biopsies (66%)
Biological characteristics	LUSC subytpes—secretory; *CD45, MUC5AC, TUB1A1* up regulated; M*KI67, KRT5* down regulated
Pathways	Down regulation of extracellular matrix, focal adhesion, integrin pathways
Transcription factors	Down regulation of *E2F*
Normal-like	
Up-regulated modules	1, 6
Down-regulated modules	8, 9
Clinical characteristics	Former smoking (65%), non-dysplastic biopsies (75%)
Biological characteristics	*CD45, MUC5AC, MKI67* down regulated; *SCGB1A1, KRT5, TUB1A1* up regulated
Pathways	Core extracellular matrix genes: collagen and laminin genes, *WISP1/2*
	Down regulation of innate and adaptive immunity: HLA genes, *IRF1/4/7/8, TLR2/4/6/8/10, IKBKB*
Transcription factors	Down regulation of *PEA3, IRF, NFKB*

For each molecular subtype, significant associations are reported  between the molecular subtype and gene module GSVA scores, clinical characteristics, canonical cell type epithelial and white blood cell gene markers, biological pathways, and transcription factors. The modules are designated as up regulated or down regulated in each molecular subtype based on the direction of gene expression of the majority of genes in each module. Source data are provided as a Source Data file

In order to characterize each molecular subtype, we first focused on identifying biological pathways enriched in each module, as the pattern of gene module expression defines the PML subtypes. Each gene module was found to be associated with distinct epithelial and immune biological processes (Fig. 1a, Supplementary Tables 3 and 4, Data 1). The Proliferative subtype is specifically characterized by increased expression of genes involved in energy metabolism and cell cycle pathways (Modules 4 and 5). The Secretory and Normal-like subtypes both have increased expression of genes in cilium-associated pathways (Module 6), however, the Normal-like subtype specifically has decreased expression of genes involved in inflammation, regulation of lymphocytes and leukocytes, and antigen processing and presentation pathways (Modules 8 and 9). The Secretory subtype exhibits decreased expression of genes involved in protein translation (Module 7), whereas RNA processing genes (Module 2) are expressed more highly in the Inflammatory subtype.

We further characterized our molecular subtypes by their associations with clinical phenotypes and established LUSC tumor molecular subtypes^11,12^. Sample genomic smoking status, the subject from whom the sample was derived, and sample histology demonstrated significant associations with molecular subtype (*p* < 0.01, two-sided Fisher’s exact test, Fig. 1b, Supplementary Tables 5 and 6, Supplementary Figs. 1, 2, and 3). The Proliferative and Secretory subtypes are enriched for current smokers and the Proliferative subtype is enriched for bronchial dysplasia (Fig. 1b). The Proliferative subtype has high expression of genes involved in cell cycle processes including the proliferation marker *MKI67*, which is significantly up regulated among samples in this subtype compared with samples in other subtypes (false discovery rate; FDR = 1.0e-30, linear model). The gene remained significantly up regulated in Proliferative normal/hyperplasia samples (FDR = 3.4e-10, linear model) and dysplasia samples (FDR = 3.1e-8, linear model), and these observations are supported by an increase in protein expression in representative samples (*p* = 0.02, linear model) (Fig. 1c–e, Supplementary Fig. 4, and Supplementary Table 7). The Proliferative subtype samples also had high concordance with the LUSC–Classical subtype (Fig. 1b). In the TCGA LUSC tumors, the LUSC–Classical subtype was associated with alterations and over expression of *KEAP1* and *NFE2L2* as well as amplification of 3q26 with over expression of *SOX2*, *TP63,* and *PIK3CA*^11^. Similarly, our Proliferative PMLs have increased expression of *KEAP1*, *NFE2L2*, *TP63*, and *PIK3CA* (FDR = 1.4e-6, 4.5e-12, 1.4e-9, and 0.03, respectively, linear model) (Supplementary Fig. 5A). Furthermore, the LUSC–Classical subtype was associated with increased expression of genes involved in energy metabolism, and our Proliferative subtype is in part defined by high expression of Module 4, that is enriched with genes involved in oxidative phosphorylation and the electron transport chain. In contrast, the Inflammatory and Secretory PML subtypes demonstrate enrichment for the LUSC–Secretory subtype. The LUSC–Secretory subtype was associated with the immune response, and the Inflammatory, and Secretory PMLs have the highest expression of Module 8 that is enriched for genes in these same pathways.

Finally, we wanted to examine the extent to which our PML molecular subtypes were driven by differences in epithelial and immune cell type composition by assessing expression of a number of canonical cell type markers. The Inflammatory and Secretory subtypes have higher levels of expression of the white blood cell marker *PTPRC* (*CD45*) consistent with enrichment of the LUSC–Secretory subtype (Supplementary Fig. 5B, FDR = 0.12 and 0.01, respectively, linear model). Consistent with the pathways enriched in Module 6, the ciliated cell marker *TUB1A1* expression is decreased in the Inflammatory and Proliferative subtypes (FDR = 1.1e-4 and 3.5e-19, respectively, linear model), and this is also shown by a decrease in acetylated α-tubulin staining in representative histological samples (Fig. 1e, Supplementary Fig. 4, and Supplementary Table 7). The Proliferative subtype has the highest expression (FDR = 2.4e-15, linear model) of basal cell marker (*KRT5*), indicating enrichment of lesions with high-grade histology that tightly correlates with protein expression in representative histology samples (*p* = 0.01, linear model) (Fig. 1e, Supplementary Fig. 4, and 5B, Supplementary Table 7). In addition, gene expression of *MUC5AC*, a marker of goblet secretory cells, is increased in subtypes enriched for current smokers (Proliferative and Secretory) but is the most significantly increased in the Secretory subtype (FDR = 3.4e-5, linear model). In contrast, gene expression of *SCGB1A1*, a marker of club cells, is the lowest in the Proliferative subtype (FDR = 6.1e-5, linear model). The Normal-like subtype is supported by expression of all epithelial cell types and has the lowest expression of *CD45* (FDR = 7.6e-4, linear model, Supplementary Figs. 5B–D). The expression levels of these marker genes agree with cell type deconvolution methods to examine epithelial and immune cell content (Supplementary Figs. 5C–D). The summation of these characterizations highlights epithelial and immune cell associated pathways that are modulated by smoking and PML histology and identifies the Proliferative subtype as a subset of high-grade PMLs that express proliferative and cell cycle-related pathways.

### Molecular subtypes are replicated in the VC

**Fig. 2:**
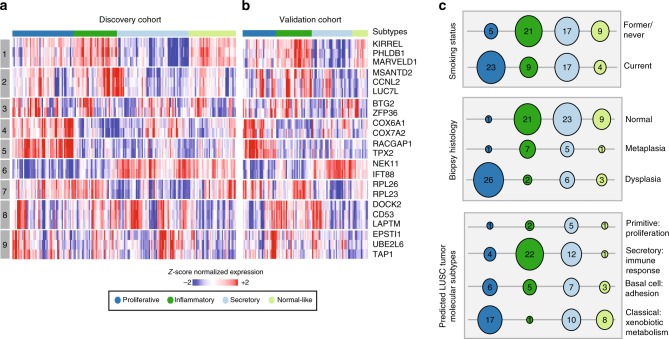
Phenotypic associations with the molecular subtypes are confirmed in an independent sample set. **a** The 190 DC biopsies and the 3936 genes were used to build a 22-gene nearest centroid molecular subtype classifier. The heatmap shows semi-supervised hierarchal clustering of z-score normalized gene expression across the 22 classifier genes and 190 DC biopsies training samples. **b** The 22-gene nearest centroid molecular subtype classifier was used to predict the molecular subtypes of the 105 VC biopsies. The heatmap shows semi-supervised hierarchal clustering of z-score normalized gene expression across 22 genes and 105 VC. The rows of the heatmap give the gene name and module membership, and the column color bar shows molecular subtype membership. **c** Bubbleplots showing significant associations (*p* < 0.01 by two-sided Fisher’s exact test) between the VC molecular subtypes and smoking status, biopsy histological grade, and the predicted LUSC tumor molecular subtypes. The columns represent the four molecular subtypes (Proliferative, Inflammatory, Secretory, and Normal-like) and the radius of the circle is proportional to the number of samples within each subtype that have the row phenotype. Source data are provided as a Source Data file

Next, we wanted to determine whether the heterogeneity captured in the DC biopsy-derived molecular subtypes was reproducible in the VC. We developed a 22-gene nearest centroid molecular subtype predictor by selecting genes highly correlated with each of the gene module eigengenes. The predictor has 84.7% accuracy across DC biopsies (training set, Fig. 2a and Supplementary Fig. 6) with the following misclassification rates per subtype 5/52 (9.6%) in Proliferative, 7/37 (18.9%) in Inflammatory, 9/61 (14.8%) in Secretory, and 8/40 (20%) in Normal-like. The 22-gene classifier was used to predict the molecular subtype of the 105 VC biopsies (Fig. 2b). The VC subtype predictions were evaluated by examining the concordance of Gene Set Variation Analysis (GSVA)^13^ scores for each of the 9 modules (using the full set of genes for each module) between the predicted VC subtypes compared with the DC subtypes. The average behavior of PC1 across the subtypes was highly similar (Supplementary Fig. 7) with few exceptions (namely, Module 3 that had the fewest genes). In addition, we compared the VC subtype predictions from the 22-gene classifier to subtypes derived in the VC biopsies using the same methodology used to derive the DC subtypes and found significant concordance (*p* = 1.0e-7, two-sided Fisher’s exact test, with the Proliferative subtype having the greatest concordance between predictions, Supplementary Fig. 6).

The statistical associations between the VC subtypes (via the 22-gene classifier) and clinical and molecular phenotypes across the VC biopsies are analogous to those observed across the DC biopsies (Fig. 2c, Supplementary Tables 5 and 6, Supplementary Figs. 1 and 3). In brief, the Proliferative subtype is enriched for current smokers, biopsies with bronchial dysplasia, and the LUSC–Classical tumor subtype (Fig. 2c, Supplementary Table 5). Epithelial and white blood cell marker gene expression across the VC biopsies reveals higher levels of the white blood cell marker *PTPRC* (*CD45* expression) in the Inflammatory subtype (FDR = 0.002, linear model) consistent with enrichment of the LUSC–Secretory subtype (Supplementary Fig. 5F). The Inflammatory and Proliferative subtypes have reduced ciliated cell marker expression (*FOXJ1*) consistent with Module 6 (*FOXJ1* FDR = 0.0005 and FDR = 2.62e-6 and Module 6 FDR = 5.73e-6 and FDR = 4.34e-10, respectively, linear models). The Proliferative subtype has the highest expression of basal cell marker *KRT5* (FDR = 1.67e-7, linear model), proliferation marker *MKI67* (FDR = 3.03e-10, linear model), and cell cycle-associated Module 5 (FDR = 1.23e-18, linear model) indicating enrichment of lesions expressing characteristics associated with high-grade histology. Gene expression of *SCGB1A1*, a marker of club cells, is the lowest in the Proliferative subtype (FDR = 1.8e-4, linear model). All epithelial cell type markers are expressed in the Normal-like subtype, and *CD45* expression is decreased (FDR = 0.14, linear model, Supplementary Figs. 5F–G). Gene expression of *MUC5AC*, a marker of goblet epithelial cells, was increased in current smokers and most significantly in the Secretory subtype in the DC biopsies; however, in the VC biopsies this trend is not preserved as current smokers are not enriched in the Secretory subtype. The expression levels of these marker genes agree with other deconvolution methods to examine epithelial and immune cell content (Supplementary Figs. 5E–H).

### Airway brushes reflect biopsy Proliferative subtype

**Fig. 3:**
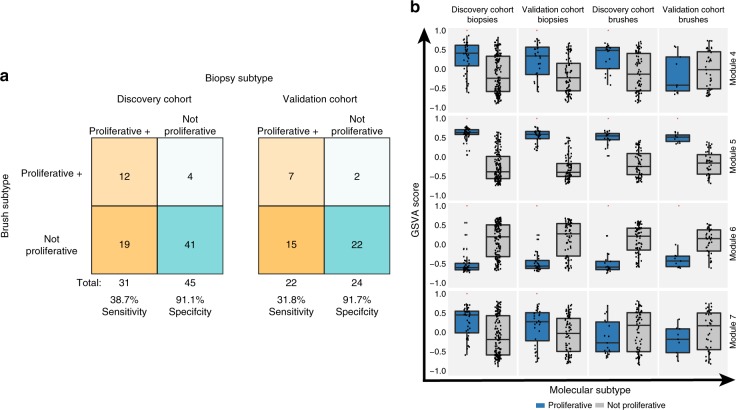
Normal-appearing bronchial brushes predict the presence of proliferative lesions. **a** The DC (left) and VC (right) cohorts, showing the number of brushes (*y* axis) classified as proliferative orange) that have at least one biopsy (*y* axis) classified as proliferative at the time the brush was sampled. Brushes/biopsies classified as not proliferative are turquoise. **b** Boxplots of GSVA scores for modules 4, 5, 6, and 7 (*y* axis) across all brushes (*n* = 86 in DC and *n* = 48 in VC) and biopsies (*n* = 190 in DC and *n* = 105 in VC) from each cohort classified as Proliferative or not Proliferative (*x* axis). The red asterisk indicates significant differences between the Proliferative subtype versus all other samples (FDR < 0.05, linear model). In the boxplots, the upper and lower hinges correspond to the first and third quartile, center line represents the median, and whiskers extend from the hinge to the largest or smallest value at most 1.5 times the distance between the quartiles. Source data are provided as a Source Data file

Previously, we have shown that bronchial brushes from normal-appearing areas of the mainstem bronchus could predict the presence of PMLs^14^; however, that study lacked biopsies and brushes from the same subjects. Above, in both the DC and the VC biopsies, the Proliferative subtype, represents a distinct subtype of PMLs enriched for dysplastic histology expressing metabolic and proliferative pathways. Biopsies classified as the Proliferative subtype may represent a group of PMLs that need close monitoring and intervention. As a result, we sought to explore whether or not we could predict the presence of Proliferative subtype biopsies using the brushes. The Proliferative subtype is defined by the behavior of Modules 4, 5, 6, and 7 (Table 3), and therefore, we used the subset of 8 genes (from the 22-gene predictor) that correspond to these modules to predict presence/absence of the Proliferative subtype across the DC and VC biopsies and brushes. A prediction of the Proliferative subtype in a brush is specific (91% and 92% in the DC and VC biopsies, respectively), but not sensitive (39% and 32% DC and VC biopsies, respectively) at indicating the presence of at least one Proliferative PML detected at the same time point (Fig. 3a). In order to understand the classifier’s performance in predicting the Proliferative subtype in brushes, we examined GSVA scores for Modules 4, 5, 6, and 7 that define the Proliferative subtype in the DC and VC brushes (Fig. 3b). In the DC and VC brushes, the GSVA scores were significantly different (FDR < 0.05) in the Proliferative subtype versus all other samples only for Modules 5 and 6, and thus these likely contribute the most heavily to Proliferative subtype classification in the brushes. Module 5 contains genes associated with cell cycle and proliferation, whereas Module 6 contains genes associated with cilium assembly and organization. Upregulation of Module 5 and downregulation of Module 6 in the brushes specifically predicts the presence of a Proliferative subtype PML; however, the absence of these signals in the airway field of injury does not preclude the development of a Proliferative subtype PML.

### Immune genes associate with PML progression

**Fig. 4:**
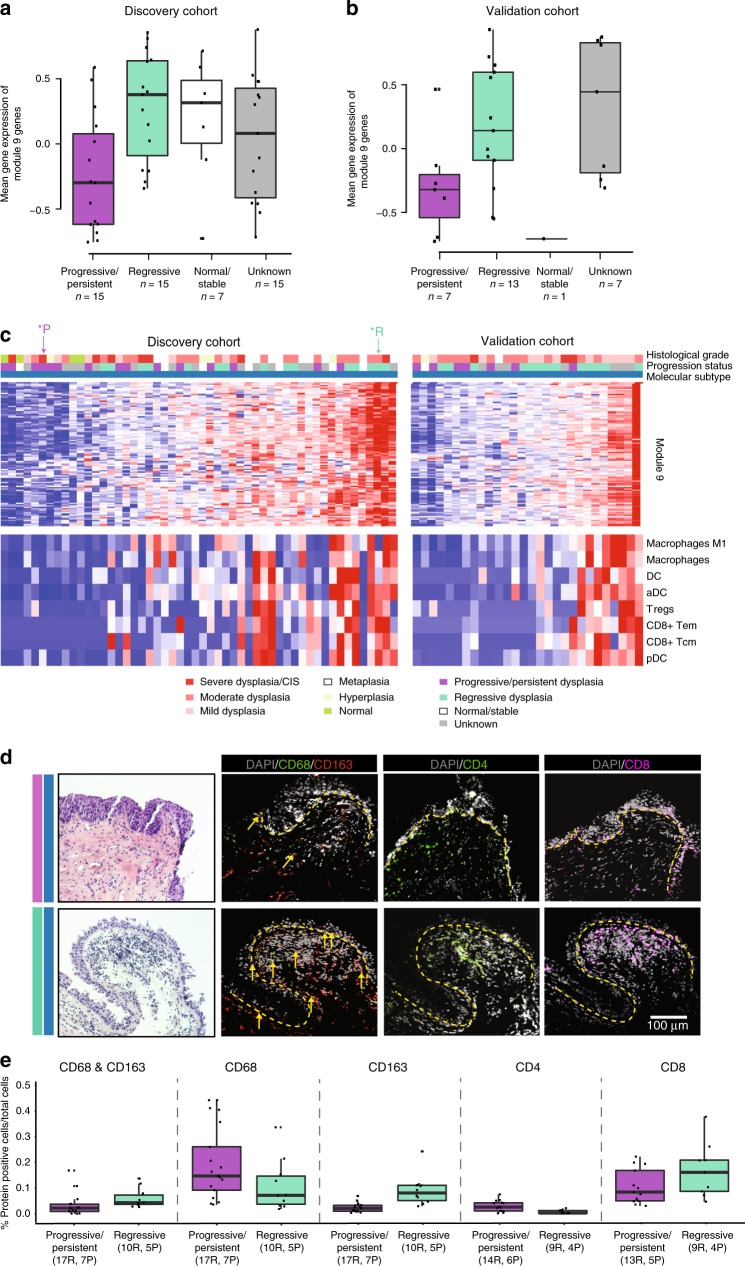
Immune alterations are associated with lesion outcome in the Proliferative subtype. Boxplots of Module 9 GSVA scores across DC **a** and VC biopsies **b** within the Proliferative subtype. There is a significant difference between the progressive/persistent versus regressive biopsies (*p* = 0.002 (DC) and *p* = 0.03 (VC), linear models). **c** Top: heatmap of z-score-normalized gene expression across the 112 genes in Module 9 in the DC biopsies (left) and the VC biopsies (right). Each heatmap is supervised by Module 9 GSVA scores. Top color bars indicate the histological grade of the biopsies and their progression status. Bottom: heatmap of xCell results indicating the relative abundance of immune cell types across the DC biopsies (left) and the VC biopsies (right). Immune cell types displayed are significantly associated with lesion progression/persistence (FDR < 0.05 in both the DC and VC, linear model). **d** Representative histology where the dashed yellow line denotes the separation of epithelium and stromal compartments. Top panels: a progressive severe dysplasia has reduced presence of immune cells demonstrated by the marked reduction in expression of M2 macrophages (CD68/163 staining, double-positive cells indicated by the yellow arrows) and CD8 T cells (sample corresponds to *P in **c**). Bottom panels: a regressive moderate dysplasia has increased presence of immune cells including M2 macrophages (CD68/163 staining double-positive cells indicated by the yellow arrows) and CD8 T cells (samples correspond to *R in **c**). **e** Boxplots of the percentages of CD68 and CD163, CD68, CD163, CD4, and CD8 positively stained cells between progressive/persistent and regressive biopsies (*p* < 0.001, linear model, for all comparisons). The *x* axis labels indicate the number of regions (R) enumerated across (P) subjects for each stain and outcome group depicted in the boxplot. Biopsies were included in the analysis if their clinical outcome was concordant with the Module 9 score. In the boxplots, the upper and lower hinges correspond to the first and third quartile, center line represents the median, and whiskers extend from the hinge to the largest or smallest value at most 1.5 times the distance between the quartiles. Source data are provided as a Source Data file

Previous studies of bronchial PMLs suggest that high-grade lesions (which occur more frequently in current smokers) are more likely to progress to invasive carcinoma^5^. Therefore, we sought to identify molecular alterations associated with subsequent PML progression/persistence (*n* = 15) versus regression (*n* = 15) among the Proliferative subtype DC biopsies, as these may be clinically relevant to identifying appropriate interception strategies. Using GSVA scores calculated across all the DC biopsies for each of the nine modules, we calculated which scores were statistically different between progressive/persistent versus regressive disease in the samples belonging to the Proliferative subtype (Supplementary Table 8). We found that the DC biopsy GSVA module scores for Module 9 were significantly higher among regressive Proliferative PMLs (*p* = 0.002, linear model, Fig. 4a) compared with progressive/persistent Proliferative PMLs. The association between low Module 9 scores and progression/persistence is replicated in the VC biopsies (*n* = 7 progressive/persistent and *n* = 13 regressive biopsies; *p* = 0.03, linear model, Fig. 4b). The ability of the Module 9 GSVA scores to discriminate between regressive versus progressing/persistent biopsies as measured by the area under the receiver operating characteristic was 0.809 and 0.802 in the DC and VC biopsies, respectively.

The genes in Module 9 include a number of genes that encode for proteins involved in interferon signaling as well as antigen processing and presentation (*SP100, CIITA, CXCL10, SOCS1, GBP1, GBP4, B2M, TAP1, TAPBP, TRIM14, TRIM21, TRIM22, STAT1, PML, OAS2, OAS3, MX1, ADAR, ISG15, IFI35, IFIT3, IFI27, PSMB8, PSMB9, BST2, IRF1, IRF9, CD74, PSME1, PSME2, HLA-DQA1/DPA1/ DPB1/DRA/ DQB2/DRB1/ DQB1/DMA/DMB/DOA, HLA-A/B/C/E/F*) and include the inhibitory receptor *LAG3*. As a result, we wanted to evaluate whether or not the presence or absence of innate or adaptive immune cells were associated with Module 9 expression within the Proliferative subtype. In an effort to deconvolute the potential presence of immune cell types, we generated GSVA scores using previously described immune cell signatures^15^ and scores for 64 different cell types using the xCell algorithm^16^, separately for both the DC and VC biopsies. We identified significant (FDR < 0.05, linear model) associations between the cell type scores and Module 9 that were in common between the DC and VC biopsies (Supplementary Fig. 8) and identified eight cell types (via xCell): dendritic cells, activated dendritic cells, plasmacytoid dendritic cells, macrophages, M1 macrophages as well as CD8+ effector memory T cells, CD8+ central memory T cells, and T regulatory cells (Fig. 4c). Taken together, the progressive/persistent biopsies in the Proliferative subtype have down-regulated expression of Module 9 compared with regressive biopsies that correlates with reduced signals from both innate and adaptive immune cell populations.

### Immune cell populations are altered in PML progression

In order to confirm the relationship between the immune cell types associated with Module 9 and histologic progression/persistence of PMLs in the Proliferative subtype, immunofluorescent staining of macrophages/monocytes (*n* = 52 regions enumerated from *n* = 16 subjects), CD4 (*n* = 50 regions enumerated from *n* = 17 subjects), and CD8 T cells (*n* = 47 regions enumerated from *n* = 16 subjects) was performed (Supplementary Table 7). The results were analyzed across all subjects assayed within the Proliferative subtype and across the subset of subjects where the lesion outcome (progression/persistence versus regression) was concordant with the Module 9 GSVA score (denoted as concordant set). Staining of CD68, a pan macrophage (and tumor-associated macrophage) marker, suggestive of M1 type macrophages, was increased in progressive/persistent lesions (*p* ≪ 0.001 in the concordant set). In contrast, staining of CD163 in combination with CD68, thought to be suggestive of M2 type macrophages, were decreased among the progressive/persistent lesions in the Proliferative subtype (*p* ≪ 0.001 using all subjects and *p* = 0.0007 in the concordant set, respectively, linear model) (Fig. 4d–e). In addition, CD4 T cells were increased (*p* ≪ 0.001 in the concordant set, linear model and CD8 T cells were decreased (*p* ≪ 0.001 in the concordant set, linear model) in PMLs that progress/persist. Interestingly, among progressive/persistent lesions, the CD8 T cells had a distinct localization pattern (*p* = 0.07 in the concordant set, linear model), where CD8 T cells both lined and were embedded within the epithelium in areas where dysplasia is present (Fig. 4d). The immunofluorescence results did not reach significance, with the exception of CD163, when just the lesion outcome was used without regard to the Module 9 score.

## Discussion

LUSC is the second most common form of lung cancer. LUSC arises in the epithelial layer of the bronchial airways, and is often preceded by the development of lung squamous PMLs. The presence of dysplastic persistent and or progressive PMLs is a marker of increased risk for LUSC^5^. Currently, however, we lack effective tools to identify PMLs at highest risk of progression to invasive carcinoma^6^. The development of markers predictive of disease progression will be important in identifying patients at highest risk for LUSC development and in identifying biological pathways exploitable for LUSC chemoprevention. Towards this goal, we profile via mRNA-Seq bronchial brushes and endobronchial biopsies obtained from subjects undergoing longitudinal lung cancer screening by chest CT and autofluorescence bronchoscopy. We identify four transcriptionally distinct groups of biopsies, one of these we label Proliferative and find it to be associated with bronchial dysplasia. Patients with Proliferative PMLs can also be identified via gene expression measured from cells in the non-involved large airway epithelium. We further find that persistent/progressive Proliferative PMLs are characterized by decreased expression of genes involved in interferon signaling and antigen processing/presentation pathways. Consistent with these gene expression findings we find that progressive/persistent Proliferative PMLs are depleted for CD68^+^/CD163^+^ macrophages and CD8 T cells by immunofluorescence. Collectively, these data suggest both the potential to identify a subset of patients with progressive/persistent LUSC PMLs, who are at risk for developing invasive lung cancer, on the basis of airway gene expression; as well as the potential to decrease the risk for progression in these patients by augmenting the immune response associated with regression.

Previous studies indicate a range of genomic alterations associated with bronchial dysplasia. Increased expression of EGFR and Ki67 staining of epithelial cells is associated with increasing histologic severity and subsequent histologic progression^5,17^. Altered protein levels of TP53, CCND1, CCNE1, BAX, and BCL2 have been associated with CIS or lung cancer occurrence independent of histological grade^18^. Telomere shortening and maintenance^19^ and loss of heterozygosity in regions frequently deleted in lung cancer (3p, 5q, 9p, 13q, 17p) has been observed in early hyperplasia/metaplasia lesions^20–22^ and found to increase in frequency and size in higher-grade dysplasia. Genomic gains in loci containing *SOX2, TP63, EGFR, MYC, CEP3*, and *CEP5* are also associated with progression of high-grade dysplasia^23^. Despite the numerous genomic alterations associated with PML histological grade and progression, we lack a comprehensive PML molecular classification system to complement pathologic examination. We pursued an unsupervised class discovery approach that led to the identification of four distinct molecular PML subtypes (Proliferative, Inflammatory, Secretory, and Normal-like). The transcriptional patterns differentiating the PML subtypes are robust and a 22-gene panel identified in the DC can be used to distinguish between the molecular subtypes in an independent VC. Interestingly, whereas prior lung cancer history may influence airway gene expression and about two-thirds of the subjects have a prior history of lung cancer, we do not detect a significant association between lung cancer history and molecular subtype, and there is a similar diversity of molecular subtypes between biopsies collected from subjects with and without a lung cancer history. This observation is supported by prior work describing gene expression alterations that reflect the presence of bronchial dysplasia using airway brushings in a similar cohort^14^.

Among the molecular subtypes, the Proliferative subtype is enriched with dysplastic PMLs from current smokers and is characterized by up regulation of metabolic (OXPHOS/ETC/TCA) and cell cycle pathways and down regulation of cilia-associated pathways. Previous work indicates increases in metabolic pathways in the airways of subjects with dysplastic lesions^14^, in PMLs adjacent to LUSC tumor^24^, and in smokers at high-risk for lung cancer^25^ as well as increases in proliferation (via Ki67 levels, as mentioned above) that have been utilized as an endpoint in lung cancer chemoprevention^26,27^. Identification of patients with Proliferative lesions may be useful to enrich lung cancer chemoprevention trials with high-risk subjects or to identify patients who would benefit from more frequent lung cancer screening. The Inflammatory subtype is predominated by PMLs from former smokers, but interestingly is not significantly enriched for dysplasia, despite similarly decreased expression of cilia-associated pathways, suggesting an abnormal epithelium. The Inflammatory subtype also shows increased expression of a gene module enriched for genes involved in inflammation and regulation of lymphocytes and leukocytes (Module 8). This gene module is also elevated in the Secretory subtype predominated by current smokers and increased expression of goblet cell markers. Interestingly, *IL1B* is part of this inflammation-related gene module, and inhibition of *IL1B* has recently been shown to reduce lung cancer incidence^28^.

Our prior work has extensively studied gene expression alterations in normal-appearing airway epithelium by profiling cells obtained via brushing the mainstem bronchus during bronchoscopy^7,14,29–34^. In the current study, we have both normal-appearing bronchial brushes and endobronchial biopsies collected during the same procedure, allowing us to identify gene expression differences in brushings, which indicate the presence of Proliferative subtype PMLs. In both the discovery and VC, applying a Proliferative subtype classifier (based on PML biopsy gene expression) to gene expression data from the brushings resulted in Proliferative subtype predictions that were very specific (91%) but not sensitive (31–38%). Brushes classified as Proliferative have increased expression of cell cycle pathways and decreased expression of cilia-associated genes, suggesting that they are more similar to squamous metaplasia than normal epithelium. Potentially, a subset of patients may harbor widespread airway damage that serves as a marker for Proliferative bronchial dysplasia leading to modest sensitivity, but high specificity. In other cases, the area of damage that gives rise to these Proliferative PMLs may be more localized, and therefore difficult to detect by brushing, contributing to decreased sensitivity. These findings suggest that therapeutics to target changes throughout the entire airway epithelium may be necessary in some subjects, whereas, more site-specific ablation may be more effective in certain cases. Another possibility and area of future research, is that a Proliferative subtype brush is a predictor of incident LUSC.

The molecular profiling of PMLs and the identification of gene co-expression modules also provides an opportunity to identify the molecular determinants of subsequent PML progression. One of the nine gene co-expression modules used to define the molecular subtypes was significantly decreased between biopsies that progress/persist compared to biopsies that regress within the Proliferative subtype in both the discovery and VC. The module contains genes involved in interferon signaling and antigen processing and presentation, and its expression was correlated with the abundance innate and adaptive immune cells via computational prediction. By immunofluorescent staining of formalin-fixed paraformaldehyde-embedded (FFPE) biopsy sections we confirmed that the progressive/persistent Proliferative lesions with low Module 9 GSVA scores had fewer CD163^+^ macrophages and CD8^+^ T cells and the CD8^+^T cells had a distinct localization pattern. These lesions also contained greater numbers of CD4^+^T cells, and it will be important in future work to assess if these cells are T regulatory cells promoting an immune suppressive environment.

The presence of tumor-associated macrophages with the polarized phenotypes (M1 as pro-inflammatory or M2 as anti-inflammatory) has been associated with lung cancer prognosis. The presence of predominantly M2 macrophages, marked by the expression of CD163, has been associated with worse survival. However, in the context of lung PMLs this relationship is not well studied. Our finding that regressive Proliferative PMLs have more CD163^+^ cells and increased expression of genes involved in interferon (IFN)γ signaling is consistent with observations in oral squamous cell carcinoma PMLs where the presence of CD163^+^ macrophages with active IFNγ signaling is associated with better outcomes^35^. In addition, we observed fewer CD8+ T cells and lower expression of human leukocyte antigen (HLA) class I genes and B2M in progressive/persistent lesions within the Proliferative subtype. Disruptions in proper T-cell-mediated immunosurveillance have been described in several studies showing that impaired HLA class I antigen processing and presentation including down regulation or loss of B2M^36,37^ and interferon signaling^38^ in lung tumors affects response and acquired resistance to checkpoint inhibitors. Lung tumors lacking an HLA-I complex had lower cytotoxic CD8^+^ lymphocyte infiltration, and this was also associated with lower levels of PD-L1. In addition, studies have also suggested negative impacts on efficacy of checkpoint inhibitors as well as survival in patients with LC that have tumors with increased CD4^+^ T cells expressing T-regulatory markers (FOXP3, CD25), resulting in immunosuppressive state suggested to hinder the recruitment and effector functions of CD8^+^ T cells^39,40^. Future DNA sequencing data on the PMLs profiled here may indicate heterozygous or homozygous loss of *B2M* or mutations in other genes in the interferon and antigen processing and presentation pathways; however, even in the case of acquired resistance, mutations and copy number changes could not explain the down regulation of these pathways across all subjects, suggesting that other epigenetic alterations or signaling pathways may have a role. Unraveling the mechanisms of innate and adaptive immune downregulation in this subset of PMLs will be important to identifying potential immunoprevention therapies.

Our data suggest that there are subtype-specific transcriptomic alterations predictive of subsequent LUSC PML progression that are the result of a lack of infiltrating immune cells in the lesion microenvironment. These data suggest that biomarkers for determining PML subtype and assessing immune infiltration may have utility for the detection of aggressive PMLs that require more intensive clinical management and genes altered in these PMLs may serve as lung chemoprevention candidates. These biomarkers could either be measured directly in PML tissue or a surrogate tissue such as bronchial airway epithelium. A benefit of biomarkers predicting aggressive PML behavior measured in surrogate tissue is the potential that these biomarkers might also predict the behavior of PMLs not directly observed during bronchoscopy. Future studies are needed to address the specific mechanism of impaired immunosurveillance in progressive/persistent lesions in the Proliferative subtype including single-cell sequencing, high coverage DNA sequencing, characterization of neoepitope burden, assessment of epigenetic alterations, and comprehensive characterization of the identified immune populations. Overall, our data support the potential for immunoprevention strategies to intercept the progression of PMLs to lung cancer.

## Methods

### Subject population and sample collection

Endobronchial biopsies and brushings were obtained from high-risk subjects undergoing lung cancer screening at ~1-year intervals by white light and autofluorescence bronchoscopy and computed tomography at Roswell. The bronchoscopy included visualization of the vocal cords, trachea, main carina, and orifices of the sub-segmental bronchi visible without causing trauma to the bronchial wall. All abnormal and suspicious areas are biopsied twice and the lung anatomic location is recorded (Supplementary Fig. 9, Supplementary Table 9). One biopsy was used for routine pathological evaluation and the other for molecular profiling. In addition, a brushing was obtained from a normal-appearing area of the left or right mainstem bronchus for research. Morphological criteria used to evaluate the biopsies are in accordance with World Health Organization (WHO) guidance^41^. Eligibility for screening includes either a previous history of aerodigestive cancer and no disease at the time of enrollment or age > 50, a current or previous history of smoking for a minimum exposure of 20 pack-years and at least one additional risk factor including moderate COPD (defined as forced expiratory volume (FEV1) < 70%), confirmed asbestos related lung disease or a strong family history of lung cancer (at least 1–2 first-degree relatives). All research specimens were stored in RNA Allprotect (Qiagen) and stored at −80°C.

Subjects were selected that had biopsies collected in repeat locations via serial bronchoscopies; however, after RNA isolation, samples from three subjects had a single biopsy and one subject had a single brushing. mRNA-seq was performed on a DC of samples comprising of endobronchial biopsies and brushes collected between 2010 and 2012 (*n* = 30 subjects, *n* = 197 biopsies, and *n* = 91 brushings). mRNA-seq was subsequently performed on a VC of samples comprising of endobronchial biopsies and brushes collected between 2012 and 2015 (*n* = 20 subjects, *n* = 111 biopsies, and *n* = 49 brushings). Biopsy progression/regression was defined for each biopsy based on the histology of the biopsy and the worst histology recorded for the same lung anatomic location in the future. Histology changes between normal, hyperplasia, and metaplasia were classified as “normal stable”, decreases in histological dysplasia grade or changes from dysplastic histology to normal/hyperplasia/metaplasia were classified as “regressive”, lack of future histological data was classified as “unknown”, and everything else was classified as “progressive/persistent.” The Institutional Review Boards at Boston University Medical Center and Roswell approved the study and all subjects provided written informed consent.

### RNA-Seq library preparation, sequencing, and data processing

Total RNA was extracted from endobronchial biopsies and bronchial brushings using miRNeasy Mini Kit or AllPrep DNA/RNA/miRNA Universal Kit (Qiagen). Sequencing libraries were prepared from total RNA samples using Illumina TruSeq RNA Kit v2 and multiplexed in groups of four using Illumina TruSeq Paired-End Cluster Kit. Each sample was sequenced on the Illumina HiSeq 2500 to generate paired-end 100-nucleotide reads. Demultiplexing and creation of FASTQ files were performed using Illumina CASAVA 1.8.2 or BaseSpace. Samples were aligned using hg19 and 2-pass STAR^42^ alignment. Gene and transcript level counts were calculated using RSEM^43^ using Ensembl v75 annotation. Quality metrics were calculated by STAR and RSeQC^8^. Samples were excluded were sex annotation did not correlate with gene expression across CYorf15A, DDX3Y, KDM5D, RPS4Y1, USP9Y, and UTY (*n* = 4 samples). Sample relatedness within a patient was confirmed using Peddy software^44^. Samples with a high-rate of heterozygosity (>3 standard deviations above the median) or samples with low relatedness to samples from the same patient (>3 standard deviations below the median) were removed from further analyses (*n* = 11 samples, two brushes, and nine biopsies). Samples were subsequently divided into the discovery and VC (as outlined above) and by tissue type (biopsy or brush). Subsequent sample and gene filtering was conducted separately on each set as follows: first, EdgeR^45^ was used to compute normalized data (library sizes normalized using TMM, trimmed mean of M-values, and log2 counts per million computed) and genes were excluded that either had an interquartile range equal to zero or a sum across samples equal or <1. Samples were excluded based on values > 2 standard deviations from the mean for more than one of the following criteria: (1) mean Pearson correlation with all other samples calculated across all filtered genes (2) the 1st or 2nd principal components calculated using the filtered gene expression matrix (3) transcript integrity number (TIN, computed by RSeQC). After sample filtering, gene filtering was recomputed as described above on the final set of high-quality samples. The data are available from NCBI’s Gene Expression Omnibus using the accession GSE109743.

### Derivation of molecular subtypes

The DC biopsies (*n* = 190 samples, *n* = 16653 genes) and brushes (*n* = 89 samples, *n* = 16058 genes) were used to derive the molecular subtypes. Two additional RNA-Seq data sets were used during the derivation of the molecular subtypes: the TCGA squamous cell carcinoma (LUSC) tumors^10^ (*n* = 471 samples, *n* = 17887 genes) and a dataset of tracheobronchial samples from mice treated with NTCU (*n* = 25 samples, *n* = 14897 genes). The mice develop lesions that are histologically and molecularly comparable to human lesions and that progress to LUSC and the samples represent a range of histology (normal, mild dysplasia, moderate dysplasia, severe dysplasia, CIS, and LUSC tumor) (Supplementary Materials and Methods). The mouse data are available from NCBI’s Gene Expression Omnibus using the accession GSE111091. Sample and gene filtering from the TCGA LUSC tumors and the mouse tissue were processed as described in the Supplementary Materials and Methods.

WGCNA^9^ was used with default parameters to derive modules of gene co-expression across the four data sets described above. Residual gene expression values adjusting for RNA quality (median TIN) and batch (Illumina flow cell) were used as input for WGCNA for the biopsy and brush data sets. For the mouse dataset, residual gene expression values adjusting for RNA quality (median TIN), mouse strain, and sample type (laser capture microdissected versus whole tissue) were used as input for WGCNA. For TCGA LUSC tumor samples, residual gene expression values adjusting for plate were used as input for WGCNA. Gene sets were created for each co-expression module for each dataset and then combined to create a compendium of gene sets. For each gene set in the compendium, the first principal component (PC1) was calculated across each z-score normalized dataset. For each data set, a matrix of absolute Pearson correlation coefficients based on PC1 values calculated for each gene sets in the compendium was computed and thresholds were set as follows: *r* > 0.85 was set to 1 and *r* ≤ 0.85 set to 0. The four matrices were subsequently summed, and gene sets derived from biopsy co-expression modules that were correlated to another non-biopsy-derived gene set across all data sets were retained (*n* = 9 modules retained). The genes defining the retained biopsy modules were required to be present in the biopsy module and at least in one of the correlated gene sets.

The filtering process above yielded a reduced set of genes (*n* = 3936) that was used to define the molecular subtypes in the biopsy data. The residual gene expression values across the reduced set of genes for the discovery biopsies were used as input for consensus clustering^46^. Consensus clustering was performed setting *k* (number of groups) to 10, the number of iterations to 1000, the subsampling to 80%, the clustering algorithm to partitioning around medoids, and the distance metric to Pearson correlation. The optimal value for *k* was 4 based on the relative change in area under the cumulative distribution function calculated based on the consensus matrix for each k.

### Molecular subtype predictor

The DC biopsies across the module genes (*n* = 3936) were used to derive a molecular subtype predictor. First, squared Pearson correlation coefficients were determined between each gene and the module eigengenes (PC1 for each of the nine modules). Genes were retained as part of a module if the squared correlation coefficient was the highest for the module in which it was assigned. The average squared Pearson correlation coefficient of the retained genes to the module eigengene was computed, and the number of genes chosen from each module for the predictor was inversely proportional to this metric. Second, genes with the highest squared correlation coefficients to the module eigengene were chosen to represent the module in the predictor. The 22 genes resulting from this analysis across the DC biopsy data were used to train a nearest centroid predictor using the pamr package with a threshold of zero and predict the molecular subtype across the VC biopsies. Prior to predicting the molecular subtype of these test sets, the training and test sets were combat^47^ adjusted and z-score normalized across combined training and test data. Using the methods described above we derived molecular subtypes using consensus clustering across the VC biopsies and compared these to the predicted subtypes.

### Identification of gene module biology

Biological processes and pathways enriched in each of the nine modules used to discover the molecular subtypes in the DC were identified using EnrichR^48^. Each module was separated into genes positively or negatively correlated with the module eigengene, the Ensembl IDs were converted to Gene Symbols using biomaRt, and the following databases were queried: GO Biological Process 2015, KEGG 2016, WikiPathways 2016, TargetScan microRNA, Transcription Factor PPIs, TRANSFAC and JASPAR PWMs, OMIM Disease, Reactome 2016, and Biocarta 2016. Processes/pathways with an FDR < 0.05 were considered to be significantly enriched. Modules are referred to being increased or decreased in each of the molecular subtypes based on the direction of change of the majority of the genes in the module.

### Identification of molecular subtype phenotypes

The molecular subtypes in the DC biopsies were annotated according to the behavior of each gene module by calculating whether or not module GSVA^13^ scores were significantly associated (FDR < 0.05) with a particular molecular subtype versus all other samples (two-level factor) using a linear mixed effects model with patient as a random effect (using the ‘duplicateCorrelation’ function) via limma. In addition, biological pathways and transcription factors associated with each subtype were identified using GSEA^49^ and mSigDB^50^ gene sets using genes ranked by the t-statistic for their association with each subtype. The ranked lists were created using the limma^51^ and edgeR^45^ packages to identify differentially expressed genes associated with subtype membership. Each linear model used voom-transformed^52^ data and included membership in the subtype of interest, batch, and RNA quality (TIN) as covariates and patient as a random effect. Pathways enriched in the ranked lists (FDR < 0.05) were used to annotate the molecular subtypes. FDR values for individual genes were derived from this analysis or analogous models using only samples of normal/hyperplasia histology or dysplasia histology.

For the DC and VC biopsies, residual gene expression values were used to predict smoking status, LUSC tumor subtype, and the relative abundance of epithelial and immune cells for each sample. Smoking status (current versus former/never) was predicted for each sample as described previously^14^. Smoking status was determined at each time point for each subject by calculating the mean of the prediction scores ( >0 for current prediction and <0 for former/never prediction) across all biopsies and brushes sampled. The LUSC tumor subtype was determined as described previously^11^ across the genes predictive of the LUSC molecular subtype^12^. The ESTIMATE algorithm^53^ was used to infer relative epithelial, stromal, and immune cell content. Immune cell type specific signatures from Bindea et al.^15^ and epithelial cell type specific signatures from Dvorak et al.^54^ were used to generate GSVA^13^ scores across samples for each signature. In addition, residual gene expression values calculated using log RPKM values were inputted into the xCell^16^ to infer relative abundances of 64 different cell types. The above categorical phenotypes along with additional clinical variables such as biopsy histology, subject, previous lung cancer history, sex, and biopsy progression/regression status were associated with molecular subtype using two-sided Fisher’s exact test.

In order to characterize the molecular alterations associated with lesion outcome, a linear mixed effects model was used to assess module GSVA score differences between progressive/persistent versus regressive lesions within each molecular subtype with patient as a random effect via limma. We estimated differences in the immune cell content (separately for xCell and Bindea et al.) between progressive/persistent versus regressive lesions in the Proliferative subtype via a linear mixed effects model correcting for epithelial cell content (‘Epithelial’ in xCell and ‘Normal mucosa’ in Bindea et al.) and patient as a random effect. We focused on cell types that were significantly different (FDR < 0.05) between progressive/persistent versus regressive lesions in the Proliferative subtype in both the DC and VC.

### Relationship between the biopsies and brushes

We wanted to quantify the predictive performance of the brush with regards to the presence of a biopsy of the Proliferative subtype. A subset of the 22-gene molecular subtype predictor was used to predict the presence or absence of the Proliferative subtype across the DC and VC brushes and biopsies. Specifically, we used eight genes (out of the 22) that corresponded to modules 4 through 7 (significantly up  or down regulated in the Proliferative subtype) to classify samples as Proliferative or not using the same methodology described above for the molecular subtype predictor. Sensitivity and specificity performance metrics were calculated based on the ability of a Proliferative subtype prediction in the DC or VC brushes to indicate the presence of at least one biopsy of the Proliferative subtype. In order to further understand the Proliferative subtype predictions in the brushes, we analyzed the behavior of the modules that define the Proliferative subtype in the DC biopsies (based on methods above) across the DC and VC brushes.

### Immunofluorescent staining and quantitation

Standard formalin fixation and embedding techniques were employed at Roswell where five-micron sections were cut from the FFPE samples used for the routine pathological evaluation at Roswell (Supplementary Table 7). Prior to staining, samples were de-waxed with xylene and rehydrated through a graded series of ethanol solutions. AR or citrate buffer was used for antigen retrieval, tissue was incubated with primary antibodies overnight at 4 °C and probed with secondary antibodies with fluorescent conjugates (Invitrogen Alexa Fluor 488, 594, 647) for 1 h at room temperature. Immunostaining was performed using the primary antibodies listed in Supplementary Table 10. Imaging was performed using an Aperio Slide Scanner for scoring and a Carl Zeiss Axio (×20 and ×40 objectives) and a Carl Zeiss LSM 710 NLO confocal microscope for capturing additional images. Digital slides were analyzed with the Definiens Tissue Studio (Definiens Inc.) for the enumeration of immunofluorescence staining. The enumeration of the immunofluorescence scored each stain including DAPI-positive cells. The enumeration was conducted on different regions (independent areas of tissue) present on a slide (1–5 regions/biopsy) for each biopsy. For each region, the percentage of positively staining cells for a given protein was calculated by dividing the number of positively stained cells by the total number of DAPI-positive cells. A binomial linear mixed effects model via the lme4 R package was used to assess differences in the percentages of cells staining positive for a given protein in each region between progressive/persistent versus regressive biopsies using the total cells stained in each region as weights and adjusting for the slide number as a random effect. The models were used across samples from the Proliferative subtype and across samples from the Proliferative subtype where the biopsy outcome (progressive/persistent versus regressive) agreed with the Module 9 GSVA score (scores < 0 are associated with progression/persistence and scores greater than 0 are associated with regression). Each region was also qualitatively scored as either positive or negative for having a distinct CD8 T-cell localization pattern where cells lined and were embedded within the epithelium.

### Reporting summary

Further information on research design is available in the Nature Research Reporting Summary linked to this article.

## Supplementary information

Supplementary Information

Description of Additional Supplementary Files

Supplementary Dataset 1

Raw Image File 1 for Figure 1E

Raw Image File 2 for Figure 1E

Raw Image File 3 for Figure 1E

Raw Image File 4 for Figure 1E

Raw Image File 5 for Figure 1E

Raw Image File 6 for Figure 1E

Raw Image File 7 for Figure 1E

Raw Image File 8 for Figure 1E

Raw Image File 9 for Figure 1E

Raw Image File 10 for Figure 1E

Raw Image File 11 for Figure 1E

Raw Image File 12 for Figure 1E

Raw Image File 13 for Figure 1E

Raw Image File 14 for Figure 1E

Raw Image File 15 for Figure 1E

Raw Image File 16 for Figure 1E

Raw Image File 17 for Figure 4D

Raw Image File 18 for Figure 4D

Raw Image File 19 for Figure 4D

Raw Image File 20 for Figure 4D

Raw Image File 21 for Figure 4D

Raw Image File 22 or Figure 4D

Raw Image File 23 or Figure 4D

Raw Image File 24 or Figure 4D

Raw Image File 25 or Figure 4D

Raw Image File 26 or Figure 4D

Raw Image File 27 or Figure 4D

Raw Image File 28 or Figure 4D

Reporting Summary

Source Data

## Data Availability

RNA sequencing data from human endobronchial biopsies and brushings has been deposited in the NCBI Gene Expression Omnibus under accession code GSE109743. RNA sequencing data from mouse lung samples treated with N-nitrosotris-(2-choroethyl)urea has been deposited in the NCBI Gene Expression Omnibus under accession code GSE111091. The source data underlying all figures and tables in the main text and supplementary information are provided as a Source Data file. All other data supporting the findings of this study are available within the article and its supplementary information files and from the corresponding author upon reasonable request. A reporting summary for this article is available as a Supplementary Information file.

## Code availability

All custom computer code is available at https://github.com/jbeane0428/Computer-code-for-PML-Molecular-Subtypes. Corresponding input and output data used in the computer code is available at 10.6084/m9.figshare.c.4432451.v1.
