# Supplementary material for: Molecular subtyping reveals immune alterations associated with progression of bronchial premalignant lesions
Source: Nat Commun. 2019 Apr 23;10:1856. doi: 10.1038/s41467-019-09834-2 (PMC6478943; doi:10.1038/s41467-019-09834-2)
Supplement: Supplementary file 1 — Supplementary Information [file 41467_2019_9834_MOESM1_ESM.pdf]

Molecular subtyping reveals immune alterations associated with progression of bronchial premalignant lesions

Beane et al.

Supplementary Materials and Methods

N-nitrosotris-(2-choroethyl)urea (NTCU) mouse sample collection and library preparation

We have previously collected and banked RNA from 40 fresh frozen whole lung sections (curls) and laser microdissected (LCM) tissue isolated with an Acrutus Pixcell II, from SWR/J and A/J mice treated with NTCU. Mice had been treated topically with 15 or 25 umol NTCU (25 ul of 40 mM NTCU for 15 or 25 weeks) as part of a study performed in accordance with IACUC approved protocol at Roswell Park Comprehensive Cancer Center (Roswell). Samples include examples of: normal (SWR/J n= 3 LCM & 3 curls & A/J n =2 LCM & 1 curl), metaplasia/mild dysplasia (SWR/J n=5 LCM & 2 curls), moderate dysplasia (SWR/J n=7 LCM & 4 curls & A/J n=2 LCM & 1 curls), and severe dysplasia (SWR/J n=3 LCM & 2 curls), and *carcinoma in situ*/LUSC (A/J n=2 LCM & 2 curls). Samples were extracted using the Qiagen mi-RNAeasy kit according to manufacturer's protocol. Sequencing libraries were prepared from total RNA samples using Illumina® TruSeq® RNA Sample Preparation Kit v2. Each sample was sequenced five per lane on the Illumina® HiSeq 2500 to generate single-end 50-nucleotide reads.

Histological Classification of the NTCU Mouse Samples that underwent RNA Sequencing (n= collected/ n=passed QC after sequencing)							
Mouse Stains	Sample Type	Normal	Mild Dysplasia	Moderate/ Severe Dysplasia	Severe Dysplasia	CIS/SCC Tumor	Total
A/J	LCM	2/2	-	2/2	-	2/1	6/5
A/J	Curls	1/1	-	1/1	-	2/1	4/3
SWR/J	LCM	4/1	5/3	7/3	3/1		19/8
SWR/J	Curls	3/3	2/2	4/3	2/1		11/9
						Total	40/25
Mean RIN values (SD)		4.0(1.8)	3.8(0.5)	3.3(0.6)	2.55(0.1)	3.4(1.2)	

NTCU mouse data processing

De-multiplexing and creation of FASTQ files were performed using Illumina CASAVA 1.8.2. Trimmomatic was used to trim adapter sequences as well as to trim reads of poor quality using the following parameters: ILLUMINACLIP:TruSeq3-SE.fa:2:30:10, LEADING:20, TRAILING:20, SLIDINGWINDOW:4:20, and MINLEN:20. After trimming, greater than 99% of reads were retained in all samples. Samples were subsequently aligned using mm9 and 2-pass STAR[1] alignment. Gene and transcript level counts were calculated using RSEM[2] and Ensembl annotation. Quality metrics were calculated by STAR and RSeQC[3]. Initially, 15 samples were removed based on percent of uniquely aligned reads (compared to total reads) less than 15%. Subsequent sample and gene filtering was conducted separately on each set as follows: First, EdgeR[4] was used to compute normalized data (library sizes normalized using TMM, trimmed mean of M-values, and log2 counts per million computed) and genes were excluded that either had an interquartile range equal to zero or a sum across samples equal or less than 1. Samples were excluded based on values greater than 2 standard deviations from the mean for 1) mean Pearson correlation with all other samples calculated across all filtered genes 2) the 1st or 2nd principal components calculated using the filtered gene expression matrix 3) transcript

integrity number (TIN, computed by RSeQC). After sample filtering, gene filtering was recomputed as described above on the final set of high-quality samples. The data are available from NCBI's Gene Expression Omnibus using the accession GSE111091[http://www.ncbi.nlm.nih.gov/geo/query/acc.cgi?acc=GSE111091].

Immunofluorescent quantification of cell type and proliferative markers

Basal and ciliated cell type markers (KRT5 and TUB1A1) and the proliferative marker (KI67) were manually enumerated for all epithelium within a biopsy in reference to DAPI staining, with a minimum of 500 cells counted per biopsy. The enumeration was conducted on different regions (independent areas of tissue) present on a slide (1-4 regions/biopsy) for each biopsy. A percent of positively stained cells was calculated for each marker in each region enumerated. A binomial mixed effects model via the lme4 R package was used to assess differences in the percentages of cells staining positive for a given protein in each region between the molecular subtypes using the total cells stained in each region as weights and adjusting for patient as a random effect.

TCGA SCC tumors data processing

Log2 transcript per million data across 20,500 genes from 476 LUSC tumors was obtained from Campbell[5] *et al*. Genes were excluded that either had an interquartile range equal to zero or a sum across samples equal or less than 1. Samples were excluded based on values greater than 2 standard deviations from the mean for more than one of the following criteria: 1) mean Pearson correlation with all other samples calculated across all filtered genes 2) the 1st or 2nd principal components calculated using the filtered gene expression matrix 3) transcript integrity number (TIN, computed by RSeQC). After sample filtering, gene filtering was recomputed as described above (n=17,887 genes) on the final set of high-quality samples (n=471 tumors).

Software Packages

Custom scripts: R-3.3.2, GSVA v1.22.4, Limma v3.30.13, ggplot2 v3.0.0, SummarizedExperiment v1.4.0, edgeR v3.16.5, ConsensusClusterPlus v1.38.0, biomaRt v2.30.0, estimate v1.0.13, heatmap3 v1.2.2, pamr v1.55, lme4 v1.1-13

RNA-seq processing pipeline software for human data (https://github.com/joshua-d-campbell/nf-RNA_Seq_Preprocess v1.0): nextflow v0.24.4, star v2.5.2b, rsem v1.3.0, FastQC v0.11.3, Picard tools v2.8.0, GATK v3.5, rseqc v2.6.4, multiqc v0.9, samtools v1.4

RNA-seq processing software for mouse data: star v2.4.2a, samtools v1.2, picard v1.138, trimmomatic v0.33, rseqc v2.6.1, rsem v1.2.23

Supplementary Tables

	Discovery Cohort	Validation Cohort
Genomic smoking status over time	Number of Subjects	Number of Subjects
Current	9	9
Former	10	5
Current->Former	7	4
Former->Current	3	2
Current->Former->Current	1	0

Supplementary Table 1. Genomic smoking status over time by subject. The smoking status of each subject at each time point was computed based on a previously published smoking-associated gene signature[6] (see methods for details). The rows indicate the smoking status across all time points sampled for each patient. The -> symbol indicates changes in smoking status over time. There is not a statistical difference between the distribution of subjects in the smoking status categories between the discovery and validation cohorts by a two-sided Fisher's exact Test (p=0.90). Source data are provided as a Source Data file.

Variable	Discovery Cohort		Validation Cohort		P-value	
Sample Type	Biopsies	Brushes	Biopsies	Brushes	Biopsies	Brushes
Batch/Illumina Flow Cell Assignment					<2e-16	<2e-16
1	19/190 (10)	12/89 (13.5)	0/105 (0)	0/48 (0)		
2	18/190 (9.5)	13/89 (14.6)	0/105 (0)	0/48 (0)		
3	22/190 (11.6)	9/89 (10.1)	0/105 (0)	0/48 (0)		
4	19/190 (10)	10/89 (11.2)	0/105 (0)	0/48 (0)		
5	29/190 (15.3)	2/89 (2.2)	0/105 (0)	0/48 (0)		
6	24/190 (12.6)	8/89 (9.0)	0/105 (0)	0/48 (0)		
7	20/190 (10.5)	11/89 (12.4)	0/105 (0)	0/48 (0)		
8	17/190 (8.9)	14/89 (15.7)	0/105 (0)	0/48 (0)		
9	22/190 (11.6)	10/89 (11.2)	0/105 (0)	0/48 (0)		
10	0/190 (0)	0/89 (0)	22/105 (10.7)	9/48 (18.8)		
11	0/190 (0)	0/89 (0)	19/105 (9.3)	10/48 (20.8)		
12	0/190 (0)	0/89 (0)	21/105 (10.2)	10/48 (20.8)		
13	0/190 (0)	0/89 (0)	20/105 (9.8)	12/48 (25)		
14	0/190 (0)	0/89 (0)	23/105 (11.2)	7/48 (14.6)		
Total Reads	45.5e+6 (7.2e+6)	45.3+-6 (7.9e+6)	42.9e+6 (6.3e+6)	42.6e+6 (4.9e+6)	1.50E-03	0.014
Median Transcript Integreity Number (TIN)	78.4 (1.9)	72.6 (3.4)	76.3 (2.0)	72.3 (2.8)	2.08E-10	0.59
Percent Uniquely Mapped	90.1 (2.9)	89.0 (5.9)	83.9 (9.6)	87.6 (4.9)	2.15E-09	0.15

Supplementary Table 2. Batch information and alignment statistics on samples in the Discovery and Validation cohorts. Statistical tests between the discovery and validation cohorts were performed using two-sided Fisher's exact tests for categorical variables and two-sided Student's T-tests for continuous variables. Percentages are reported for categorical variables and mean and standard deviations are reported for continuous variables. Source data are provided as a Source Data file.

Module Number	Number of Genes	Biological Pathways Associated with Module Genes	Key Genes	FDR for Difference between Molecular Subtypes
1	514	Extracellular Matrix / Cell Adhesion	Collagens, Lamins, TGFb	2.7E-36
2	939	mRNA processing and splicing	RBMs & SRSF	7.2E-05
3	20	Transcriptional regulation in response to stimuli - (AP-1) Immediate-early response genes	JUN & FOS	1.9E-01
4	64	OXPHOS / ETC / TCA	COXs & NDUFs	3.3E-07
5	209	Cell Cycle / DNA replication / DNA repair	PCNA, TOP2A, CDC, AURK, RAD, XRCC	2.0E-31
6	1295	Cilium organization and assembly	FOXJ1, DYNC	6.6E-57
7	180	Ribosomal Proteins/ Translation	RPLs & RPSs	1.9E-13
8	603	Immune Activation and Inflammatory Response (leukocyte/lymphocyte regulation)	CD8A, CD86, GATA, STAT, IL1B, CD163, CD68	3.3E-07
9	112	Interferon signaling and Antigen Processing and Presentation	SP100, HLAs, STAT1	1.3E-02

Supplementary Table 3. Summary of biological characteristics of the gene modules. For each gene module, the following characteristics are listed: the module number, the number of genes in the module, the biological pathways enriched in each gene module, select genes from the module, and an FDR value for the difference in GSVA scores for each module between the molecular subtypes (within the discovery cohort biopsies) are reported. The FDR value for the difference between molecular subtypes was calculated using a linear mixed model with molecular subtype as the main effect and patient as a random effect. Source data are provided as a Source Data file.

Module Number	Number of Unique Gene Symbols	Correlation with Module Eigengene	Database	Category	P-value	FDR	Genes
1	40	Negative	WikiPathways_2016	Benzo(a)pyrene metabolism_Homo sapiens_WP696	6.17E-07	1.85E-05	AKR1C1,AKR1C3,AKR1C2
1	40	Negative	Reactome_2016	Glutathione synthesis and recycling_Homo sapiens_R-HSA-174403	0.00034956	0.0085643	GCLC,CNDP2
1	467	Positive	GO_Biological_Process_2015	extracellular matrix organization (GO:0030198)	2.79E-46	5.16E-43	COL18A1,SPARC,ITGB5,COL14A1,ELN,COL12A1,ICAM4,LAMC1,LOXL1,ADAMTS2,EFEMP2,EFEMP1,TIMP2,KDR,CSGALNACT1,PDGFRA,POSTN,DST,VWF,LEPRE1,MMP2,BGN,HSPG2,MMP11,CRISPLD2,COL4A4,COL6A2,COL6A1,PXDN,COL4A6,COL8A2,ITGA7,COL4A5,COL6A3,COL8A1,GAS6,DDR2,RAMP2,LAMA2,COL13A1,LAMA4,LAMA3,LRP4,PDGFA,LTBP3,NID1,FBLN2,THSD4,FBLN5,NCAM1,JAM3,TGFB2,FOXF2,FOXF1,WNT3A,TGFB3,FN1,LAMB1,MFAP5,COL1A1,MFAP4,SMOC2,COL3A1,COL1A2,COL5A1,LEPREL2,COL7A1,COL5A3,MFAP2,COL5A2,RECK,FBN1
1	467	Positive	WikiPathways_2016	Focal Adhesion-PI3K-Akt-mTOR-signaling pathway_Mus musculus_WP2841	1.68E-16	2.03E-14	FLT1,LAMA2,ITGB5,EPAS1,IRS1,LAMA4,LAMA3,PDGFA,PIK3R1,LAMC1,EGFR,GHR,KDR,NGFR,PDGFRA,ANGPT2,ANGPT1,VWF,F2R,FN1,LAMB1,GNG11,COL1A1,COL3A1,KITLG,COL1A2,COL5A1,COL4A4,COL5A3,COL6A2,KIT,COL5A2,COL4A6,ITGA7,TEK,FGFR4,FGFR1
1	467	Positive	GO_Biological_Process_2015	regulation of cell adhesion (GO:0030155)	7.40E-13	1.24E-10	SEMA5A,LAMA2,LDB1,ROCK2,LAMA4,LAMA3,LEF1,PIK3R1,NID1,FBLN2,DLL1,CX3CL1,PTPRG,CYTH3,NUAK1,PODXL,KDR,DACT2,EDIL3,WNT4,JAG2,TGFB2,ANGPT2,ANGPT1,FOXF1,WNT3A,LAMB1,SMAD7,COL1A1,SMOC2,BCL2,SNAI2,COL8A1,TEK
1	467	Positive	GO_Biological_Process_2015	lung development (GO:0030324)	4.30E-07	1.85E-05	PDGFRA,ADAMTS2,SPARC,FOXF1,CRISPLD2,EPAS1,HEG1,PDPN,GPC3,TBX4,EGFR,GLI3
1	467	Positive	Reactome_2016	Cell-Cell communication_Homo sapiens_R-HSA-1500931	2.39E-06	8.34E-05	KIRREL,CADM1,DST,LAMA3,PIK3R1,PARVB,NTN1,CDH5,CLDN5,CDH2,CDH11,PVRL3,FERMT2,SPTBN1
2	686	Positive	WikiPathways_2016	mRNA Processing_Homo sapiens_WP411	1.17E-12	2.14E-10	SFSWAP,NXF1,PABPN1,SNRNP70,SF3B1,SRSF10,RBM5,RBM17,RBM39,FUS,METTL3,CLK4,PTBP2,CLK2,CLK1,SFPQ,HNRNPH1,PRPF3,SRSF2,SRSF4,SRSF5,SREK1,SRSF6,SRSF7,SUGP2
2	686	Positive	GO_Biological_Process_2015	RNA splicing (GO:0008380)	7.70E-13	8.11E-10	ZCCHC8,RBM28,DDX5,RBM25,AKAP17A,RNPC3,U2AF1L4,PNN,PABPN1,SNRNP70,PCF11,TRA2A,SRSF10,SF3B1,SRSF11,RBM5,SRRM2,RBM39,RBM17,CPSF7,PRPF38B,ZRANB2,FUS,THOC1,PRPF39,SFPQ,DDX39B,HNRNPH1,PRPF3,SRSF2,LUC7L3,ACIN1,SRSF4,SNRNP48,SRSF5,SREK1,SRSF6,SRSF7,SUGP2,SF1
2	686	Positive	KEGG_2016	Spliceosome_Homo sapiens_hsa03040	5.92E-09	1.08E-06	TCERG1,RBM17,PRPF38B,DDX5,RBM25,DDX42,THOC1,U2SURP,U2AF1L4,DDX39B,SNRNP70,PRPF3,TRA2A,SRSF2,ACIN1,SRSF4,SRSF5,SRSF6,SRSF7,SF3B1,SRSF10
3	20	Positive	GO_Biological_Process_2015	response to cAMP (GO:0051591)	2.58E-10	1.73E-07	EGR1,JUN,DUSP1,FOSB,FOS,JUNB
3	20	Positive	GO_Biological_Process_2015	response to extracellular stimulus (GO:0009991)	4.51E-07	3.02E-05	NR4A2,EGR1,JUN,ZFP36,FOS,SLC25A25
4	64	Positive	WikiPathways_2016	Electron Transport Chain_Homo sapiens_WP111	1.49E-48	2.68E-47	NDUFB8,COX7B,NDUFB7,NDUFB10,NDUFB6,NDUFA12,NDUFB4,NDUFB3,NDUFB2,UQCR11,COX7A2,ATP5I,UQCR10,COX6A1,COX5B,COX5A,UQCRFS1,COX8A,NDUFA4,NDUFA1,NDUFC2,ATP5F1,COX6C,ATP5J2,COX6B1,UQCRQ,NDUFS6,NDUFS3
4	64	Positive	KEGG_2016	Oxidative phosphorylation_Homo sapiens_hsa00190	3.19E-47	7.99E-46	COX7B,NDUFB8,NDUFA13,NDUFB7,NDUFB10,NDUFB6,NDUFA12,NDUFB4,NDUFB3,NDUFB2,UQCR11,COX7A2,ATP5I,UQCR10,COX6A1,COX5B,COX5A,UQCRFS1,COX8A,NDUFA4,NDUFA1,NDUFC2,ATP5F1,COX6C,ATP5J2,COX6B1,UQCRQ,NDUFS6,NDUFS3
4	64	Positive	Reactome_2016	The citric acid (TCA) cycle and respiratory electron transport_Homo sapiens_R-HSA-1428517	3.64E-43	3.82E-41	NDUFB8,COX7B,NDUFA13,NDUFB7,NDUFB10,NDUFB6,NDUFA12,NDUFB4,NDUFB3,NDUFB2,UQCR11,ATP5I,UQCR10,COX6A1,COX5B,COX5A,UQCRFS1,COX8A,NDUFA4,NDUFA1,NDUFC2,ATP5F1,COX6C,ATP5J2,COX6B1,UQCRQ,NDUFS6,NDUFS3
5	209	Positive	Reactome_2016	Cell Cycle_Homo sapiens_R-HSA-1640170	3.45E-88	1.29E-85	TOP2A,FEN1,ERCC6L,ZWILCH,NCAPG2,HJURP,CASC5,BUB1B,FOXM1,BRCA2,SMC4,LMNB1,CKS1B,CDC20,PTTG1,NUF2,CHEK1,MYBL2,SPDL1,SKP2,GTSE1,TMPO,RFC5,BORA,RFC2,H2AFX,KIF23,ESCO2,MASTL,CKAP5,CDC25A,CCNA2,DBF4,ESPL1,CCNE2,MCM3,BIRC5,MCM4,MCM5,KIF2C,KIF20A,PCNA,HDAC1,CDCA5,NCAPG,CDCA8,LIN9,HMMR,PKMYT1,TYMS,NCAPH,SKA1,AURKB,SKA2,AURKA,CCNB2,BRIP1,CCNB1,ORC6,CDC45,ORC1,E2F1,E2F2,BUB1,PLK4,GINS1,CDT1,GINS2,CENPW,RRM2,UBE2C,RCC2,PLK1,GINS4,CDC7,CDC6,NDC80,ZWINT,DHFR,CENPE,TPX2,CENPF,KIF18A,RAD51,CENPH,CENPI,CENPK,CDK1,CENPN,NCAPD2,CENPO,SPC24,MAD2L1
5	209	Positive	WikiPathways_2016	Cell Cycle_Homo sapiens_WP179	8.44E-34	4.94E-32	PCNA,HDAC1,BUB1B,PKMYT1,CDC20,CCNB2,CCNB1,ORC6,CDC45,PTTG1,ORC1,CHEK1,E2F1,E2F2,SKP2,BUB1,PLK1,CDC7,CDC6,CDC25A,CCNA2,DBF4,ESPL1,CCNE2,CDK1,MCM3,MCM4,MCM5,MAD2L1
5	209	Positive	Reactome_2016	E2F mediated regulation of DNA replication_Homo sapiens_R-HSA-113510	5.80E-18	1.20E-16	CDT1,RRM2,PCNA,CDC6,TYMS,CDC25A,DHFR,CCNB1,ORC6,CDC45,ORC1,CDK1,E2F1
5	209	Positive	Reactome_2016	DNA Repair_Homo sapiens_R-HSA-73894	3.10E-13	4.17E-12	FANCI,RFC5,XRCC6,FEN1,PCNA,RFC2,XRCC2,H2AFX,FANCA,BRCA2,RAD51AP1,CCNA2,BRIP1,RAD51,EME1,KIAA0101,FANCD2,UBE2T,CHEK1,TIMELESS,KPNA2,DTL
6	1008	Positive	GO_Biological_Process_2015	cilium organization (GO:0044782)	4.24E-48	1.19E-44	SPAG16,TTC26,C2CD3,TRAF3IP1,IFT172,CBY1,LRRC6,HSPB11,RPGR,TCTN2,TMEM107,HYDIN,NEK1,BBS9,IFT57,CC2D2A,BBS5,BBS4,IQCB1,DYNC2H1,DNAI2,DNAAF3,IFT140,KIF24,RFX3,IFT80,NME5,RPGRIP1L,WDR35,GPR162,RP1,FAM161A,IFT81,WDPCP,ZMYND10,MNS1,IFT46,ARL6,TTBK2,DYNC2LI1,IFT74,ARL13B,KIF3A,TMEM67,NPHP1,DMD,BBS2,BBS1,CCDC176,WDR19,FOXJ1,FUZ,BBIP1,TMEM231,IFT20,TTC8,TMEM17,TTC30B,MKS1,B9D1,B9D2,CEP41,CLUAP1
6	1008	Positive	Reactome_2016	Assembly of the primary cilium_Homo sapiens_R-HSA-5617833	2.30E-40	1.79E-37	TTC26,C2CD3,TRAF3IP1,IFT172,CETN2,CCP110,HSPB11,IFT52,TUBA1A,TCTN2,CNTRL,TCTN1,BBS9,CNGA4,CEP97,KIFAP3,IFT57,CC2D2A,BBS5,BBS4,DYNC2H1,IQCB1,HSP90AA1,IFT140,KIF24,IFT80,RPGRIP1L,WDR34,DYNLL1,WDR35,LZTFL1,IFT88,IFT43,IFT81,BBS12,AKAP9,IFT46,SDCCAG8,UNC119B,ARL6,ARL3,TTBK2,DYNC2LI1,IFT74,ARL13B,KIF3A,TMEM67,TTC21B,NPHP1,TCTEX1D2,NPHP4,BBS2,BBS1,ODF2,WDR19,BBIP1,TUBB4B,IFT20,TTC8,TTC30B,WDR60,ALMS1,MKS1,B9D1,DYNLRB2,B9D2,CEP41,CLUAP1
7	161	Positive	Reactome_2016	Eukaryotic Translation Elongation_Homo sapiens_R-HSA-156842	4.67E-177	1.89E-174	RPL4,RPL5,RPL30,RPL3,RPL32,RPL31,RPLP1,RPL34,RPLP0,RPL10A,RPL8,RPL9,RPL6,RPL7,EEF1B2,RPS15,RPS4X,RPS14,RPL7A,RPS17,RPS16,RPL18A,RPS19,RPS18,RPL36,RPLP2,RPL35,RPL38,RPL37,RPS11,RPS10,RPL39,RPS13,RPS12,RPS9,RPL21,RPS7,RPS8,RPL23,RPS5,RPL22,RPS6,RPL13A,RPS3A,RPSA,EEF1A1,EEF1G,EEF1D,RPL37A,RPL24,RPL27,RPL26,RPL29,RPL28,UBA52,EEF1A1P5,RPL10,RPL12,RPL11,RPL36A,RPS15A,RPL14,RPS3,RPL13,RPL15,RPS2,RPL18,RPS27A,RPL17,RPL19,RPL41,RPL35A,RPL23A,RPS25,RPS28,RPS27,RPS29,RPL27A,RPS20,FAU,RPS21,RPS24,RPS23
7	161	Positive	WikiPathways_2016	Cytoplasmic Ribosomal Proteins_Homo sapiens_WP477	2.58E-160	1.14E-158	RPL4,RPL5,RPL30,RPL3,RPL32,RPL31,RPLP1,RPL34,RPLP0,RPL10A,RPL8,RPL9,RPL6,RPL7,RPS15,RPS4X,RPL7A,RPS14,RPS17,RPS16,RPL18A,RPS19,RPS18,RPL36,RPL35,RPLP2,RPL38,RPL37,RPS11,RPS10,RPL39,RPS13,RPS12,RPS9,RPL21,RPS7,RPS8,RPL23,RPS5,RPL22,RPS6,RPL13A,RPS3A,RPSA,RPL37A,RPL24,RPL27,RPL26,RPL29,RPL28,UBA52,RPL10,RPL12,RPL11,RPL36A,RPS15A,RPL14,RPS3,RPL13,RPL15,RPS2,RPL18,RPS27A,RPL17,RPL19,RPL41,RPL35A,RPL23A,RPS25,RPS28,RPS27,RPS29,RPL27A,RPS20,FAU,RPS21,RPS24,RPS23
8	593	Positive	Reactome_2016	Immune System_Homo sapiens_R-HSA-168256	2.04E-50	1.44E-47	FCN1,CD86,IFITM2,CSF3R,NCF1,WIPF1,NCF2,NCF4,TRAC,ICAM3,UBE2D1,IFI30,ACTB,IL27RA,ICAM1,IL18RAP,GRAP2,CLEC5A,C3AR1,AP1S2,TRIM25,PIP4K2A,GBP5,CD96,CISH,NUP210,PRKCB,CD300A,SLC11A1,TAP2,CYBB,OSCAR,CLEC4A,TYROBP,CD8B,CD8A,BTK,ELMO1,PRKCQ,TLN1,CLEC4E,CARD11,CSF1R,TXK,C5AR2,LY96,CSF2RB,RASGRP2,MEFV,RASAL3,RASGRP1,CSF2RA,RASGRP4,KLRK1,CLEC7A,INPP5D,NLRP3,NCKAP1L,SLAMF6,CD14,ICOS,CD300LF,PAG1,STAT5A,STAT5B,TNFSF14,CD160,SH2D1A,SIGLEC10,LY86,PTPRC,SELL,AGO4,TREML2,CD28,CD27,GRB2,IL7R,LAT,ITK,ITGAM,SH3KBP1,ARPC1B,ITGB2,PIK3CD,CD3G,PTPRJ,ARRB2,ITGAL,CD3E,CD3D,TREM1,SIRPB1,TNFSF13B,PSTPIP1,FCGR3A,CASP4,CASP1,CD36,AMICA1,JAK3,CCR2,HAVCR2,FCER1G,ITGA4,IL16,GAB2,TNFRSF1B,IL17RA,APBB1IP,TLR1,LAT2,FGR,HCK,ZAP70,LCK,IRF4,IL1B,ATP6V1B2,TLR8,IRF8,LCP2,LTB,HCST,TLR4,IL9R,TLR2,C1QB,LILRA6,C1QA,PTAFR,WAS,NLRC4,IL2RG,CBL,LILRA2,ADCY7,TANK,FYB,LILRA5,ADRBK1,PTK2B,FYN,IL12RB1,FCGR1A,LAIR1,MICB,TRAT1,CARD9,OSM,LILRB1,LILRB2,LILRB3,LILRB4,FCGR2A,CAMK4,IL2RB,PTPN6,SIGLEC1,CD247,PIK3AP1,FCGR2B,IL18R1,SIPA1,C1QC
8	593	Positive	GO_Biological_Process_2015	leukocyte activation (GO:0045321)	9.53E-48	3.04E-44	CD86,ITK,ITGAM,SPI1,CSF1,DOCK8,GIMAP1,PIK3CD,CD3G,PTPN22,IKZF1,ITGAL,CD3E,CD3D,PIK3CG,ICAM1,PREX1,AMICA1,JAK3,FCER1G,ITGA4,PRKCB,SLC11A1,NFAM1,RHOH,LAX1,UNC13D,LAT2,TLR1,ZAP70,TYROBP,CD8B,IRF4,LCK,CD8A,BTK,TLR8,CD48,LCP2,LCP1,DOCK2,TLR4,TLR2,SELPLG,TXK,TCF7,WAS,CXCR4,GATA3,RASGRP1,AIF1,STX11,KLRK1,CLEC7A,CCL5,CXCR2,IL21R,CCL3,PTK2B,FYN,FNIP1,MICB,SLAMF1,STAT5A,STAT5B,TGFB1,TNFSF14,BCL11B,LILRB1,CD2,PTPRC,IMPDH1,GPR183,CAMK4,TREML2,CD7,CD28,IL7R,LAT,MYO1F,IL18R1,GAPT
8	593	Positive	GO_Biological_Process_2015	lymphocyte activation (GO:0046649)	4.34E-39	3.46E-36	CD86,ITK,ITGAM,SPI1,DOCK8,GIMAP1,PIK3CD,CD3G,PTPN22,IKZF1,CD3E,ITGAL,CD3D,PIK3CG,ICAM1,PREX1,AMICA1,JAK3,ITGA4,PRKCB,NFAM1,SLC11A1,RHOH,LAX1,UNC13D,LAT2,ZAP70,CD8B,LCK,CD8A,IRF4,BTK,CD48,LCP1,DOCK2,TLR4,TXK,TCF7,WAS,CXCR4,GATA3,STX11,KLRK1,CLEC7A,IL21R,PTK2B,FYN,FNIP1,MICB,SLAMF1,STAT5A,STAT5B,TGFB1,TNFSF14,BCL11B,LILRB1,CD2,PTPRC,IMPDH1,GPR183,CD7,TREML2,CD28,IL7R,LAT,IL18R1,GAPT
8	593	Positive	GO_Biological_Process_2015	inflammatory response (GO:0006954)	4.87E-33	1.94E-30	NRROS,SERPINA1,NCF1,CSF1,ITGB2,PIK3CD,ITGAL,PIK3CG,PSTPIP1,MMP25,IL18RAP,CCRL2,C3AR1,PROK2,OLR1,CCR7,CCR2,KDM6B,NFAM1,SLC11A1,CYBB,TNFRSF1B,TLR1,HCK,ADORA2A,IL1B,CHI3L1,TLR8,ADAM8,PRKCQ,S100A9,S100A8,TLR2,CSF1R,CEBPB,C5AR2,PTAFR,CXCR4,LY96,FPR2,GATA3,NLRC4,AOAH,MEFV,RASGRP1,AIF1,PTGS1,CXCR1,CLEC7A,CCL5,CXCR2,CCL4,CCL3,NLRP3,CD14,FCGR1A,CCL18,CCR1,STAT5B,CD163,TGFB1,LY86,EMR2,THEMIS2,CAMK4,SIGLEC1,LAT
8	593	Positive	GO_Biological_Process_2015	regulation of T cell activation (GO:0050863)	2.71E-32	9.59E-30	CD86,CD83,TRAC,CD3G,PTPN22,IKZF1,CD3E,ITGAL,CD3D,TNFSF13B,SPN,SIT1,GRAP2,TNFAIP8L2,VSIG4,JAK3,CCR2,CD300A,LAX1,ZAP70,ADORA2A,LCK,IRF4,IL1B,ADAM8,PRKCQ,CARD11,SASH3,GATA3,CORO1A,AIF1,KLRK1,TESPA1,CCL5,NCKAP1L,FYN,IL12RB1,ICOS,PAG1,STAT5A,STAT5B,TGFB1,TNFSF14,LILRB1,LILRB2,CD2,PTPRC,CD5,CAMK4,CD28,PTPN6,GRB2,CD247,RLTPR,IL7R,LAT
9	110	Positive	Reactome_2016	Interferon Signaling_Homo sapiens_R-HSA-913531	4.33E-51	1.19E-48	SP100,CIITA,IFI6,UBE2L6,ADAR,IFI35,IFIT3,SOCS1,GBP1,B2M,TRIM21,GBP4,HLA-DQA1,TRIM22,HLA-DPA1,STAT1,DDX58,MX1,HLA-B,HLA-C,EIF2AK2,ISG15,HLA-A,HLA-F,PML,PSMB8,HLA-E,BST2,IFI27,OAS2,IRF1,OAS3,HLA-DPB1,TRIM14,HLA-DRA,HLA-DRB1,HLA-DQB2,IRF9,HLA-DQB1
9	110	Positive	Reactome_2016	Interferon gamma signaling_Homo sapiens_R-HSA-877300	4.57E-40	6.30E-38	SP100,CIITA,SOCS1,GBP1,B2M,TRIM21,HLA-DQA1,GBP4,TRIM22,HLA-DPA1,STAT1,HLA-B,HLA-C,HLA-A,HLA-F,PML,HLA-E,OAS2,IRF1,OAS3,HLA-DPB1,HLA-DRA,TRIM14,IRF9,HLA-DQB2,HLA-DRB1,HLA-DQB1
9	110	Positive	Reactome_2016	Cytokine Signaling in Immune system_Homo sapiens_R-HSA-1280215	6.62E-39	6.09E-37	CIITA,SP100,IFI6,UBE2L6,ADAR,IFI35,PSMB10,IFIT3,SOCS1,LGALS9,GBP1,TRIM21,B2M,GBP4,HLA-DQA1,TRIM22,HLA-DPA1,STAT1,IL15,DDX58,MX1,HLA-B,EIF2AK2,HLA-C,ISG15,HLA-A,HLA-F,PSMB8,PML,PSMB9,HLA-E,BST2,IFI27,OAS2,IRF1,OAS3,PSME1,HLA-DPB1,PSME2,HLA-DRA,TRIM14,HLA-DRB1,HLA-DQB2,IRF9,HLA-DQB1
9	110	Positive	Reactome_2016	Immune System_Homo sapiens_R-HSA-168256	1.41E-36	9.72E-35	CIITA,ADAR,UBE2L6,IFI35,IFIT3,IFIH1,RNF114,TRIM69,LGALS9,HLA-DOA,TRIM21,B2M,TRIM22,HERC6,HLA-DPA1,DTX3L,IL15,DDX58,HLA-B,HLA-C,TAP1,HLA-A,HLA-F,TAPBP,HLA-E,IFI27,OAS2,OAS3,IRF1,PSME1,TRIM14,PSME2,HLA-DQB2,IRF9,HLA-DQB1,SP100,NLRC5,IFI6,PSMB10,C2,HLA-DMA,RNF213,HLA-DMB,SOCS1,GBP1,HLA-DQA1,GBP4,CD74,STAT1,MX1,EIF2AK2,ISG15,PML,PSMB8,PSMB9,BST2,HLA-DPB1,HLA-DRA,HLA-DRB1
9	110	Positive	KEGG_2016	Antigen processing and presentation_Homo sapiens_hsa04612	1.46E-30	1.07E-28	CD74,CIITA,HLA-B,TAP1,HLA-C,HLA-A,HLA-F,TAPBP,HLA-E,HLA-DMA,HLA-DMB,HLA-DPB1,PSME1,HLA-DRA,PSME2,HLA-DOA,B2M,HLA-DQA1,HLA-DRB1,HLA-DQB1,HLA-DPA1
9	110	Positive	Reactome_2016	Interferon alpha/beta signaling_Homo sapiens_R-HSA-909733	6.87E-30	3.79E-28	STAT1,MX1,IFI6,HLA-B,HLA-C,ADAR,ISG15,HLA-A,IFI35,HLA-F,IFIT3,PSMB8,HLA-E,BST2,SOCS1,IFI27,OAS2,IRF1,OAS3,IRF9

Supplementary Table 4. Biological pathways enriched in each of the gene modules. Biological processes and pathways enriched in each of the nine modules used to discover the molecular subtypes in the discovery cohort were identified using EnrichR. Each module was separated into genes positively or negatively correlated with the module eigengene and the Ensembl IDs were converted to gene symbols using biomaRt, and the following databases were queried: GO Biological Process 2015, KEGG 2016, WikiPathways 2016, TargetScan microRNA, Transcription Factor PPIs, TRANSFAC and JASPAR PWMs, OMIM Disease, Reactome 2016, and Biocarta 2016. Processes/pathways with an FDR<0.05 were considered to be significantly enriched. Data S1 contains the complete results. Source data are provided as a Source Data file.

Variable	DC P-value	VC P-value
Genomic Smoking Status	2.71E-09	2.72E-04
Subject	9.66E-05	5.87E-03
Subject/Time	6.96E-04	1.40E-02
Histology	6.75E-03	9.99E-08
Location	2.57E-02	6.69E-01
Subject/Location	6.01E-02	1.95E-01
Asbestos Exposure	1.23E-01	7.47E-02
Lung Cancer History	1.32E-01	9.92E-01
Progression Status	1.60E-01	1.67E-05
High-risk Job	4.31E-01	8.30E-01
Sex	5.62E-01	8.90E-01
LUSC Tumor Subtype	9.99E-08	1.80E-06
COPD Status	1.62E-01	9.38E-03

Supplementary Table 5. Molecular subtype associations with clinical and biological characteristics within the discovery and validation cohort biopsies. Statistical tests within the discovery and validation cohorts were performed using two-sided Fisher's exact tests. DC = Discovery Cohort and VC = Validation Cohort. Source data are provided as a Source Data file.

	Discovery Cohort Biopsies (n=190)				Validation Cohort Biopsies (n=105)			
**Variable**	No LC History	LC History - LUSC	LC History - Other	P-Value	No LC History	LC History - LUSC	LC History - Other	P-Value
Molecular Subtype								
Proliferative	14	5	33		12	9	7	
Inflammatory	10	6	21		12	4	14	
Secretory	26	8	27		14	13	7	
Normal-like	9	3	28	p=0.19	6	1	6	p=0.10

Supplementary Table 6. Molecular Subtype associations with previous history of lung cancer. Previous history of lung cancer (LC) was categorized as follows: no history (No LC History), a previous history of LC that include a lung squamous cell carcinoma (LC History – LUSC), and a previous history of LC that does not include a lung squamous cell carcinoma (LC History – Other). Statistical tests within the discovery and validation cohorts were performed using two-sided Fisher's exact tests. Source data are provided as a Source Data file.

			Samples Used For Scoring Each Panel							
IF Panel	All Samples		K5/KI67/Ac-alpha-Tubulin		CD68/CD163		CD4		CD8	
Variable	Discovery Cohort	Validation Cohort	Discovery Cohort	Validation Cohort	Discovery Cohort	Validation Cohort	Discovery Cohort	Validation Cohort	Discovery Cohort	Validation Cohort
Number of Subjects	17	12	7	2	17	12	17	11	17	11
Number of Samples	27	20	8	2	25	18	27	19	26	18
**Subtype**										
Normal-like	2/27 (7)	1/20 (5)	1/8 (13)	0/2 (0)	2/25 (8)	1/18 (6)	2/27 (7)	1/19 (5)	2/26 (8)	1/18 (6)
Secretory	7/27 (26)	5/20 (25)	1/8 (13)	0/2 (0)	7/25 (28)	4/18 (22)	7/27 (26)	5/19 (26)	7/26 (27)	5/18 (28)
Inflammatory	8/27 (30)	3/20 (15)	2/8 (25)	1/2 (50)	7/25 (28)	2/18 (11)	8/27 (30)	3/19 (16)	8/26 (31)	3/18 (17)
Proliferative	10/27 (37)	11/20 (55)	4/8 (50)	1/2 (50)	9/25 (36)	11/18 (61)	10/27 (37)	10/19 (53)	9/26 (35)	9/18 (50)
**Histology**										
Normal/Hyperplasia	9/27 (33)	2/20 (10)	3/8 (38)	0/2 (0)	9/25 (36)	1/18 (6)	9/27 (33)	2/19 (11)	9/26 (35)	2/18 (11)
Squamous Metaplasia	3/27 (11)	3/20 (15)	0/8 (0)	1/2 (50)	3/25 (12)	3/18 (17)	3/27 (11)	3/19 (16)	3/26 (12)	3/18 (17)
Mild Dysplasia	1/27 (4)	4/20 (20)	0/8 (0)	0/2 (0)	1/25 (4)	4/18 (22)	1/27 (4)	4/19 (21)	1/26 (4)	4/18 (22)
Moderate Dysplasia	8/27 (30)	9/20 (45)	3/8 (38)	1/2 (50)	7/25 (28)	8/18 (44)	8/27 (30)	8/19 (42)	7/26 (27)	7/18 (39)
Severe Dysplasia/CIS	6/27 (22)	2/20 (10)	2/8 (25)	0/2 (0)	5/25 (20)	2/18 (11)	6/27 (22)	2/19 (11)	6/26 (23)	2/18 (11)
**Lesion State**										
Progressive/Persistent	7/27 (26)	10/20 (50)	1/8 (13)	1/2 (50)	7/25 (28)	9/18 (50)	7/27 (26)	10/19 (53)	6/26 (23)	9/18 (50)
Regressive	6/27 (22)	5/20 (25)	3/8 (38)	0/2 (0)	5/25 (20)	5/18 (28)	6/27 (22)	4/19 (21)	6/26 (23)	4/18 (22)
nknown or Normal/Stable	14/27 (52)	5/20 (25)	4/8 (50)	1/2 (50)	13/25 (52)	4/18 (22)	14/27 (52)	5/19 (26)	14/26 (54)	5/18 (28)
**Smoking Status**										
Current	15/27 (56)	12/20 (60)	6/8 (75)	1/2 (50)	13/25 (52)	12/18 (67)	15/27 (56)	11/19 (56)	14/26 (54)	10/18 (56)
Former/Never	12/27 (44)	8/20 (40)	2/8 (25)	1/2 (50)	12/25 (48)	6/18 (33)	12/27 (44)	8/19 (42)	12/26 (46)	8/18 (44)

Supplementary Table 7. Clinical and biological characteristics of the samples used for immunofluorescence studies. For each characteristic, percentages are reported in parenthesis.

**Molecular Subtype**	Normal	Normal	Secretory	Secretory	Inflammatory	Inflammatory	Proliferative	Proliferative
**Cohort**	DC	VC	DC	VC	DC	VC	DC	VC
**Number of Progressive/Persistent Lesions**	5	1	17	7	7	5	15	7
**Number of Regressive Lesions**	3	3	8	1	4	1	15	13
**Module Number**								
1	ns	N/A	ns	N/A	ns	N/A	ns	ns
2	ns	N/A	ns	N/A	ns	N/A	ns	ns
3	ns	N/A	ns	N/A	ns	N/A	0.047	ns
4	0.026	N/A	ns	N/A	ns	N/A	ns	ns
5	ns	N/A	ns	N/A	ns	N/A	ns	ns
6	ns	N/A	ns	N/A	ns	N/A	ns	ns
7	ns	N/A	ns	N/A	ns	N/A	ns	ns
8	0.027	N/A	ns	N/A	0.005	N/A	ns	ns
9	ns	N/A	ns	N/A	ns	N/A	0.0017	0.03

Supplementary Table 8. Statistical associations between progression/persistence versus regression within each molecular subtype and cohort for each gene module. The P-values were calculated based on a linear model (implemented in limma) with GSVA scores for each module as the dependent variable and progression/regression status as the independent variable and patient as a random effect. P-values less than 0.05 are reported. ns= not significant and N/A= not enough samples in each group to conduct the analysis. DC = Discovery Cohort and VC = Validation Cohort. Source data are provided as a Source Data file.

ID	Name	Description
096	VC	True Vocal Cords, Neck
051	Mouth	Floor of Mouth
007	EPIG	Epiglottis
005	ART	Arytenoids
008	FVC	False Vocal Cords
095	TR	Trachea
050	MC	Main Carina, Carina NOS
086	RMB	Right Main Bronchus, incl Secondary Carina right
091	RUL	Right Upper Lobe
093	RULO	Right Upper Lobe Orifice or opening
094	RULS	Right Upper Lobe Stump
092	RULB	Right Upper Lobe Bronchus
087	RML	Right Middle Lobe
089	RMLO	Right Middle Lobe Orifice or opening
090	RMLS	Right Middle Lobe Stump
088	RMLB	Right Middle Lobe Bronchus
082	RLL	Right Lower Lobe
084	RLLO	Right Lower Lobe Orifice
085	RLLS	Right Lower Lobe Stump
083	RLLB	Right Lower Lobe Bronchus
006	BI	Bronchus Intermedius
052	RB1	RUL Apical Segment (AS)
060	RB2	RUL Posterior Segment (PS)
063	RB3	RUL Anterior Segment (ANTS)
053	RB1/2	RUL Carina between RB1 and RB2
054	RB1/3	RUL Carina between RB1 and RB3
061	RB2/3	RUL Carina between RB2 and RB3
059	RB1A/B	RUL AS Carina between RB1 A and B
062	RB2A/B	RUL PS Carina between RB2 A and B
064	RB3A/B	RUL ANTS Carina between RB3 A and B
065	RB4	RML Lateral Segment (LS)
068	RB5	RML Medial Segment (MS)
066	RB4/5	RML LS Carina between RB4 and RB5
067	RB4A/B	RML LS Carina between RB4 A and B
069	RB5A/B	RML MS Carina between RB5 A and B
070	RB6	RLL Superior Basal Segment (SBS)
071	RB6A/B	RLL SBS Carina between RB6A and B
072	RB6A/C	RLL SBS Carina between RB6A and C
073	RB6B/C	RLL SBS Carina between RB6B and C
074	RB7	RLL Medial Basal Segment (MBS)
075	RB7A/B	RLL MBS Carina between RB7A and B
076	RB8	RLL Anterior Basal Seg (ABS)
077	RB8/9	RLL ABS Carina between RB8 and RB9
078	RB8A/B	RLL ABS Carina between RB8A and B
079	RB9	RLL Lateral Basal Segment (LBS)
080	RB9/10	RLL LBS Carina between RB9 and RB10
081	RB9A/B	RLL LBS Carina between RB9A and B
055	RB10	RLL Posterior Basal Segment (PBS)
056	RB10A/B	RLL PBS Carina between RB10A and B
057	RB10A/C	RLL PBS Carina between RB10A and C
058	RB10B/C	RLL PBS Carina between RB10B and C
001	666	Location was surgically altered or removed
002	777	Abstractor needs clinician help to code
003	888	Location code is unknown, illegible
004	999	Location code is blank, not noted
043	LMB	Left Main Bronchus, incl Secondary Carina left
044	LMBD	Left Main Bronchus, Distal
046	LUL	Left Upper Lobe
048	LULO	Left Upper Lobe Orifice or opening
049	LULS	Left Upper Lobe Stump
035	LGL	Lingula
037	LGLO	Lingula Orifice or opening
038	LGLS	Lingula Stump
047	LULB	Left Upper Lobe Bronchus
045	LUDB	Left Upper Division Bronchus
036	LGLDB	Lingular Division Bronchus, lingular bronchus
039	LLL	Left Lower Lobe
041	LLLO	Left Lower Lobe Orifice or opening
042	LLLS	Left Lower Lobe Stump
040	LLLB	Left Lower Lobe Bronchus
009	LB1+2	LUL Apical-Posterior Segment (APS)
018	LB3	LUL Anterior Segment
011	LB1/2	LUL APS Carina between LB1 and LB2
010	LB1+2/3	LUL APS Carina between LB1+2 and LB3
016	LB2A/C	LUL APS Carina between LB2 A and C
017	LB2B/C	LUL APS Carina between LB2B and C
019	LB3A/B	LUL ANTS Carina between LB3A and B
020	LB4	LUL Superior Lingular Segment (SLS)
023	LB5	LUL Inferior Lingular Segment (ILS)
021	LB4/5	LUL SLS Carina between LB4 and LB5
022	LB4A/B	LUL SLS Carina between LB4A and B
024	LB5A/B	LUL ILS Carina between LB5A and B
025	LB6	LLL Superior Segment (SS)
026	LB6A/B	LLL SS Carina between LB6A and B
027	LB6A/C	LLL SS Carina between LB6A and C
028	LB6B/C	LLL SS Carina between LB6B and C
029	LB8	LLL Antero Medial Basal Segment (AMBS)
030	LB8/9	LLL AMBS Carina between LB8 and LB9
031	LB8A/B	LLL AMBS Carina between LB8A and B
032	LB9	LLL Lateral Basal Segment (LBS)
033	LB9/10	LLL LBS Carina between LB9 and LB10
034	LB9A/B	LLL LBS Carina between LB9A and B
012	LB10	LLL Posterior Basal Segment (PBS)
013	LB10A/B	LLL PBS Carina between LB10A and B
014	LB10A/C	LLL PBS Carina between LB10A and C
015	LB10B/C	LLL PBS Carina between LB10B and C

Supplementary Table 9. Lung sites where endobronchial biopsies were obtained. The site code, name, and description are reported for each site.

Antibody	Company	Catalog	Dilution	Antigen retrieval	Species
**Immune cell type markers**					
CD68	Dako	m0876	1- 250	AR6	mouse
CD163	Cell Marque	163m-16	1-100	AR9	mouse
CD4	Thermo Fisher	ms1528S	1-100	AR9	mouse
CD8	Dako	M7103	1-100	AR9	mouse
**Epithelial cell type and proliferation markers**					
Ac-α-Tub	Sigma	T6793	1-100	citrate	mouse
KRT5	BioLegend	905-901	1-100	citrate	chicken
KI67	Abcam	ab16667	1-100	citrate	rabbit

Supplementary Table 10. Antibodies used in the immunofluorescence studies.

Supplementary Figures

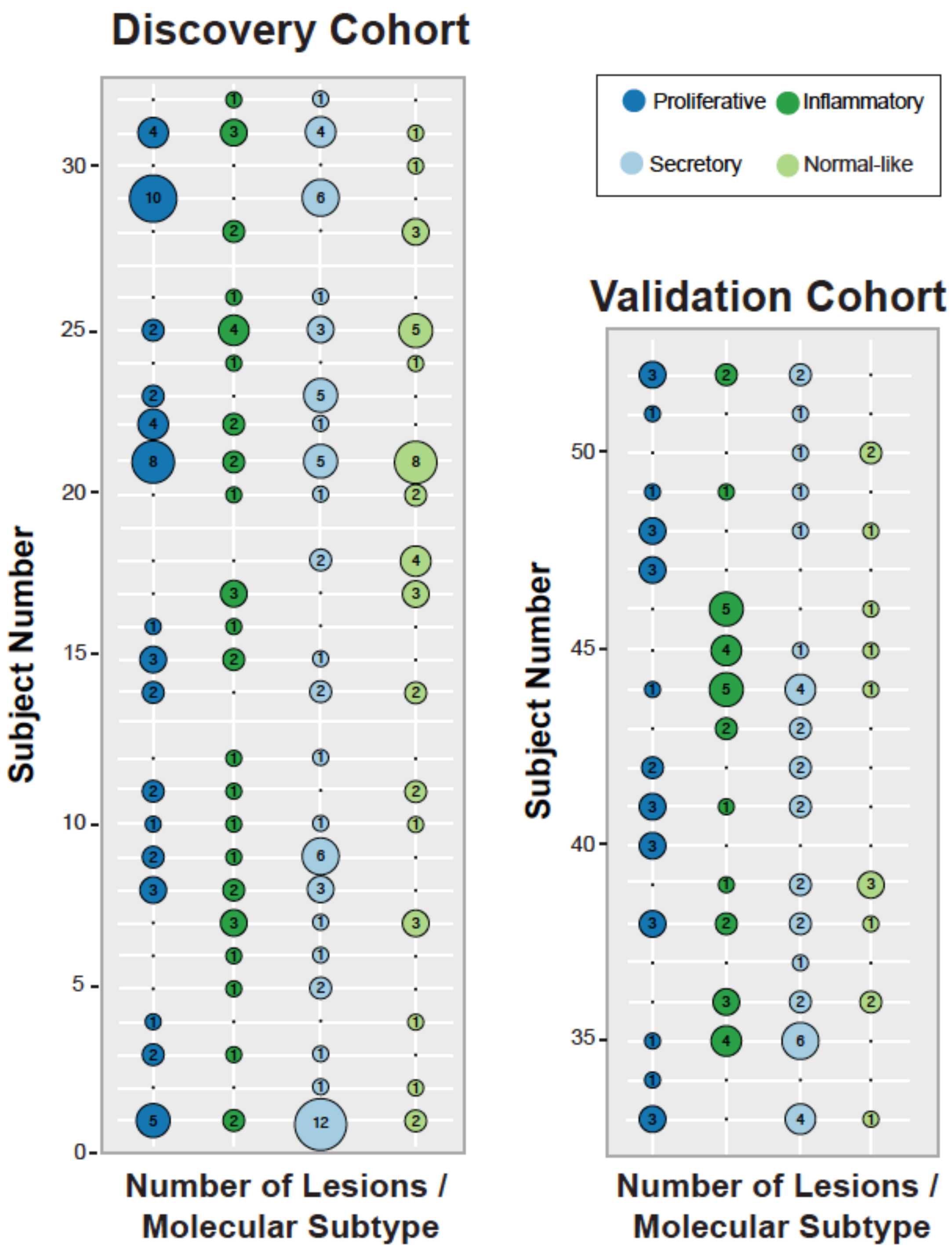

Supplementary Figure 1. Distribution of molecular subtypes by subject across the biopsies. The columns represent the 4 molecular subtypes (Proliferative, dark blue; Inflammatory, dark green; Secretory, light blue; and Normal-like, light green) and the radius of the circle is proportional to the number of samples within each subtype. The discovery cohort samples are shown on the right and the validation cohort samples are shown on the left. Source data are provided as a Source Data file.

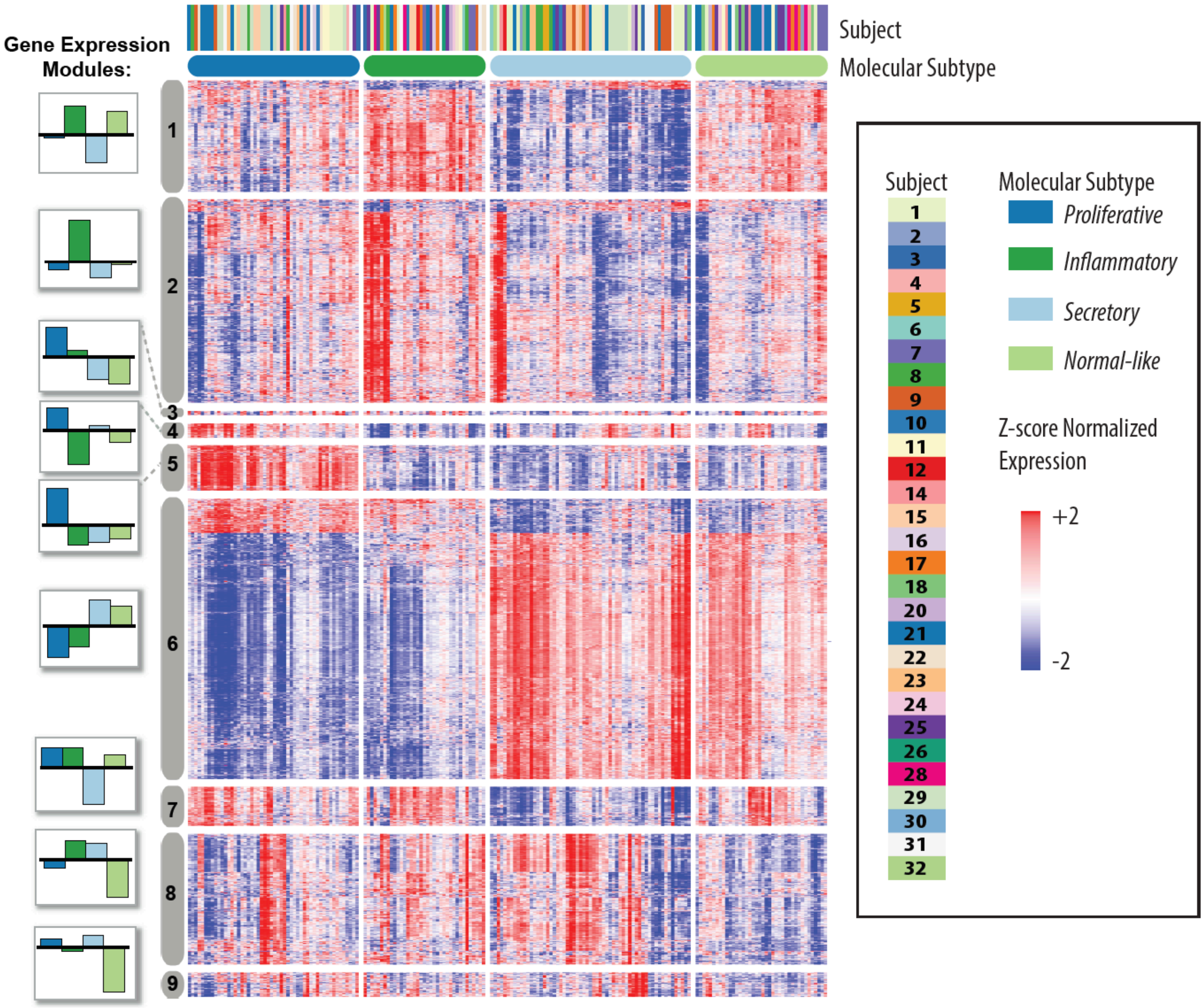

Supplementary Figure 2. Distribution of subject among the discovery cohort endobronchial biopsies across the four molecular subtypes. Genes (n=3,936) organized into 9 gene co-expression modules were used to discover four molecular subtypes (Proliferative, Inflammatory, Secretory, and Normal-like) across the 190 discovery cohort (DC) biopsies using consensus clustering. The heatmap shows semi-supervised hierarchal clustering of z-score normalized gene expression across the 3,936 genes and 190 DC biopsies. The top color bars represent the subject the sample was derived and molecular subtype membership: Proliferative (n=52 samples), Inflammatory (n=37 samples), Secretory (n=61 samples), and Normal-like (n=40 samples). On the left side of the heatmap, the mean module GSVA score is plotted for each subtype. Source data are provided as a Source Data file.

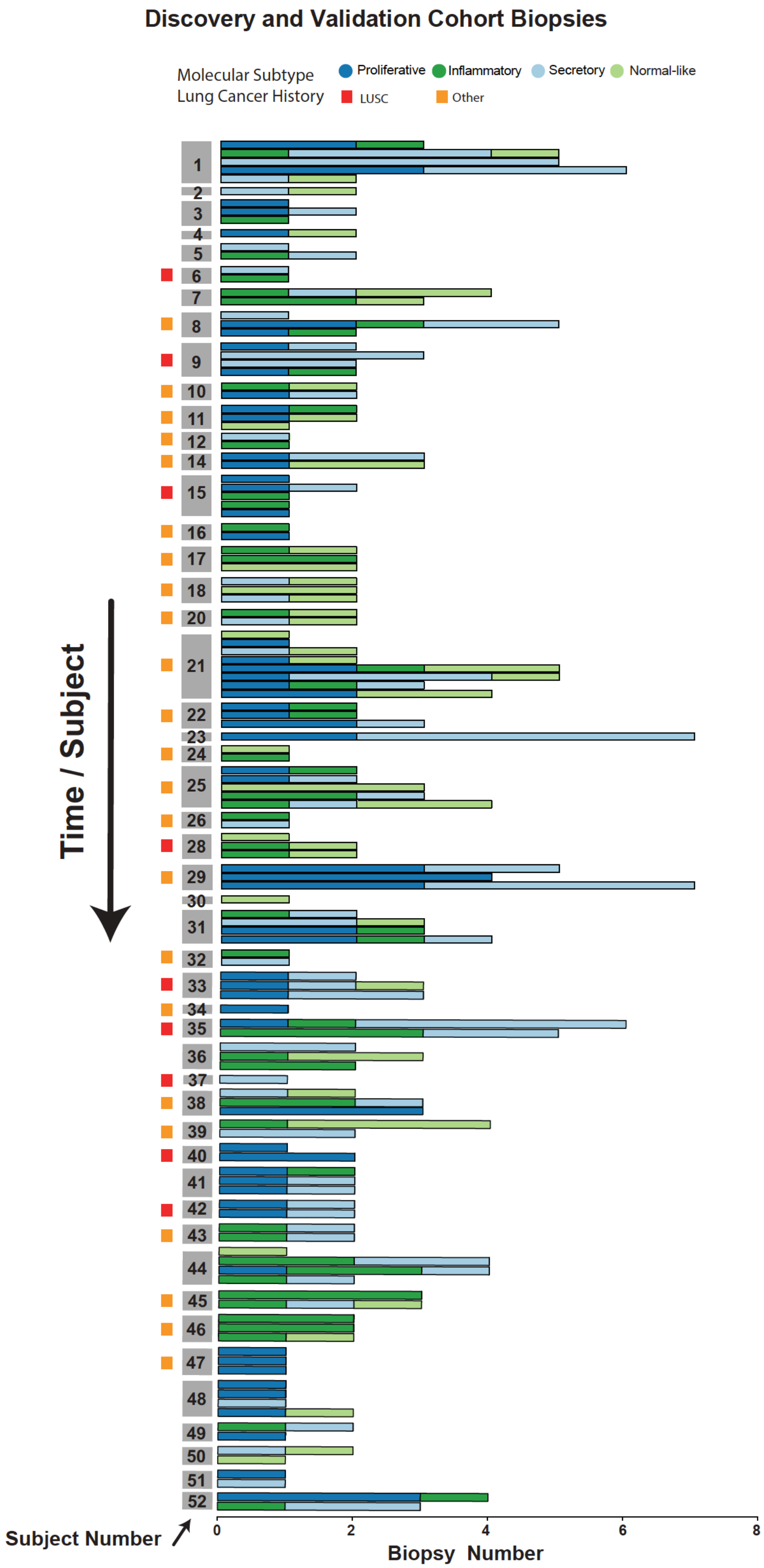

Supplementary Figure 3. Molecular subtype distribution for each subject across bronchoscopy procedures. The barplot shows for each subject and each bronchoscopy procedure the number of biopsies sampled and their corresponding molecular subtype. The y-axis indicates the subject number and whether or not that subject had a prior history of either lung squamous cell carcinoma (LUSC, red) or another type of lung cancer (Other, yellow). The discovery cohort includes subjects 1 through 32 and the validation cohort includes subjects 33 through 52. We did not detect a difference in the diversity of subtype classifications within a subject based on prior history of lung cancer (mean Shannon entropy of subtype classifications within patients with a history of lung cancer = 1.12, n=32 vs. patients without a history of lung cancer = 1.25, n = 17; Wilcoxon Rank Sum test p-value = 0.43). Source data are provided as a Source Data file.

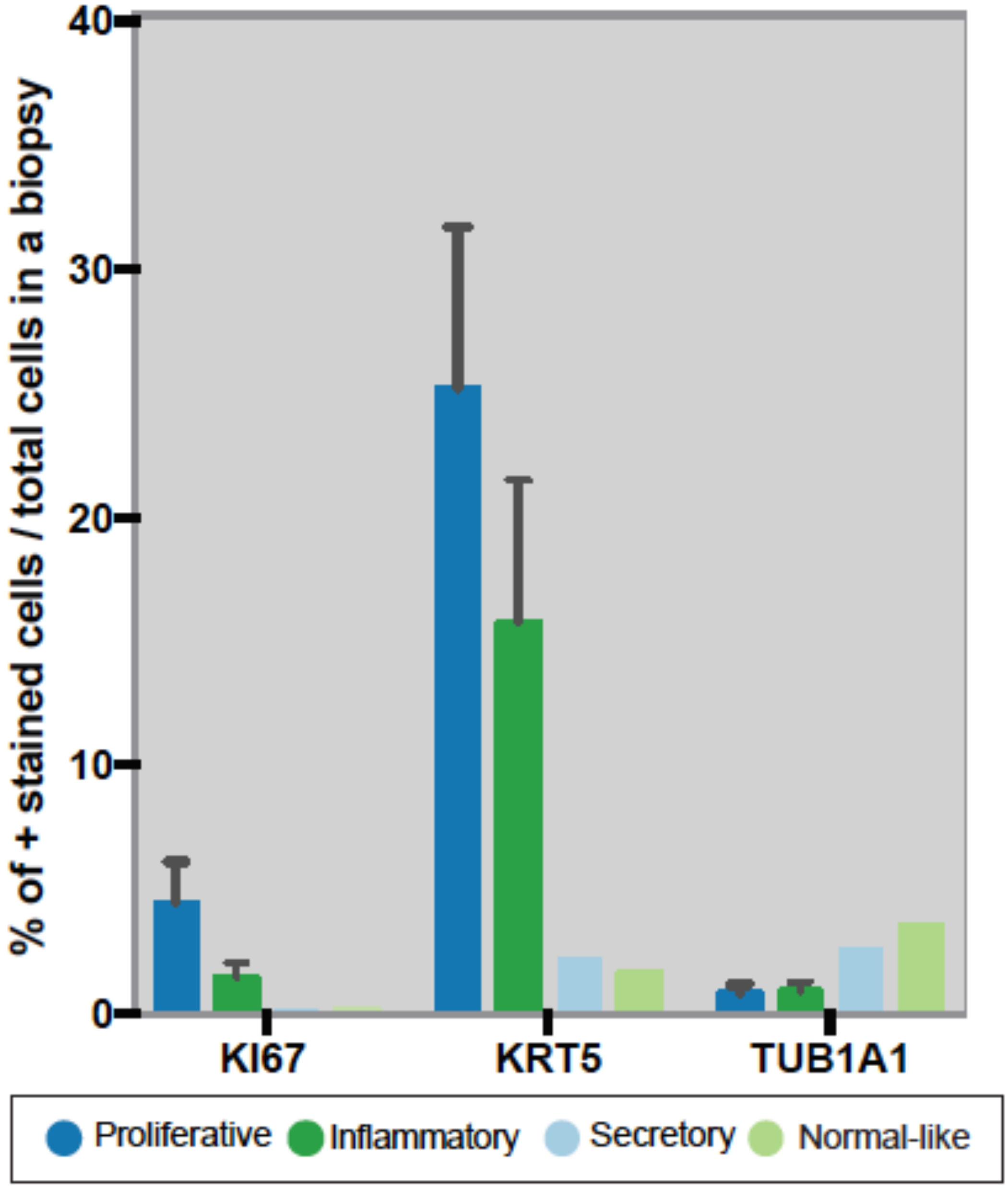

Supplementary Figure 4. Immunofluorescent staining quantitation of proliferation, basal cell, and ciliated cell markers across the molecular subtypes. Boxplot of immunofluorescent staining quantitation of KI67 (proliferation), KRT5 (basal cell) and TUB1A1 (ciliated cell) across representative samples from each molecular subtype (Proliferative n= 4, Inflammatory n=3, Secretory n=1, Normal-like n=1). KI67 and KRT5 staining are significantly higher in samples in the Proliferative subtype (p=0.02 and p=0.01 via linear models, respectively, for sample differences between the Proliferative subtype and other subtypes). TUB1A1 was lower in samples in the Proliferative and Inflammatory subtypes but did not reach statistical significance (p=0.06, linear model, for sample differences between Proliferative and Inflammatory subtypes versus Inflammatory and Secretory subtypes). The error bars represent the standard deviation. Source data are provided as a Source Data file.

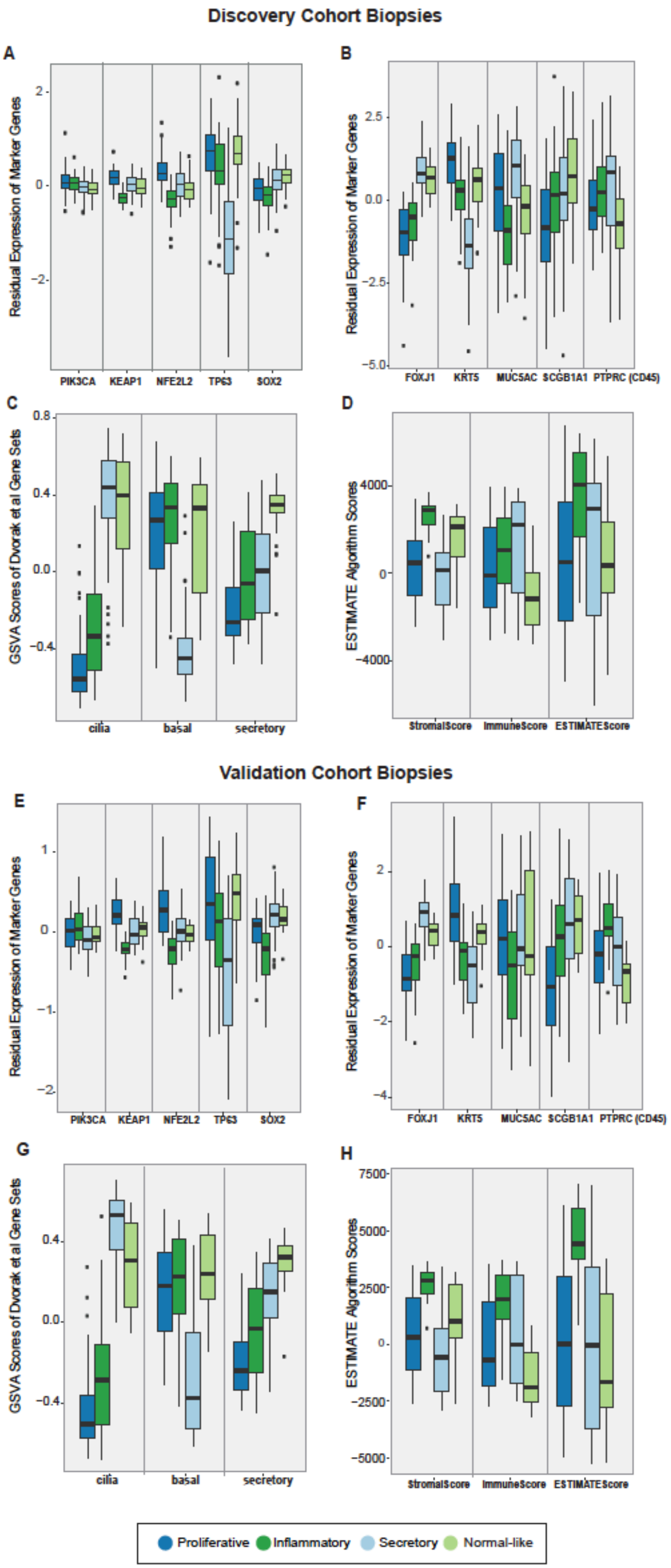

Supplementary Figure 5. Boxplots of select genes and cell type deconvolution results across the discovery and validation cohorts by molecular subtype. (**A-D**) Discovery cohort biopsies. (**E-H**) Validation cohort biopsies. (**A**) and (**E**) show boxplots of gene expression levels of LUSC driver genes identified by TCGA across the molecular subtypes. (**B**) and (**F**) show boxplots of gene expression levels of cell type marker genes across the molecular subtypes. (**C**) and (**G**) show boxplots of GSVA scores calculated using Dvorak *et al*. gene sets across the molecular subtypes. (**D**) and (**H**) show boxplots of ESTIMATE algorithm scores across the molecular subtypes. The ESTIMATE algorithm estimates the stromal (StromalScore), immune (ImmuneScore), and epithelial (ESTIMATEScore) cell fractions in each sample. High immune and stromal scores indicate a high fraction of stromal and immune cells while low epithelial scores indicate a high fraction of epithelial cells. In the boxplots, the upper and lower hinges correspond to the first and third quartile, center line represents the median, and whiskers extend from the hinge to the largest or smallest value at most 1.5 times the distance between the quartiles. Source data are provided as a Source Data file.

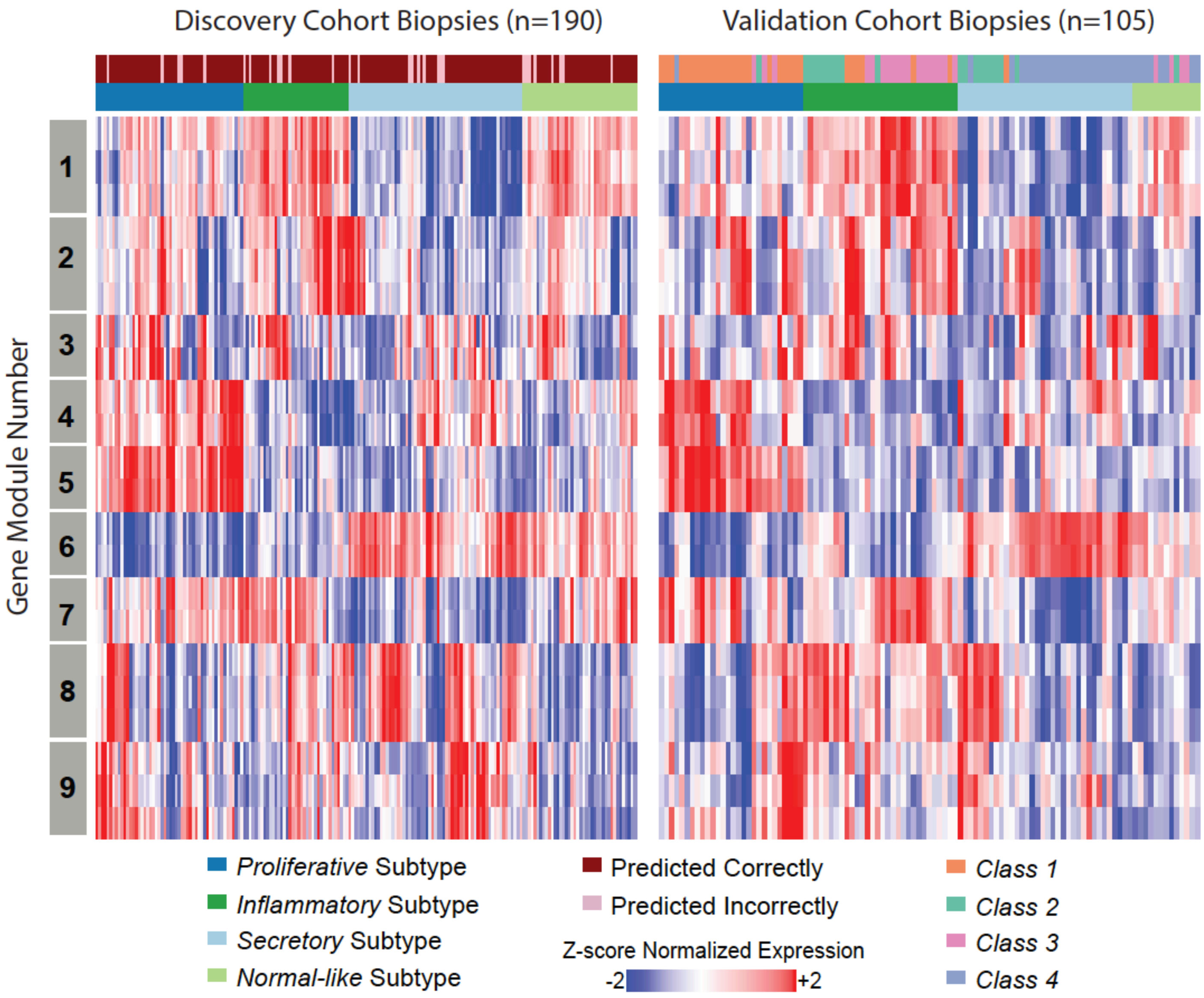

Supplementary Figure 6. Heatmap of the 22-gene molecular subtype classifier in the discovery and validation cohort biopsies. Semi-supervised hierarchal clustering of z-score normalized residual gene expression across the 22 classifier genes and 190 discovery cohort biopsies training samples (left) and the 105 Validation cohort biopsies (right). The rows of the heatmap show the gene module membership. The first column color bar shows molecular subtype membership in the discovery cohort and the 22-gene predicted molecular subtype membership in the validation cohort. The second column color bar depicts correct (dark red) and incorrect (pink) predictions in the discovery cohort using the 22-gene classifier and molecular subtypes (orange, Class 1; turquoise, Class 2; pink, Class 3; and purple/blue, Class 4) derived by performing consensus clustering across the validation cohort using n=3,936 genes. Source data are provided as a Source Data file.

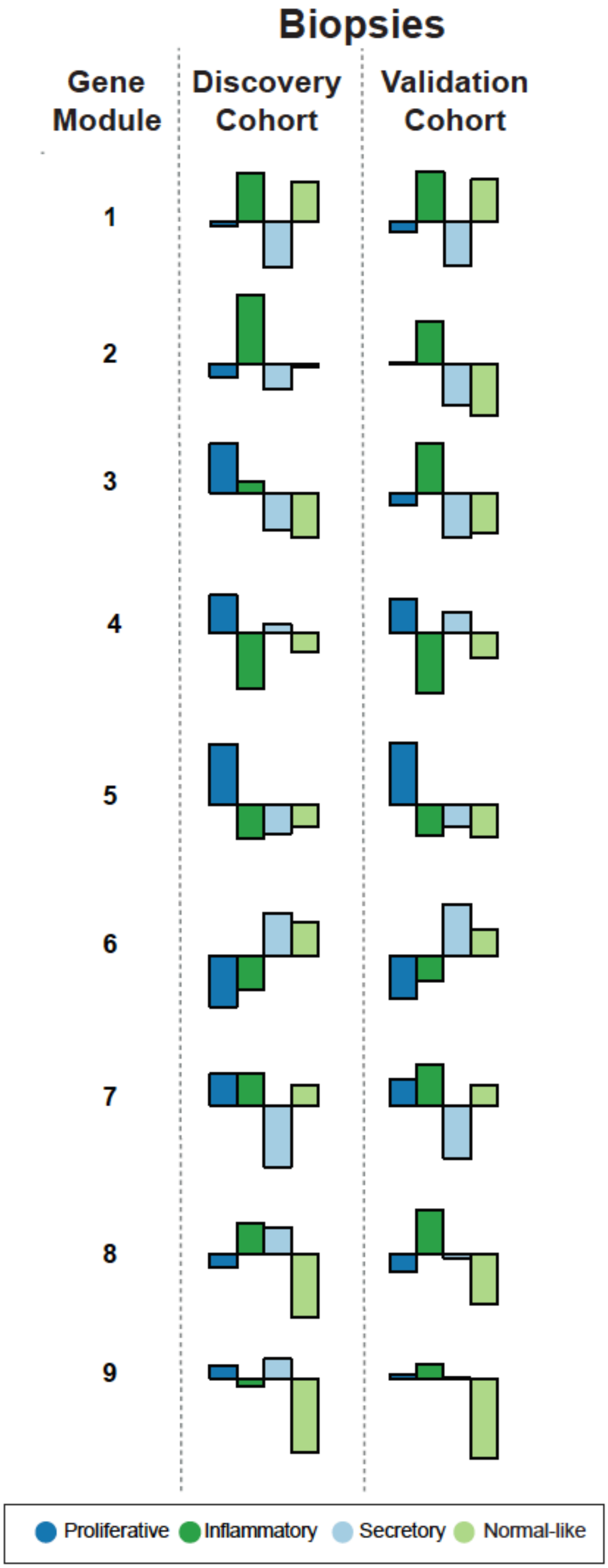

Supplementary Figure 7. Gene module behavior across the molecular subtypes in the discovery and validation cohort biopsies. The mean GSVA score for each module is plotted for each molecular subtype. Source data are provided as a Source Data file.

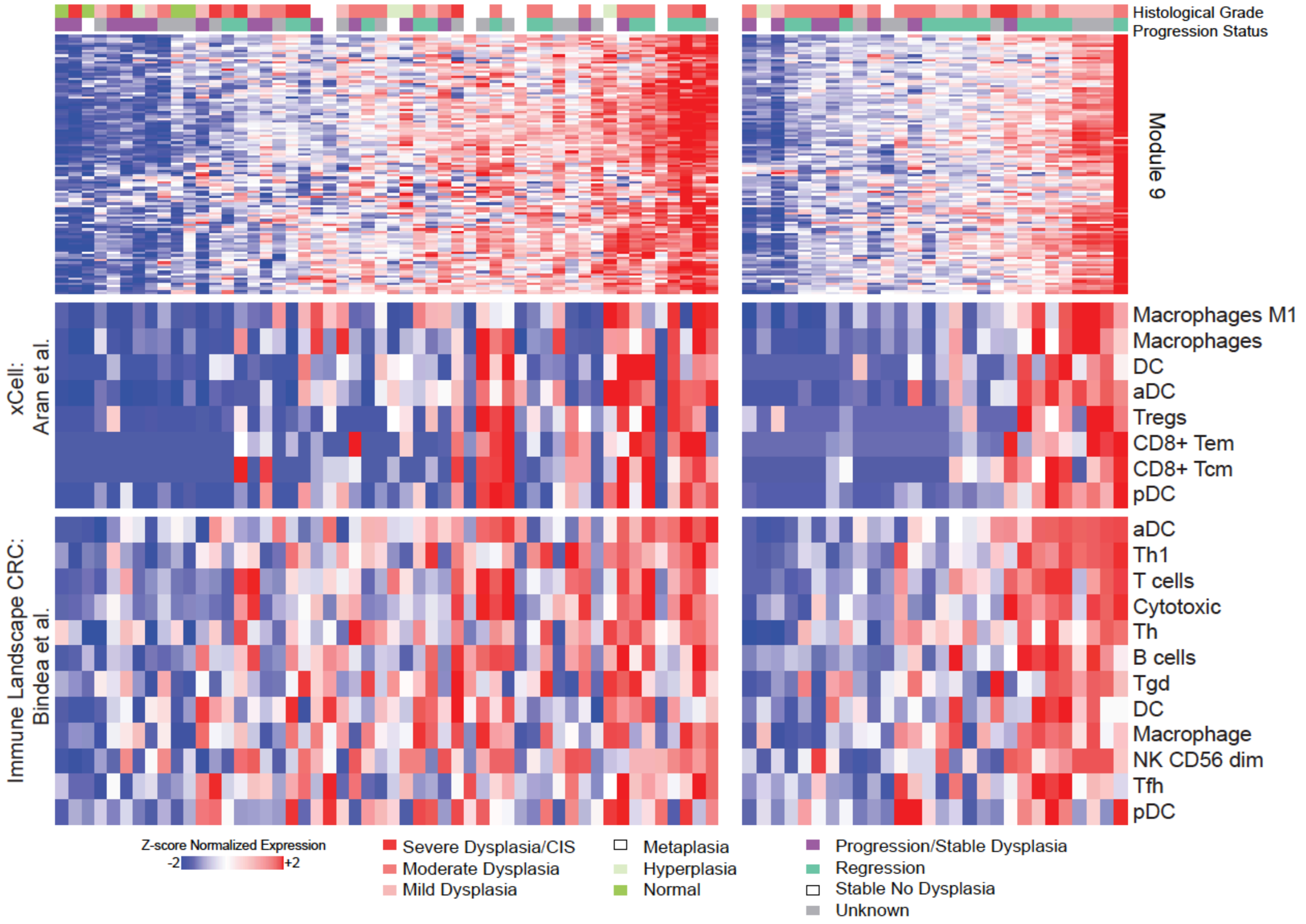

Supplementary Figure 8. Concordance between module 9 and two cell type deconvolution analyses. Top: Hierarchal clustering of z-score normalized gene expression across the 112 genes in module 9 and the Discovery cohort biopsies (left) and the Validation cohort biopsies (right). Each heatmap is supervised according to the module 9 GSVA scores. Top color bars indicate the histological grade of the biopsies and their progression status. xCell results (Middle) and GSVA scores for gene sets described by Bindea et al. (Bottom) indicating the relative abundance of immune cell types across the discovery cohort biopsies (left) and the validation cohort biopsies (right). Immune cell types displayed are significantly associated with lesion progression/persistence (FDR<0.05, linear model, in both the discovery cohort and validation cohort). Source data are provided as a Source Data file.

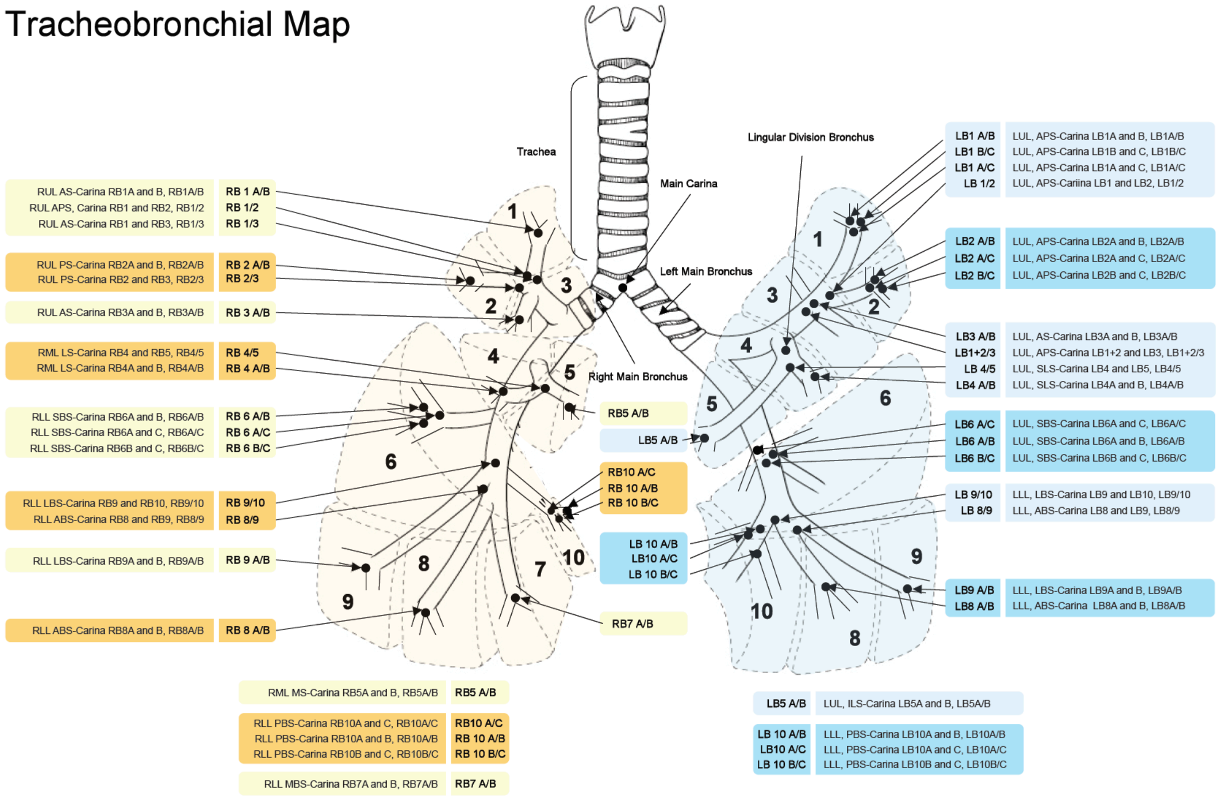

Supplementary Figure 9. Tracheobronchial map. The locations of the sites sampled by endobronchial biopsy.

Supplementary References

1. Dobin, A. *et al.* STAR: ultrafast universal RNA-seq aligner. *Bioinformatics* **29,** 15–21 (2013).
2. Li, B. & Dewey, C. N. RSEM: accurate transcript quantification from RNA-Seq data with or without a reference genome. *BMC Bioinformatics* **12,** 323 (2011).
3. Wang, L., Wang, S. & Li, W. RSeQC: quality control of RNA-seq experiments. *Bioinformatics* **28,** 2184–2185 (2012).
4. Robinson, M. D., McCarthy, D. J. & Smyth, G. K. edgeR: a Bioconductor package for differential expression analysis of digital gene expression data. *Bioinformatics* **26,** 139–140 (2010).
5. Campbell, J. D. *et al.* Distinct patterns of somatic genome alterations in lung adenocarcinomas and squamous cell carcinomas. *Nature Publishing Group* **48,** 607–616 (2016).
6. Beane, J. *et al.* Reversible and permanent effects of tobacco smoke exposure on airway epithelial gene expression. *Genome Biol.* **8,** R201 (2007).
