# Supplementary material for: Molecular subtyping reveals immune alterations associated with progression of bronchial premalignant lesions
Source: Nat Commun. 2019 Apr 23;10:1856. doi: 10.1038/s41467-019-09834-2 (PMC6478943; doi:10.1038/s41467-019-09834-2)
Supplement: Supplementary file 2 — Description of Additional Supplementary Files [file 41467_2019_9834_MOESM2_ESM.docx]

**Title:** Supplementary Data 1
**Description:** Biological pathways significantly enriched in each of the gene modules. Biological processes and pathways enriched in each of the nine modules used to discover the molecular subtypes in the discovery cohort were identified using EnrichR. Each module was separated into genes positively or negatively correlated with the module eigengene and the Ensembl IDs were converted to gene symbols using biomaRt, and the following databases were queried: GO Biological Process 2015, KEGG 2016, WikiPathways 2016, TargetScan microRNA, Transcription Factor PPIs, TRANSFAC and JASPAR PWMs, OMIM Disease, Reactome 2016, and Biocarta 2016. Processes/pathways with an FDR
