# Supplementary figures and images for: Molecular subtyping reveals immune alterations associated with progression of bronchial premalignant lesions
Source: Nat Commun. 2019 Apr 23;10:1856. doi: 10.1038/s41467-019-09834-2 (PMC6478943; doi:10.1038/s41467-019-09834-2)

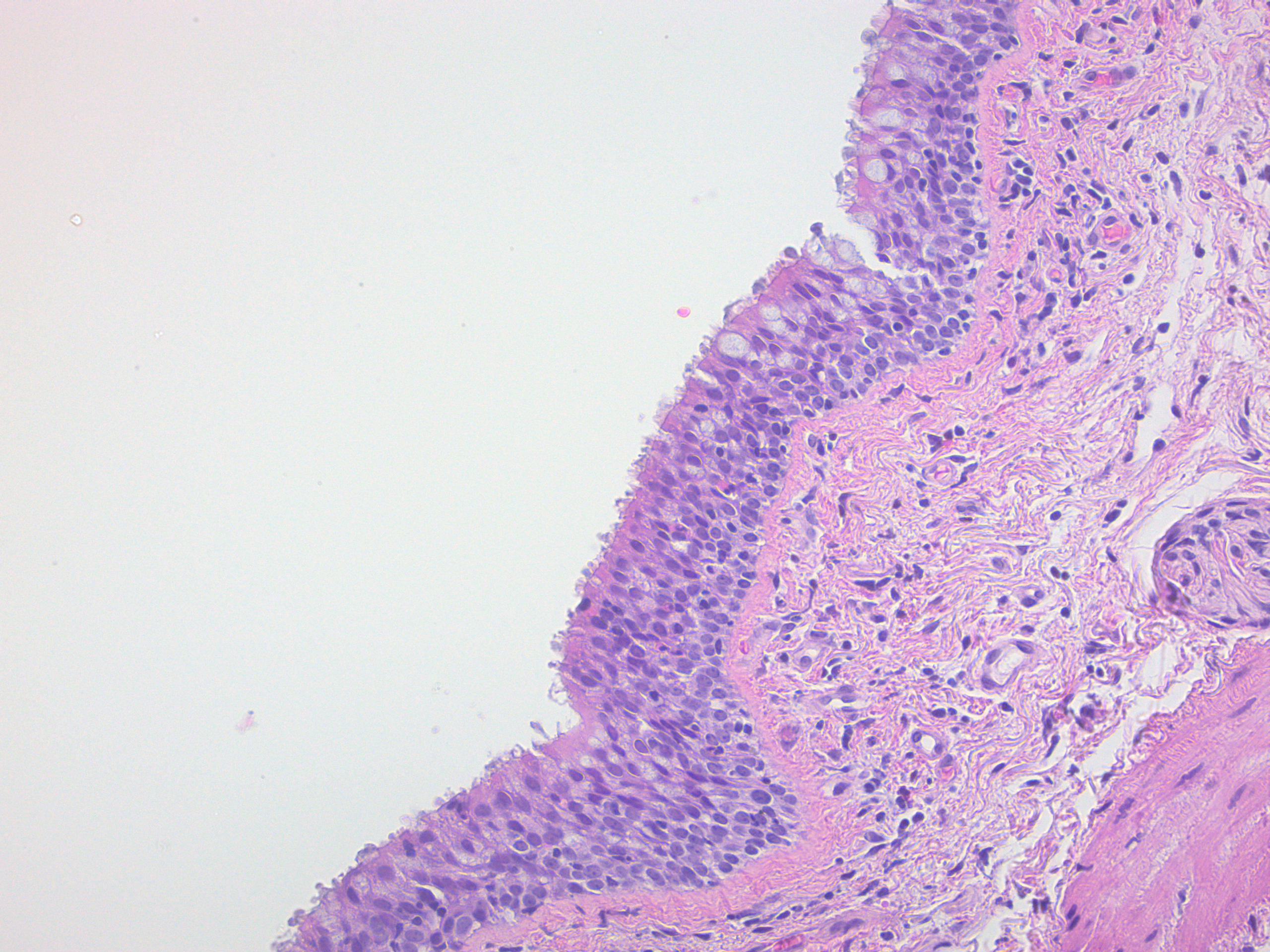

Supplement: Supplementary file 5 — Raw Image File 2 for Figure 1E [file 41467_2019_9834_MOESM5_ESM.tif]

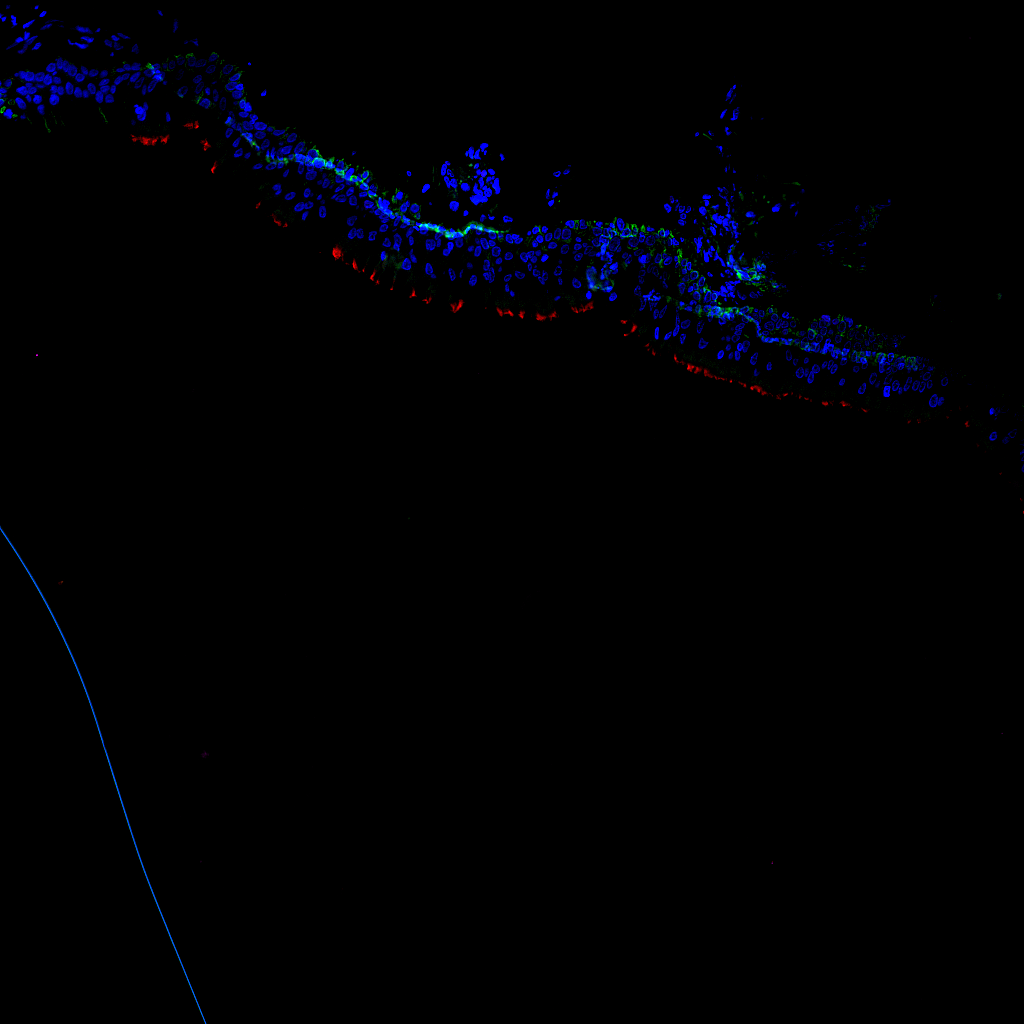

Supplement: Supplementary file 7 — Raw Image File 4 for Figure 1E [file 41467_2019_9834_MOESM7_ESM.tif]

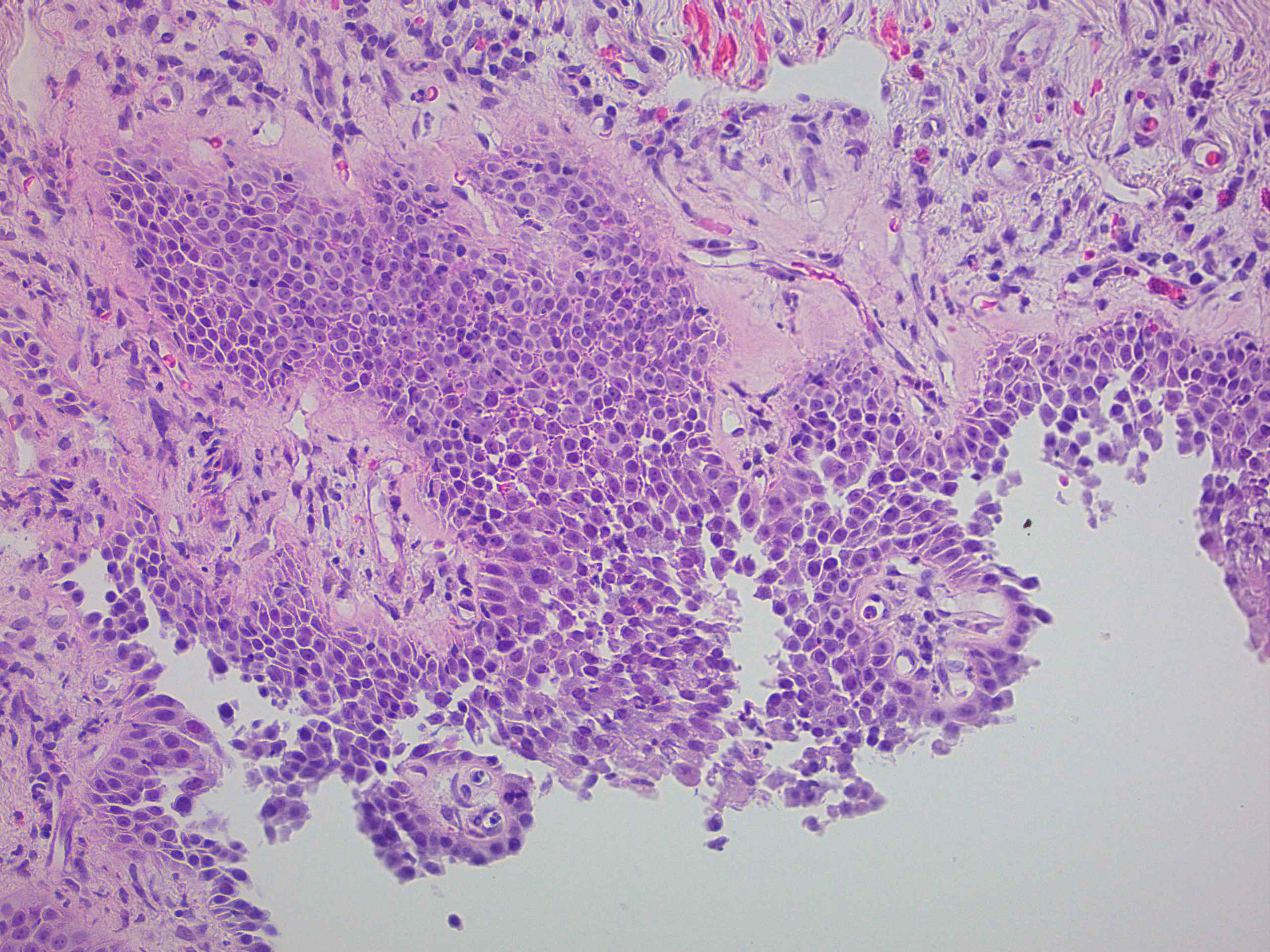

Supplement: Supplementary file 9 — Raw Image File 6 for Figure 1E [file 41467_2019_9834_MOESM9_ESM.tif]

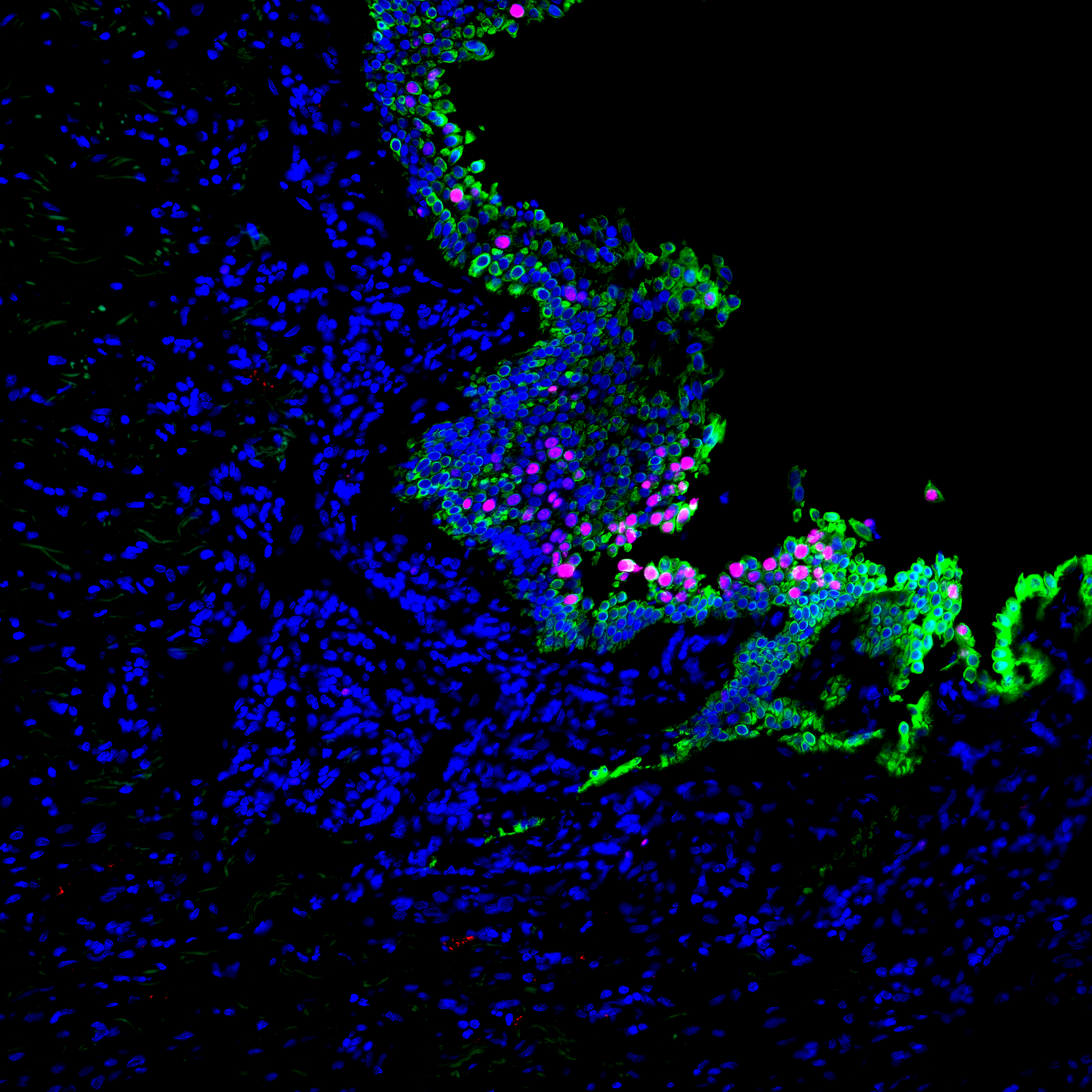

Supplement: Supplementary file 11 — Raw Image File 8 for Figure 1E [file 41467_2019_9834_MOESM11_ESM.tif]

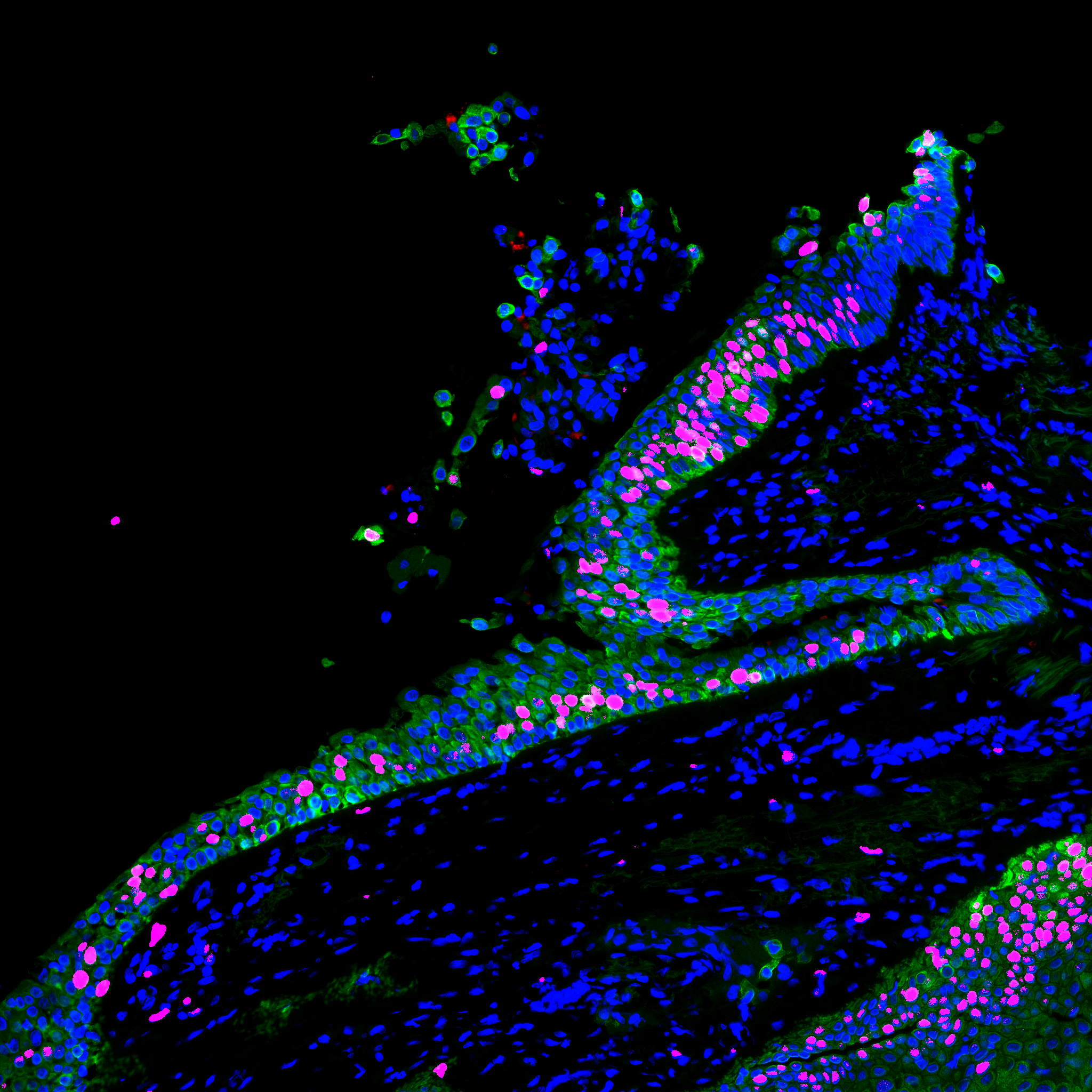

Supplement: Supplementary file 13 — Raw Image File 10 for Figure 1E [file 41467_2019_9834_MOESM13_ESM.tif]

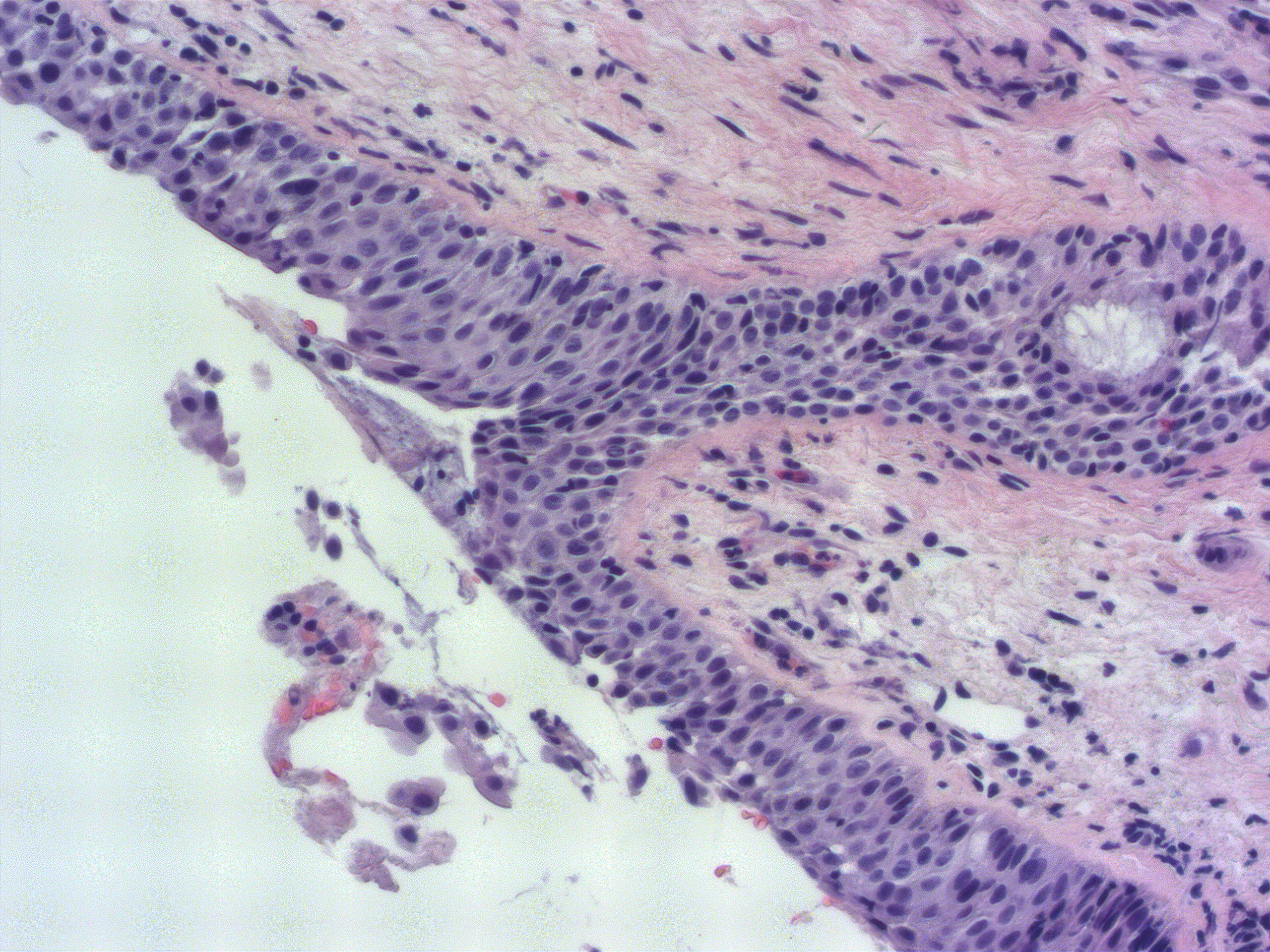

Supplement: Supplementary file 14 — Raw Image File 11 for Figure 1E [file 41467_2019_9834_MOESM14_ESM.tif]

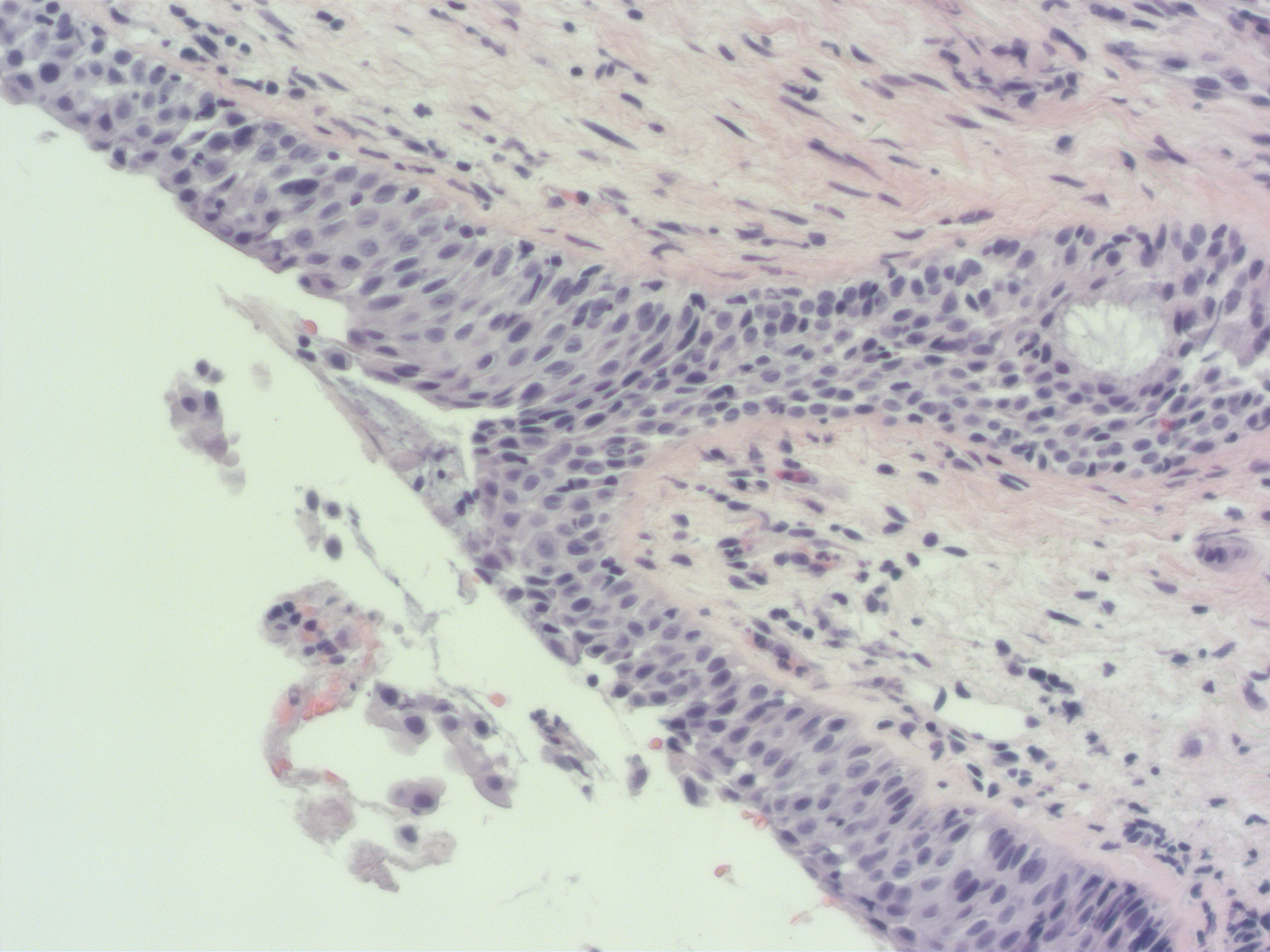

Supplement: Supplementary file 15 — Raw Image File 12 for Figure 1E [file 41467_2019_9834_MOESM15_ESM.tif]

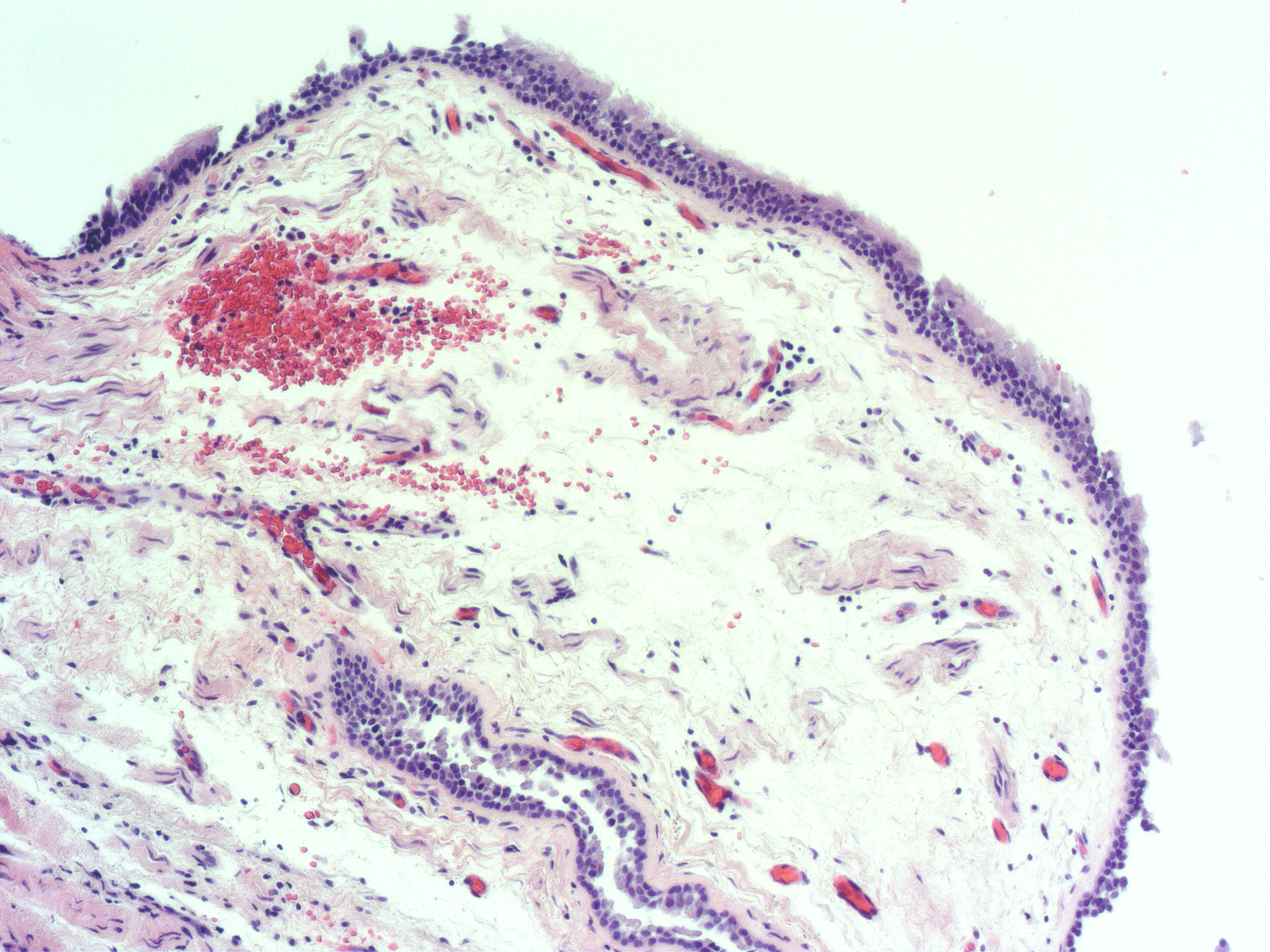

Supplement: Supplementary file 16 — Raw Image File 13 for Figure 1E [file 41467_2019_9834_MOESM16_ESM.tif]

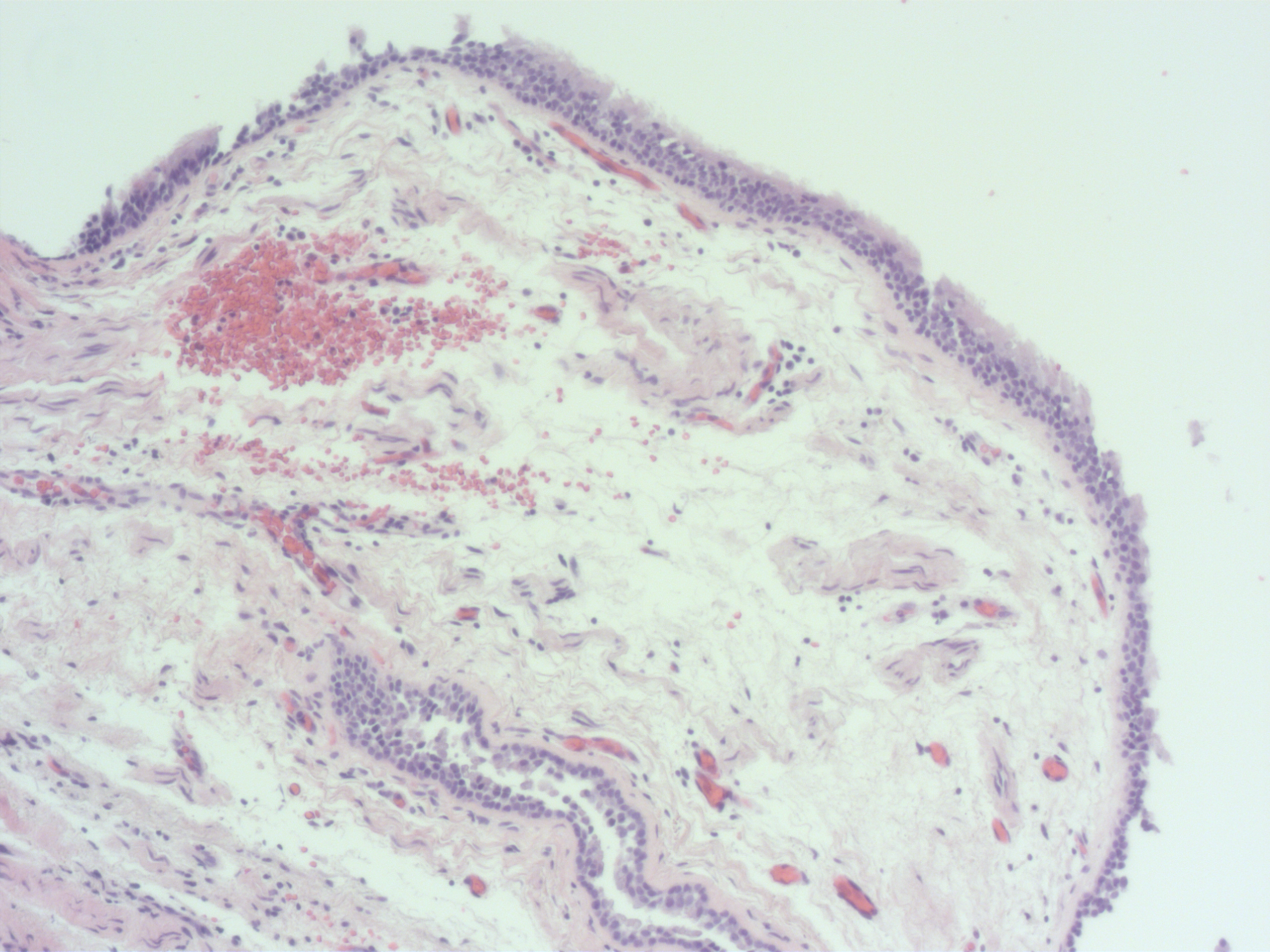

Supplement: Supplementary file 17 — Raw Image File 14 for Figure 1E [file 41467_2019_9834_MOESM17_ESM.tif]

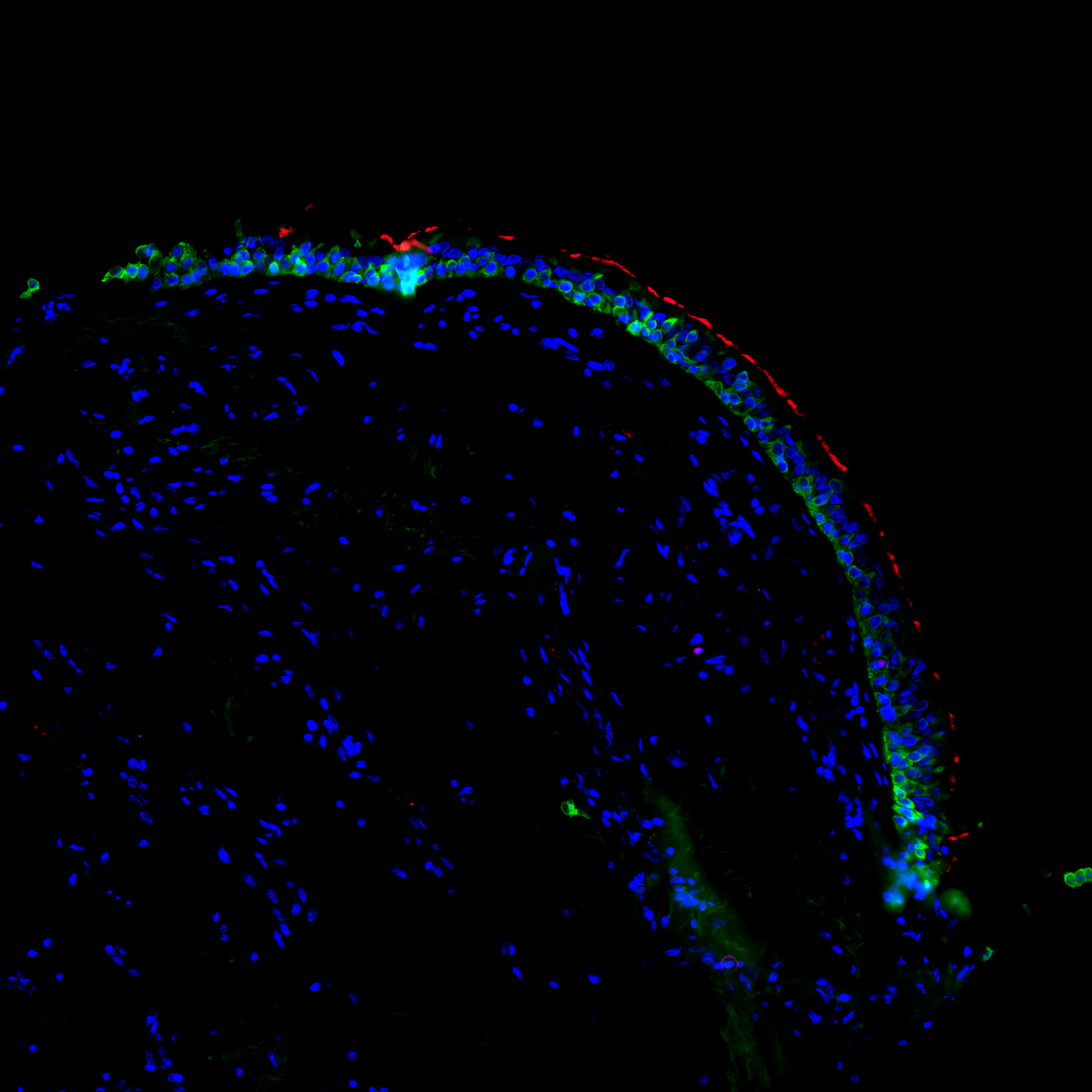

Supplement: Supplementary file 18 — Raw Image File 15 for Figure 1E [file 41467_2019_9834_MOESM18_ESM.tif]

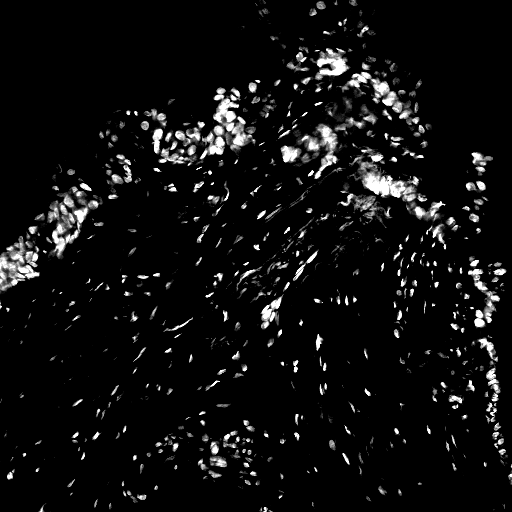

Supplement: Supplementary file 20 — Raw Image File 17 for Figure 4D [file 41467_2019_9834_MOESM20_ESM.tif]

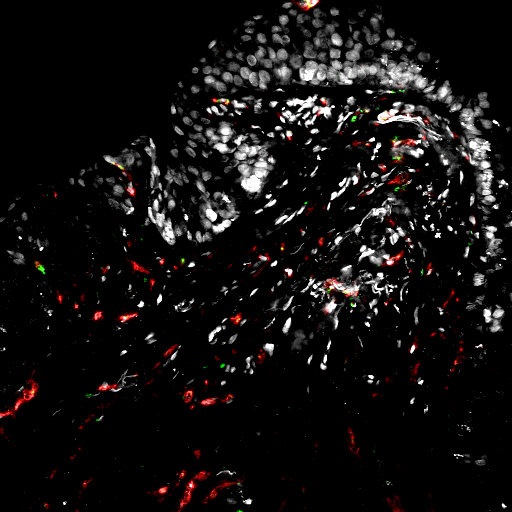

Supplement: Supplementary file 22 — Raw Image File 19 for Figure 4D [file 41467_2019_9834_MOESM22_ESM.tif]

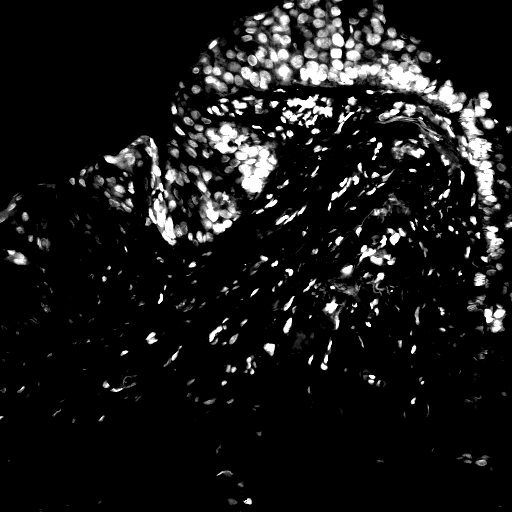

Supplement: Supplementary file 23 — Raw Image File 20 for Figure 4D [file 41467_2019_9834_MOESM23_ESM.tif]

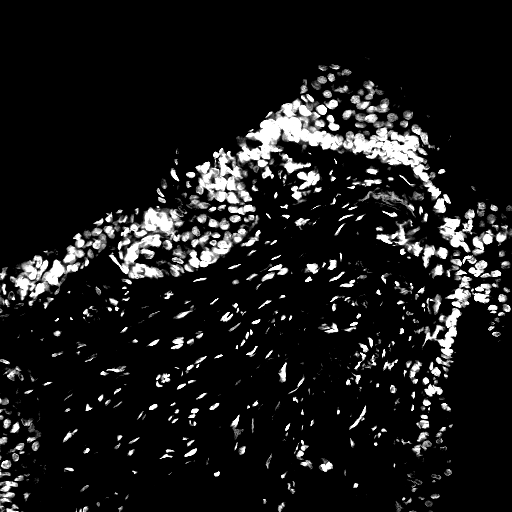

Supplement: Supplementary file 24 — Raw Image File 21 for Figure 4D [file 41467_2019_9834_MOESM24_ESM.tif]

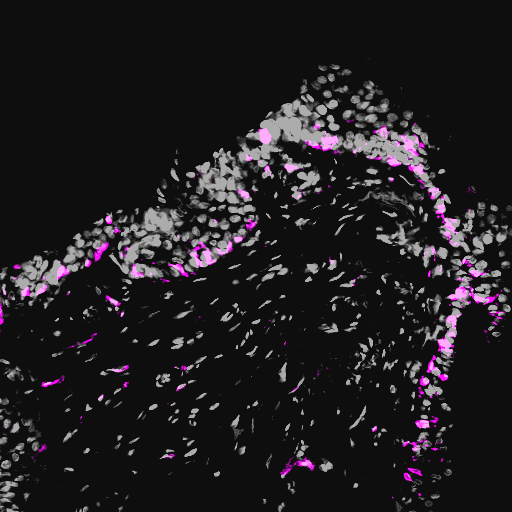

Supplement: Supplementary file 25 — Raw Image File 22 or Figure 4D [file 41467_2019_9834_MOESM25_ESM.tif]

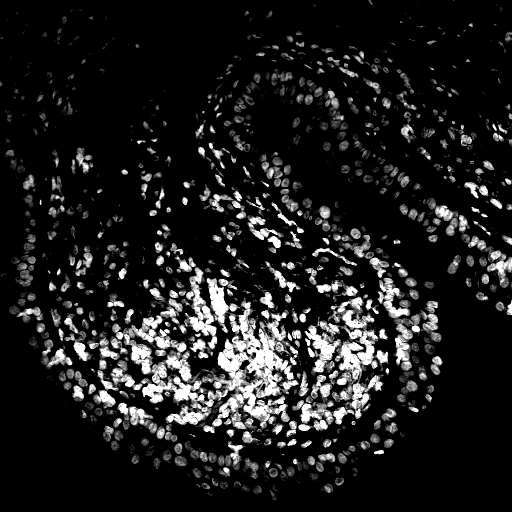

Supplement: Supplementary file 26 — Raw Image File 23 or Figure 4D [file 41467_2019_9834_MOESM26_ESM.tif]

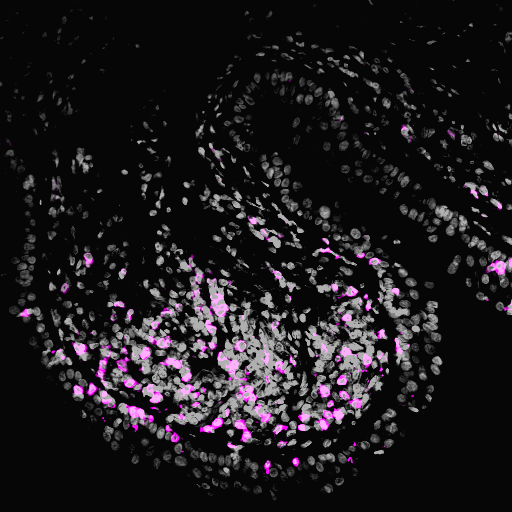

Supplement: Supplementary file 27 — Raw Image File 24 or Figure 4D [file 41467_2019_9834_MOESM27_ESM.tif]

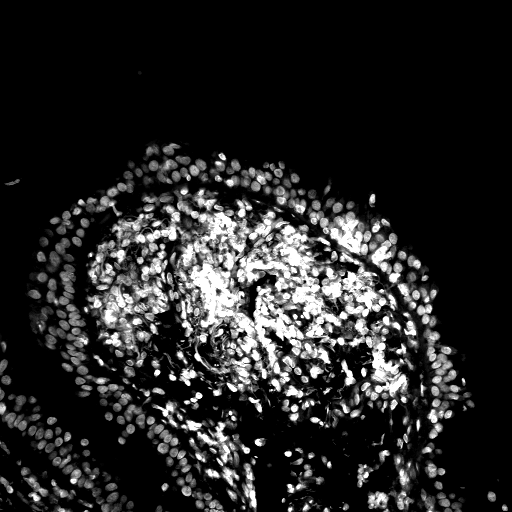

Supplement: Supplementary file 28 — Raw Image File 25 or Figure 4D [file 41467_2019_9834_MOESM28_ESM.tif]

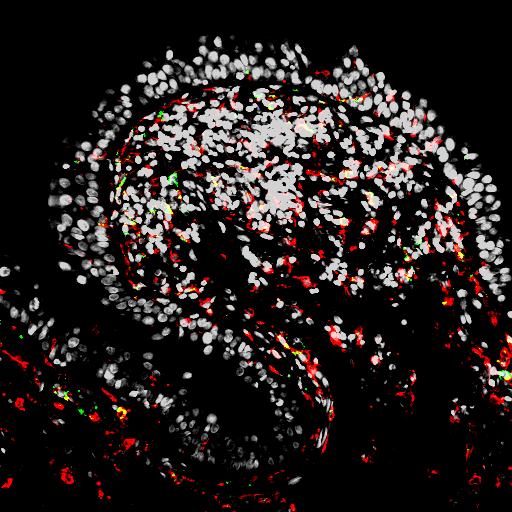

Supplement: Supplementary file 30 — Raw Image File 27 or Figure 4D [file 41467_2019_9834_MOESM30_ESM.tif]

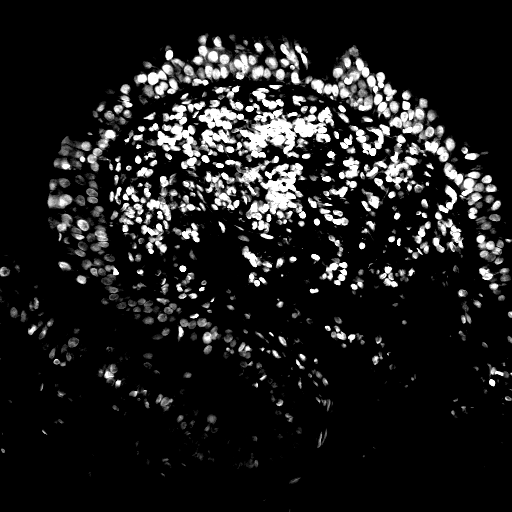

Supplement: Supplementary file 31 — Raw Image File 28 or Figure 4D [file 41467_2019_9834_MOESM31_ESM.tif]
