# Supplementary material for: Molecular subtyping reveals immune alterations associated with progression of bronchial premalignant lesions
Source: Nat Commun. 2019 Apr 23;10:1856. doi: 10.1038/s41467-019-09834-2 (PMC6478943; doi:10.1038/s41467-019-09834-2)
Supplement: Supplementary file 32 — Reporting Summary [file 41467_2019_9834_MOESM32_ESM.pdf]

nature research

Corresponding author(s): Jennifer Beane

Reporting Summary

Nature Research wishes to improve the reproducibility of the work that we publish. This form provides structure for consistency and transparency in reporting. For further information on Nature Research policies, see Authors & Referees and the Editorial Policy Checklist.

Statistical parameters

When statistical analyses are reported, confirm that the following items are present in the relevant location (e.g. figure legend, table legend, main text, or Methods section).

n/a	Confirmed	
☐	☒	The exact sample size (n) for each experimental group/condition, given as a discrete number and unit of measurement
☐	☒	An indication of whether measurements were taken from distinct samples or whether the same sample was measured repeatedly
☐	☒	The statistical test(s) used AND whether they are one- or two-sided<br>*Only common tests should be described solely by name; describe more complex techniques in the Methods section.*
☐	☒	A description of all covariates tested
☐	☒	A description of any assumptions or corrections, such as tests of normality and adjustment for multiple comparisons
☐	☒	A full description of the statistics including central tendency (e.g. means) or other basic estimates (e.g. regression coefficient) AND variation (e.g. standard deviation) or associated estimates of uncertainty (e.g. confidence intervals)
☐	☒	For null hypothesis testing, the test statistic (e.g. F, t, r) with confidence intervals, effect sizes, degrees of freedom and P value noted<br>*Give P values as exact values whenever suitable.*
☒	☐	For Bayesian analysis, information on the choice of priors and Markov chain Monte Carlo settings
☐	☒	For hierarchical and complex designs, identification of the appropriate level for tests and full reporting of outcomes
☒	☐	Estimates of effect sizes (e.g. Cohen's d, Pearson's r), indicating how they were calculated
☐	☒	Clearly defined error bars<br>*State explicitly what error bars represent (e.g. SD, SE, CI)*

Our web collection on statistics for biologists may be useful.

Software and code

Policy information about availability of computer code

Data collection	FASTQ files for RNA-Seq experiments were demultiplexed using Illumina CASAVA 1.8.2 or BaseSpace.<br>RNA-seq processing pipeline software for human data (https://github.com/joshua-d-campbell/nf-RNA_Seq_Preprocess v1.0): nextflow v0.24.4, star v2.5.2b, rsem v1.3.0, FastQC v0.11.3, Picard tools v2.8.0, GATK v3.5, rseqc v2.6.4, multiqc v0.9, samtools v1.4<br>RNA-seq processing software for mouse data: star v2.4.2a, samtools v1.2, picard v1.138, trimmomatic v0.33, rseqc v2.6.1, rsem v1.2.23
Data analysis	All custom computer codes are available upon request from the corresponding author. Custom scripts utilized the following software: R-3.3.2, GSVA v1.22.4, Limma v3.30.13, ggplot2 v3.0.0, SummarizedExperiment v1.4.0, edgeR v3.16.5, ConsensusClusterPlus v1.38.0, biomaRt v2.30.0, estimate v1.0.13, heatmap3 v1.2.2, pamr v1.55, lme4 v1.1-13

For manuscripts utilizing custom algorithms or software that are central to the research but not yet described in published literature, software must be made available to editors/reviewers upon request. We strongly encourage code deposition in a community repository (e.g. GitHub). See the Nature Research guidelines for submitting code & software for further information.

Data

Policy information about availability of data

All manuscripts must include a data availability statement. This statement should provide the following information, where applicable:

- Accession codes, unique identifiers, or web links for publicly available datasets
- A list of figures that have associated raw data
- A description of any restrictions on data availability

RNA sequencing data from human endobronchial biopsies and brushings has been deposited in the NCBI Gene Expression Omnibus under accession code GSE109743 [http://www.ncbi.nlm.nih.gov/geo/query/acc.cgi?acc=GSE109743]. RNA sequencing data from mouse lung samples treated with N-nitrosotris-(2-choroethyl)urea has been deposited in the NCBI Gene Expression Omnibus under accession code GSE111091 [http://www.ncbi.nlm.nih.gov/geo/query/acc.cgi?acc=GSE111091]. The source data underlying all figures and tables in the main text and supplementary information are provided as a Source Data file. All other data supporting the findings of this study are available within the article and its supplementary information files and from the corresponding author upon reasonable request. A reporting summary for this article is available as a Supplementary Information file.

Field-specific reporting

Please select the best fit for your research. If you are not sure, read the appropriate sections before making your selection.

☒ Life sciences ☐ Behavioural & social sciences ☐ Ecological, evolutionary & environmental sciences

For a reference copy of the document with all sections, see nature.com/authors/policies/ReportingSummary-flat.pdf

Life sciences study design

All studies must disclose on these points even when the disclosure is negative.

Sample size	Subjects were selected that had biopsies collected in repeat locations via serial bronchoscopies; however, after RNA isolation, samples from 3 subjects had a single biopsy and 1 subject had a single brushing. mRNA sequencing was performed on a discovery cohort (DC) of samples comprising of endobronchial biopsies and brushes collected between 2010 and 2012 (n=30 subjects, n=197 biopsies, and n=91 brushings). mRNA sequencing was subsequently performed on a validation cohort (VC) of samples comprising of endobronchial biopsies and brushes collected between 2012 and 2015 (n=20 subjects, n=111 biopsies, and n=49 brushings).
Data exclusions	Out of the 448 samples profiled, 432 passed quality metrics and were included in the analyses. Samples were excluded were sex annotation did not correlate with gene expression across CYorf15A, DDX3Y, KDM5D, RPS4Y1, USP9Y, and UTY (n=4 samples). Sample relatedness within a patient was confirmed using Peddy software(47). Samples with a high-rate of heterozygosity (more than 3 standard deviations above the median) or samples with low relatedness to samples from the same patient (more than 3 standard deviations below the median) were removed from further analyses (n=11 samples, 2 brushes and 9 biopsies). Samples were subsequently divided into the discovery and validation cohorts (as outlined above) and by tissue type (biopsy or brush). Subsequent sample and gene filtering was conducted separately on each set as follows: First, EdgeR was used to compute normalized data (library sizes normalized using TMM, trimmed mean of M-values, and log2 counts per million computed) and genes were excluded that either had an interquartile range equal to zero or a sum across samples equal or less than 1. Samples were excluded based on values greater than 2 standard deviations from the mean for more than one of the following criteria: 1) mean Pearson correlation with all other samples calculated across all filtered genes 2) the 1st or 2nd principal components calculated using the filtered gene expression matrix 3) transcript integrity number (TIN, computed by RSeQC).
Replication	Results presented were derived in a discovery cohort of samples and replicated in a validation cohort.
Randomization	The discovery and validation cohorts established based on when the samples were collected (see above).
Blinding	Blinding was not relevant to our study, as the goal was to discover biomarkers and potential therapeutics to detect and treat bronchial premalignant lesions. Future studies, where the biomarkers are rigorously tested will require blinding.

Reporting for specific materials, systems and methods

Materials & experimental systems	
n/a	Involved in the study
☐	☒ Unique biological materials
☐	☒ Antibodies
☒	☐ Eukaryotic cell lines
☒	☐ Palaeontology
☐	☒ Animals and other organisms
☐	☒ Human research participants

Methods	
n/a	Involved in the study
☒	☐ ChIP-seq
☒	☐ Flow cytometry
☒	☐ MRI-based neuroimaging

Unique biological materials

Policy information about availability of materials

Obtaining unique materials

All unique materials used are available from the authors of commercial sources (Qiagen, Illumina, Dako, Cell Marque,ThermoFisher, BioLegend, Abcam, Sigma).

Antibodies

Antibodies used

Antibody/Company/Catalog/Dilution/Antigen retrieval/Species

Immune cell type markers
CD68/Dako/m0876/1- 250/AR6/mouse
CD163/Cell Marque/163m-16/1-100/AR9/mouse
CD4/Thermo Fisher/ms1528S/1-100/AR9/mouse
CD8/Dako/M7103/1-100/AR9/mouse

Epithelial cell type and proliferation markers
Ac-a-Tub/Sigma/T6793/1-100/citrate/mouse
KRT5/BioLegend 905-901/1-100 citrate/chicken
KI67/Abcam/ab16667/1-100/citrate/rabbit

Validation

Antibodies were validated individually using tonsil tissue.

Animals and other organisms

Policy information about studies involving animals; ARRIVE guidelines recommended for reporting animal research

Laboratory animals

Female A/J and SWR/J mice from Jackson labs were purchased at ~6w. of age and entered into the study at ~ 8w. of age

Wild animals

This study did not involve wild animals.

Field-collected samples

This study did not involve did not involve the collection of field samples.

Human research participants

Policy information about studies involving human research participants

Population characteristics

Endobronchial biopsies and brushings were obtained from high-risk subjects undergoing lung cancer screening at approximately 1-year intervals by white light and auto-fluorescence bronchoscopy and computed tomography at Roswell. The bronchoscopy included visualization of the vocal cords, trachea, main carina, and orifices of the sub-segmental bronchi visible without causing trauma to the bronchial wall. All abnormal and suspicious areas are biopsied twice and the lung anatomic location is recorded (Fig. S10, Table S9). One biopsy was used for routine pathological evaluation and the other for molecular profiling. Additionally, a brushing was obtained from a normal appearing area of the left or right mainstem bronchus for research. Morphological criteria used to evaluate the biopsies are in accordance with World Health Organization (WHO) guidance. Eligibility for screening includes either a previous history of aerodigestive cancer and no disease at the time of enrollment or age greater than 50, a current or previous history of smoking for a minimum exposure of 20 pack-years and at least one additional risk factor including moderate chronic obstructive pulmonary disease (COPD) (defined as forced expiratory volume (FEV1) < 70%), confirmed asbestos related lung disease or a strong family history of lung cancer (at least 1-2 first degree relatives).

Recruitment

Patients that meet the eligibility criteria above are referred for lung cancer screening. The population is usually comprised of older adults with smoking histories.
